# Wikis, blogs and podcasts: a new generation of Web-based tools for virtual collaborative clinical practice and education

**DOI:** 10.1186/1472-6920-6-41

**Published:** 2006-08-15

**Authors:** Maged N Kamel Boulos, Inocencio Maramba, Steve Wheeler

**Affiliations:** 1 Faculty of Health and Social Work, University of Plymouth, Drake Circus, Plymouth, Devon PL4 8AA, UK; 2 Faculty of Education, University of Plymouth, Drake Circus, Plymouth, Devon PL4 8AA, UK

## Abstract

**Background:**

We have witnessed a rapid increase in the use of Web-based 'collaborationware' in recent years. These Web 2.0 applications, particularly wikis, blogs and podcasts, have been increasingly adopted by many online health-related professional and educational services. Because of their ease of use and rapidity of deployment, they offer the opportunity for powerful information sharing and ease of collaboration. Wikis are Web sites that can be edited by anyone who has access to them. The word 'blog' is a contraction of 'Web Log' – an online Web journal that can offer a resource rich multimedia environment. Podcasts are repositories of audio and video materials that can be "pushed" to subscribers, even without user intervention. These audio and video files can be downloaded to portable media players that can be taken anywhere, providing the potential for "anytime, anywhere" learning experiences (mobile learning).

**Discussion:**

Wikis, blogs and podcasts are all relatively easy to use, which partly accounts for their proliferation. The fact that there are many free and Open Source versions of these tools may also be responsible for their explosive growth. Thus it would be relatively easy to implement any or all within a Health Professions' Educational Environment. Paradoxically, some of their disadvantages also relate to their openness and ease of use. With virtually anybody able to alter, edit or otherwise contribute to the collaborative Web pages, it can be problematic to gauge the reliability and accuracy of such resources. While arguably, the very process of collaboration leads to a Darwinian type 'survival of the fittest' content within a Web page, the veracity of these resources can be assured through careful monitoring, moderation, and operation of the collaborationware in a closed and secure digital environment. Empirical research is still needed to build our pedagogic evidence base about the different aspects of these tools in the context of medical/health education.

**Summary and conclusion:**

If effectively deployed, wikis, blogs and podcasts could offer a way to enhance students', clinicians' and patients' learning experiences, and deepen levels of learners' engagement and collaboration within digital learning environments. Therefore, research should be conducted to determine the best ways to integrate these tools into existing e-Learning programmes for students, health professionals and patients, taking into account the different, but also overlapping, needs of these three audience classes and the opportunities of virtual collaboration between them. Of particular importance is research into novel integrative applications, to serve as the "glue" to bind the different forms of Web-based collaborationware synergistically in order to provide a coherent wholesome learning experience.

## Background

### Introduction and aims of this paper

Recent years have witnessed a growing interest in the latest generation of Web-based collaborationware (also known as Web 2.0 tools), namely wikis, blogs and podcasts, as evidenced by the growing number of publications on the subject [1-17], and the many examples of online health-related professional and educational services that have adopted the use these tools.

Wikis, blogs/photoblogs and podcasts (and its video incarnation, the vodcast) carry the potential of complementing, improving and adding new collaborative dimensions to the many Web-based medical/health education, CPD (Continuing Professional Development), and research services currently in existence. They offer many unique and powerful information sharing and collaboration features. They also afford users the added advantage of reducing the technical skill required to use these features, by allowing users to focus on the information and collaborative tasks themselves with few delivery obstacles [18]. Such technology is known as 'transparent technology' [19] in as much as the user is able to concentrate more on the learning task by 'seeing through' the technological environment they are immersed within.

This paper explores, with examples, some of the current uses of Web 2.0 tools in the education of medical/nursing students, the continuing professional development and education of healthcare professionals, and patient education (see also 'Additional file 1'). We then touch on the pedagogy underpinning these tools (see also 'Additional file 2'), and discuss some of their advantages and disadvantages. The paper concludes with a preliminary brainstorming about a research agenda and an invitation to medical and health educationalists/researchers to formally debate, investigate and report on the use and effectiveness of these tools in clinical education, in order to build the currently lacking evidence base about these tools *in the context of medical/health education*, and to ultimately compile and disseminate among the medical/health education community comprehensive evidence-based best practice guidelines and exemplars of the use of these tools in teaching and learning.

### A brief overview of the phenomenon of wikis, blogs and podcasts in online medical/health education and communities of practice

#### Wikis

A wiki (from the Hawaiian *wiki*, to hurry, swift) is a collaborative Web site whose content can be edited by anyone who has access to it [20]. Perhaps the best example of a wiki in action today is 'Wikipedia – The Free Encyclopedia' [21]. Wikis, and in particular Wikipedia, represent a promising principle that can significantly transform the Internet information age; they have greatly grown in popularity in recent months and years [17].

Special conferences have been and are being organized to discuss the interesting Web phenomenon of wikis. For example, the ACM (Association for Computing Machinery)-sponsored WikiSym 2005, the 2005 International Symposium on Wikis, 17–18 October 2005, San Diego, California, USA [22].

Wikis can be used as a source for obtaining information and knowledge, and also as a method of virtual collaboration, e.g., to share dialogue and information among participants in group projects, or to allow learners to engage in learning with each other, using wikis as a collaborative environment to construct their knowledge or to be part of a virtual community of practice (see 'Additional file 2').

Medical and health-related wiki examples include the Flu Wiki, which is intended to help local public health communities prepare for, and perhaps cope, with a possible (avian) influenza pandemic [18,23], and Ganfyd, an online collaborative medical reference that is edited by medical professionals and invited non-medical experts [24].

Wiki features include easy editing, versioning capabilities, and article discussions (see [25-27] and 'Additional file 1' for further details and screenshots).

#### Blogs

A related Web information sharing technology is the 'blog'. A blog (WeBLOG) is a Web site that contains dated entries in reverse chronological order (most recent first) about a particular topic [28]. Functioning as an online journal, blogs can be written by one person or a group of contributors. Entries contain commentary and links to other Web sites, and images as well as a search facility may also be included.

Because blogs engage people in knowledge sharing, reflection, and debate, they often attract a large and dedicated readership [29]. They can also engender the drawing together of small virtual groupings of individuals interested in co-constructing knowledge around a common topic within a community of practice.

Standard blog features include easy posting, archives of previous posts, and a standalone Web page for each post to the blog with a unique URL. The latter feature facilitates linking to and organising content within the same blog and from external sites [13]. Posting a clinical photo from a digital camera directly to a blog after optimisation and adding of a blogger's comments can also be made at the touch of a button using, for example, a free Google product called Picasa [30]. Moreover, the currently available 3G generation of mobile phones equipped with 2+ megapixel cameras can instantly post high resolution clinical photos to photoblogs/moblogs (mobile blogs) to a potentially world wide audience on the Web [31].

Medical blog examples include Clinical Cases and Images [32,33], and DIG@UTMB blog (Dermatology Interest Group at the University of Texas Medical Branch – Galveston Texas) [13,34] (see 'Additional file 1' for further blog examples, feature details, and screenshots).

#### Podcasts and m-Learning (mobile learning)

"Podcasting's essence is about creating content (audio or video – vodcasts) for an audience that wants to listen when they want, where they want, and how they want [35]." Users can listen to podcasts and watch vodcasts on their computer (e.g., using Windows Media Player), or download to portable MP3/MP4 players and listen/watch on the move/anywhere, which is perfect for the busy health professional.

Podcasts are already being used in medical school curricula [36]. Meng [37] describes many educational applications of podcasting and videocasting, including:

- Recordings of lectures for those students unable to attend the lecture in person;

- Audio recordings of textbook content by chapter allowing students to "read" or review texts while walking or driving to class (can be significant aid for auditory learners – see 'Additional file 2'); and

- Downloadable libraries of high resolution heart and respiratory sounds for medical students.

Meng's 'white paper' also contains excellent 'How to Podcast' and 'How to VODcast' sections [37]. Podcasts can be created from written text using text-to-speech synthesizer software, but better podcasts featuring real human voice and radio-style programmes are also available [38].

Podcasts use RSS (Really Simple Syndication), which is now natively supported by, and built into, the latest Windows Internet Explorer 7 (IE7) [39]. Users do not need a dedicated 'podcatcher program' if they are running IE7/Windows Vista. Mozilla Firefox, a popular Web browser that is free and Open Source, also supports RSS through its "live bookmarks" [40].

Medical and health-related podcast examples include the New York University ophthalmology CME (Continuing Medical Education) programs via podcast [41], the New England Journal of Medicine podcasts [42], McGraw-Hill's AccessMedicine podcasts [43], and John Hopkins Medicine Podcasts [44]. Health-related podcasts are also available for patients and the general public. The Arizona Heart Institute [45] and the Cleveland Clinic [46] offer video podcasts for healthcare professionals as well as for patients. The Denison Memorial Library at the University of Colorado at Denver and Health Sciences Center has compiled a useful Health/Medical Podcast Directory [47] (see also [48,49] and 'Additional file 1' for further details and screenshots).

## Discussion

### Underpinning pedagogy

The notion of 'anytime, anyplace' learning has been difficult to achieve but, recently, the advent of cheaper, better supported mobile, personal technology is making mobile learning (or m-Learning) more achievable and more ubiquitous (u-learning) than ever before. Students are now more mobile than ever, and often find themselves multi-tasking, working in part-time jobs, or located some distance from a parent institution on professional practice placement. A similar situation is faced by clinicians in remote and rural areas, who often lack training and proper academic support because of their geographic isolation from the large central hospitals and academic centres of excellence in the main cities. In such situations, students can feel pressurised, unsupported and socially isolated from tutors and peers [50], and may even become discouraged and drop out from the course [51]; professionally isolated clinicians may also lag behind in their CPD. In this context, quality learner support is vital, and social presence [52] becomes a highly desirable feature to embed within the delivery of any learning product.

Furthermore, previous studies into the impacts of e-Learning have highlighted a number of quality concerns [53] prompting calls for improved delivery to learners in terms of cost benefits and better learning outcomes [54]. Wheeler et al. [19] have argued that deeper engagement with learning objects and online discussion groups yields significant benefits for the development of professional practice.

Although the potential impact of wiki, blog and podcast technologies on higher education in the UK and elsewhere is immense, it is perhaps the combined use of the three applications as 'mind tools' [55] that may yield the most powerful learning experiences. According to Jonassen et al. 'mindtools' act as cognitive reflection and amplification tools, aiding the construction of meaning, through the act of self-design of knowledge databases [55]. Wikis in particular, and blogs to a lesser extent, enable such activities, and actively involve learners in their own construction of knowledge.

The uses of such technologies to encourage learners' deeper engagement with learning materials, and the affordance of shared working spaces to improve collaboration between learners are desirable outcomes. It is generally held by many educators that students of all ages learn best when immersed within a culturally and socially rich environment in which scaffolding of learning can be achieved [56]. Further, where learners and peers are committed to achieving the same goals, they tend to regulate each other's performances [55], a positive outcome that can be facilitated through the use of shared, digital learning environments. The combination of wikis, blogs and podcasting technologies, then, has the potential to both liberate and tie learners together [55], creating dynamic learning communities.

However, as research has already shown, technology is neutral until it delivers content [57] and will lose its effectiveness if it is not applied in a planned and systematic manner [58]. It will, therefore, be important to effectively demonstrate how tutors successfully deploy such technologies in live learning contexts, and how dynamic content can be developed, edited, reused, and negotiated within a virtual community of professional practice [59]. It may also be necessary to re-educate learners regarding their participation within such a dynamic learning environment, for as Jonassen and his colleagues suggest, old models of education have left their legacy. Many students have been so busy memorising what teachers tell them, they may need some support when they first attempt to communicate with others using collaborative technologies [55].

'Additional file 2' provides further important insight into the underpinning pedagogy of Web 2.0 tools, their uses and best practices in the context of higher education, together with a comprehensive Webliography about the subject. (See also 'What's next? A research and development agenda' below.)

### General advantages, disadvantages and remedies

#### Advantages

Perhaps the two main big advantages of wikis, blogs and podcasts are their ease of use and the availability of many Open Source/free or low-cost software and hosting options to run them. Examples of the latter include MediaWiki (Open Source – the same software package that runs Wikipedia) [60] and Google Blogger (free) [61].

Like podcasts, wikis and blogs also use RSS, which means users can easily set up/subscribe to 'feeds' to automatically receive content updates from their favourite services.

Podcasts also have the potential of offering superior support for auditory learners (it is claimed that the primary learning style in at least 30% of learners is auditory [62,63] – see also 'Additional file 2'), and also for visual learners in case of vodcasts. However, audio and video files can be large in size; users must have sufficient bandwidth to download them.

#### Disadvantages

Wikis and blogs are sometimes prone to vandalism [64,65] and, as a result, to serious quality issues, because of their free form nature and the (relative/potential) lack of control over their content, though this can also be their very strength [66]. One of the most famous documented examples of Web vandalism occurred on Wikipedia in the biographical article about John Seigenthaler, Sr. [67].

In an open and collaborative Web environment, anyone can very easily post copyrighted material without the permission of copyright holders (see, for example, Wikipedia's regularly updated listings of possible copyright violations [68]), post otherwise unsuitable or misleading content, edit existing content in a way that reduces its quality/accuracy, or even delete/blank a good wiki entry. There is also the problem of protecting patient anonymity when clinical data and images are posted on the Web.

However, most good wiki software includes a restoration/rollback function, which allows the Administrator/editor to revert a page back to its latest non-vandalised version. And of course, copyrighted/patient material posted without permission can be edited out, when brought to the editors' attention (see also 'Remedies' below).

The lack of vital article meta-information is another potentially serious issue. Wikis are authored by communities, not individuals (open editing/distributed page authorship and ownership), and thus discourage the feeling of authorship. It is usually impossible to properly identity contributors to a wiki entry since wiki authors are typically anonymous, unless the group of contributors is extremely limited and/or authorial identification is enforced (but this latter option might deplete a wiki of one of its most important ingredients of strength) [69]. All what one usually finds in wikis are IP addresses and nicknames of authors and editors. The lack of clear and complete authorship/editorship information attached to each wiki entry, including authors'/editors' affiliations and credentials, is a very serious quality issue encountered in most wiki-based encyclopaedias these days.

Wiki author anonymity also poses enormous questions for higher education institutions where assessment and grading are still typically based on individual efforts [69].

On the other hand, it is this very openness of wikis that gives rise to the concept of "Darwikinism" [66], which is a concept that describes the "socially Darwinian process" that wiki pages are subject to. Basically, because of the openness and rapidity that wiki pages can be edited, the pages undergo an evolutionary selection process not unlike that which nature subjects to living organisms. "Unfit" sentences and sections are ruthlessly culled, edited and replaced if they are not considered "fit", which hopefully results in the evolution of a higher quality and more relevant page. Whilst such openness may invite "vandalism" and the posting of untrue information, this same openness also makes it possible to rapidly correct or restore a "quality" wiki page.

In fact, a recent review of Wikipedia vis-á-vis the online Encyclopaedia Britannica showed that similar amounts of errors were found in both online encyclopaedias, indicating that the quality of articles in Wikipedia approached that of the Encyclopaedia Britannica [70].

#### Remedies

##### Monitoring and moderation of open wikis and blogs

What follows is an approach to the management of content adopted by Wikipedia [21]. Monitoring and moderating posts, and deleting/reverting back edits (rollback function) as necessary; protecting (rendering 'read-only') key/stable content; controlling who can post; blocking specific (problematic) users/IP addresses are all possible remedies in an open wiki or blog (where anyone can edit). Wiki and blog software packages have built-in Administrator's functionalities to support these tasks. However, monitoring, moderation and administration tasks can be very time-consuming due to the requirement for intensive human resourcing, and may prove to be too great a challenge to ask of educators who already lack time and resources [69,71,72].

##### The 'closed environment' scenario

Another alternative approach is what these authors call the 'closed environment' scenario. Perhaps the best example of such a closed environment is Ganfyd [24,73]. In this scenario, the wiki or blog owner(s)/Administrator(s) enforce, check, and limit wiki and blog registration and editing privileges to selected, well-defined, and verifiable special interest groups or communities of users. Posting/editing articles on these wikis and blogs will thus be limited to select, well-known and trustworthy people (the Administrator may also ask them to create an online user profile detailing their institutional affiliation and credentials). Everyone else will still be able to access/read the Wiki or blog and, if desirable/required, also post limited (moderated) comments (to build a community). (Read-only access and posting limited moderated comments/discussion topics can also be blocked by the Administrator, if deemed necessary.) Once a trustworthy expert is identified from among external readers (based on the quality of his/her posted comments and further private communication with them), they can also be granted posting/editing privileges (and in this way the (closed) pool of editors will keep growing).

This scenario would be suitable for wikis of the kind proposed by Wang [2]. Wang's gene-function wiki aims at utilizing the collective knowledge and intelligence of biologists around the world to create an invaluable tool for biological sciences. Wang postulates that such a Wiki would also be less susceptible to spam and more accurate, as most editors would be (verified) biologists.

##### Patient privacy

Patient permission must be sought when posting clinical photos and videos, and all efforts should be made to preserve individuals' privacy, e.g., by reasonably de-identifying face images. Clinical blogs/photoblogs, wikis and podcasts/vodcasts may also be password protected if necessary to further preserve patient confidentiality [74]. The ease of use of the wiki/blog software also makes it a simple matter for an editor to delete/revert or modify material that violates patient privacy.

##### Towards a research and development agenda

Clearly then, these Web 2.0 applications are here to stay and can be of great use in the higher education, CPD, and patient education settings. However, new technologies are particularly vulnerable to criticism as they can be costly to deploy/employ (not just the software cost), time consuming to learn to use (e.g., for tutors to develop pedagogically sound 'use scenarios' and activities that make use of the new technologies), and may initially demonstrate little pertinence for teaching and learning. Emerging technologies such as those introduced in this article should therefore be systematically evaluated to ascertain their benefits and limitations in a number of learning contexts, and to determine and document their proper use for higher education, the CPD of healthcare professionals, and patient education.

Undergraduate and postgraduate students, clinicians in practice, and members of the general public/patients are in many ways different audiences with different learning needs. However, there are also many areas of overlap and potentials for useful online collaboration between these audience. There might be some room for compiling some shared-audience educational content sets using Web 2.0 tools, and, in doing so, maximising the efficiencies of content authoring and delivery, and promoting fruitful collaboration between students, clinicians and patients. However, in order to achieve this, research is needed into which factors (in relation to content, presentation form and audience) make the intersection between the different audience domains grow big or small, and into the different possibilities/scenarios for collaboration between these audiences.

Research into the use and evaluation of Web 2.0 tools *in medical/health education *is still in its infancy, and the current pedagogic evidence base about these tools *in the context of medical/health education *is seriously lacking. We would therefore like to invite educators/researchers to experiment with these tools in some formal way and report back their results to the medical/health education community, so that we may start building a proper evidence base, e.g., about best practices/uses of these tools in medical/health teaching and learning, and for different audiences.

In her paper on wiki pedagogy published in 2005, Renée Fountain provides a comprehensive list of "wiki issues that pose fundamental – if not radical – questions for higher education, and, as such, merit considerable investigation" [69]. There is an abundance of "trade magazine-style articles" out there today about Web 2.0 tools, but very little reliable original pedagogic research and evaluation evidence to properly and fully answer this sort of questions. Research into analysing the uses, benefits and limitations of Web 2.0 learning solutions should therefore be a priority for universities that adopt such technologies. User perspectives of both student and tutor can proffer different, yet complementary, vital insights into the effectiveness of learning technologies within variable contexts, and should therefore be adequately covered in any pedagogic research into Web 2.0 tools.

In an article published in 2006, Whitsed suggests building a 'technology lab' for further experimentation and research into wikis, blogs and related tools in higher education [75]. Clearly, an adequate user base must be present in order to be able to properly experiment with, and evaluate Web 2.0 applications. The proposed 'technology labs' would be a good means to invite and encourage large numbers of medical/health educators, practitioners, and their institutions to start using and innovating with these tools.

**Figure 1:**
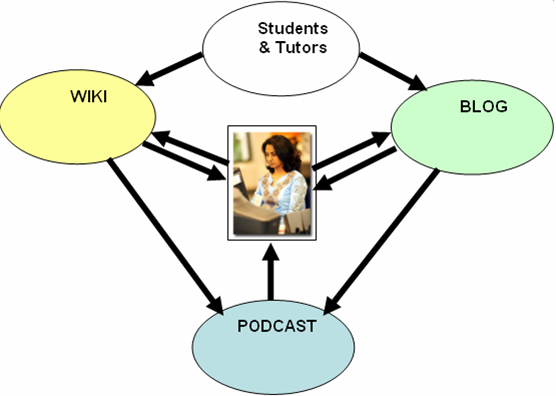
**The co-relationship and dependent positioning of wikis, blogs and podcasts within a student centred learning environment**. A diagram to indicate the co-relationship, dependent positioning, and potential for confluence of the three collaborationware components discussed in this paper, wikis, blogs and podcasts, within a student centred learning environment. The diagram illustrates the flow paths of communication.

Studies could also investigate the cross-operability and integration (confluence) of the three emerging Web 2.0 applications (wikis, blogs and podcasts), and their respective and synergistic contributions toward the enhancement of student learning (Figure 1). Building on these studies, researchers could establish key activities that can be evidenced to enhance student learning experiences and deepen levels of student engagement within digital learning environments.

It is noteworthy that some confluence examples of Web 2.0 tools already exist, or have recently been proposed, e.g., blikis (blogs with wiki support [76]). Confluence of Web 2.0 tools is usually sought for synergy, new unique hybrid features, and/or convenience (see 'Additional file 2' for more details and examples).

## Summary and conclusion

The latest generation of collaborative Web-based tools, namely wikis, blogs/photoblogs, blikis and podcasts/vodcasts, offer many unique and powerful information sharing and collaboration features. In this paper we have explored how these Web 2.0 applications would prove useful on the long run for virtual collaborative clinical practice and learning, based on the currently available initial online medical/health-related examples and literature about these tools. Careful thinking and research are still needed in order to find the best ways to leverage these emerging tools to boost our teaching and learning productivity, foster better 'communities of practice', and support continuing medical education/professional development (CME/CPD) and patient education.

Stakeholders'/prospective users' representatives (students, healthcare professionals and patients) must be adequately involved in these research and development processes.

## Competing interests

The author(s) declare that they have no competing interests.

## Authors' contributions

MNKB conceived and drafted this manuscript and the two accompanying multimedia PowerPoint presentations. IM contributed important and unique insight to the article and wrote parts of it. SW contributed toward the pedagogical conceptualisation and theorisation of the article. All authors read and approved the final manuscript.

## Pre-publication history

The pre-publication history for this paper can be accessed here:



## Supplementary Material

Additional File 1 **Wikis, blogs and podcasts: emerging tools for virtual collaborative practice and learning/CPD in medicine**. An illustrated PowerPoint introducing wikis, blogs and podcasts, and their use in medical education and practice, with pointers to some online medical and health-related examples of these tools (format: PPT). (Note: slide 35 has embedded sound; please turn on your speakers.) Click here for file

Additional File 2 **Web 2.0 tools: underpinning pedagogy, uses, and best practices in education**. An illustrated PowerPoint PDF introducing the underpinning pedagogy of Web 2.0 tools, their uses and best practices in the context of higher education, together with a comprehensive Webliography about the subject. The presentation covers wikis, blogs, wikis vs. blogs, podcasts, and confluence of Web 2.0 tools, e.g., blikis and voice wikis (format: PDF). Click here for file
