# Supplementary material for: Wikis, blogs and podcasts: a new generation of Web-based tools for virtual collaborative clinical practice and education
Source: BMC Med Educ. 2006 Aug 15;6:41. doi: 10.1186/1472-6920-6-41 (PMC1564136; doi:10.1186/1472-6920-6-41)
Supplement: Additional File 1 — Wikis, blogs and podcasts: emerging tools for virtual collaborative practice and learning/CPD in medicine. An illustrated PowerPoint introducing wikis, blogs and podcasts, and their use in medical education and practice, with pointers to some online medical and health-related examples of these tools (format: PPT). (Note: slide 35 has embedded sound; please turn on your speakers.) [file 1472-6920-6-41-S1.ppt]

## Slide 1
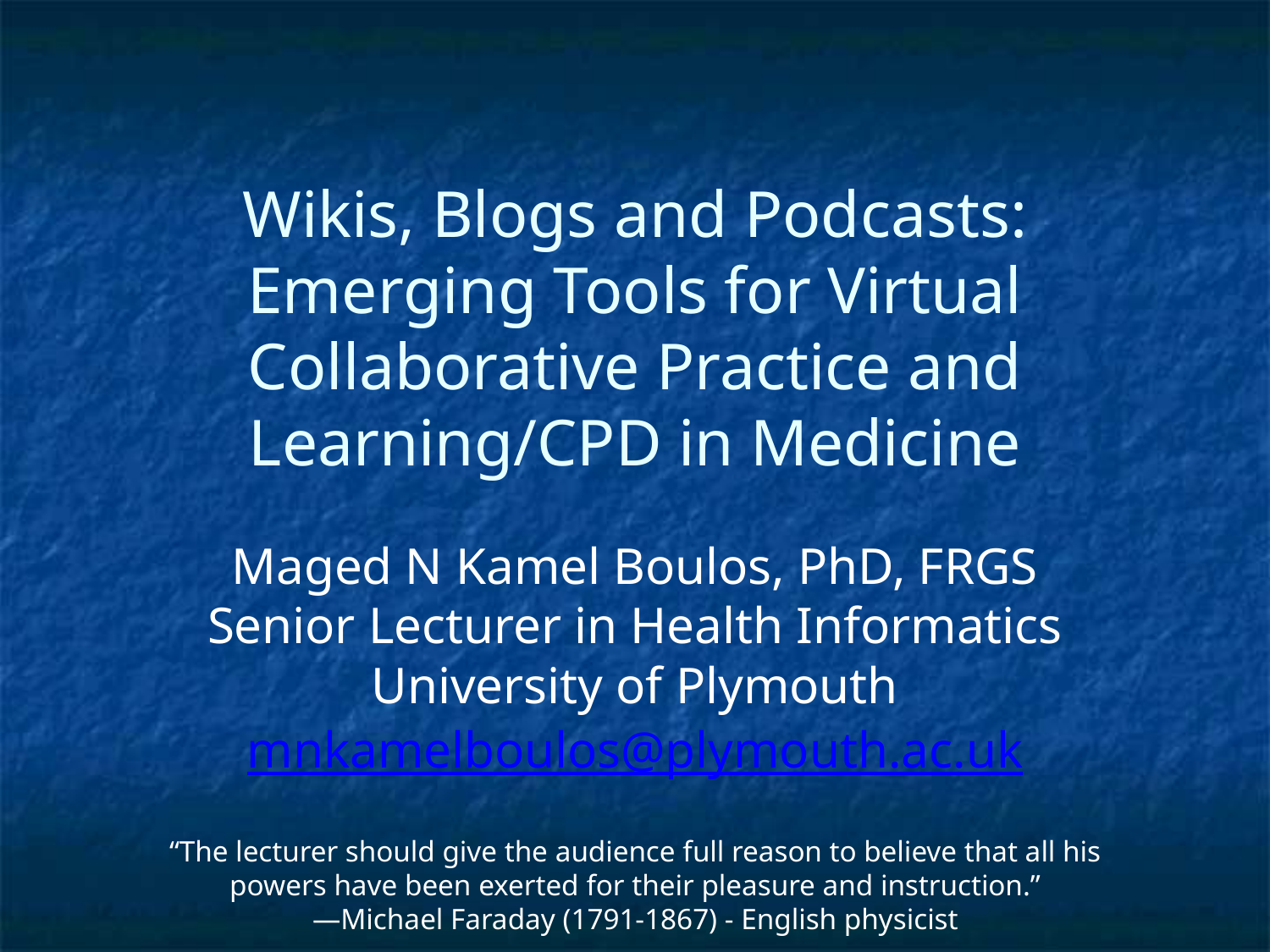

# Wikis, Blogs and Podcasts: Emerging Tools for Virtual Collaborative Practice and Learning/CPD in Medicine
Maged N Kamel Boulos, PhD, FRGS
Senior Lecturer in Health Informatics
University of Plymouth
mnkamelboulos@plymouth.ac.uk
“The lecturer should give the audience full reason to believe that all his powers have been exerted for their pleasure and instruction.”
—Michael Faraday (1791-1867) - English physicist

## Slide 2
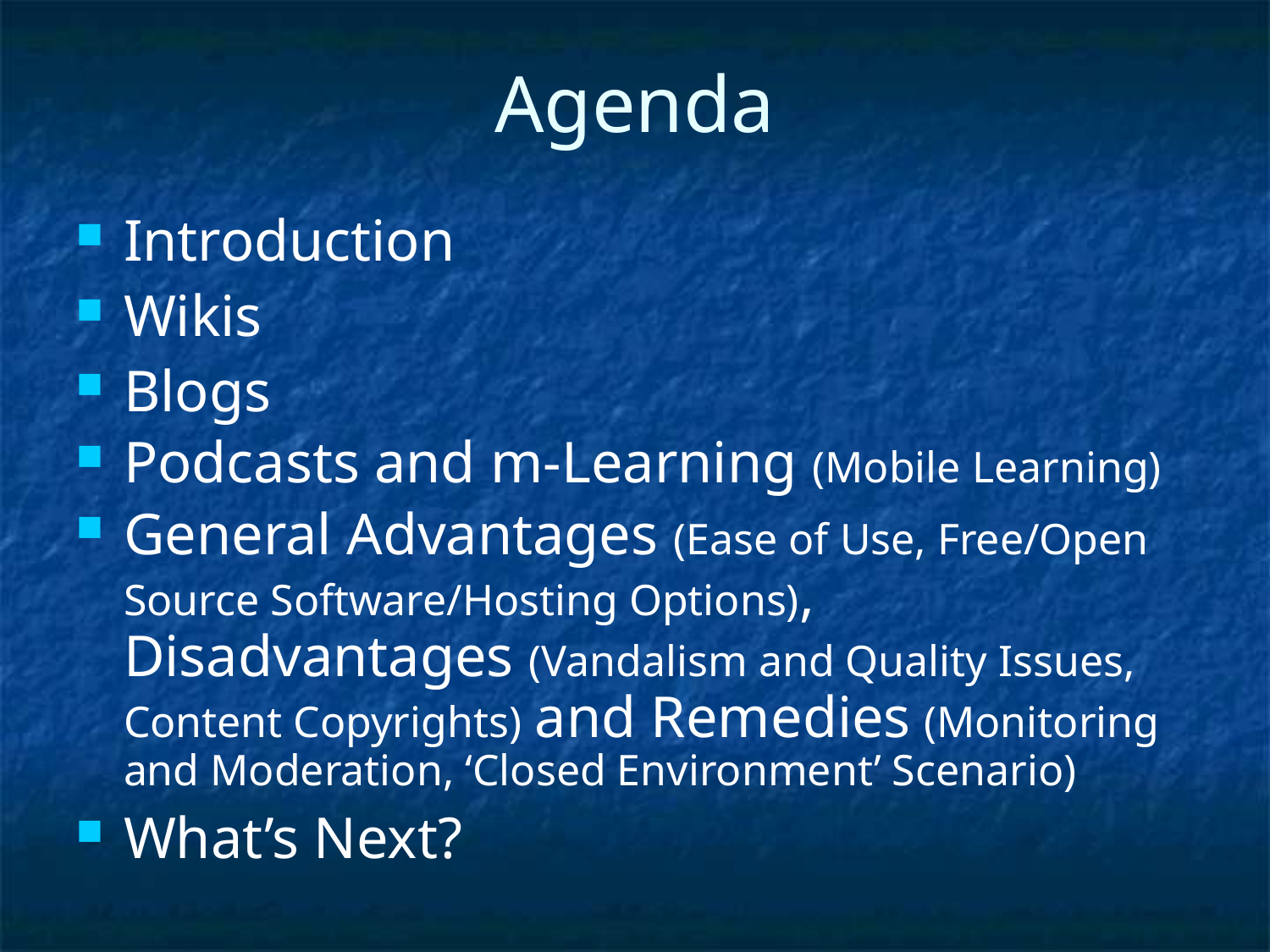

# Agenda
Introduction
Wikis
Blogs
Podcasts and m-Learning (Mobile Learning)
General Advantages (Ease of Use, Free/Open Source Software/Hosting Options), Disadvantages (Vandalism and Quality Issues, Content Copyrights) and Remedies (Monitoring and Moderation, ‘Closed Environment’ Scenario)
What’s Next?

## Slide 3
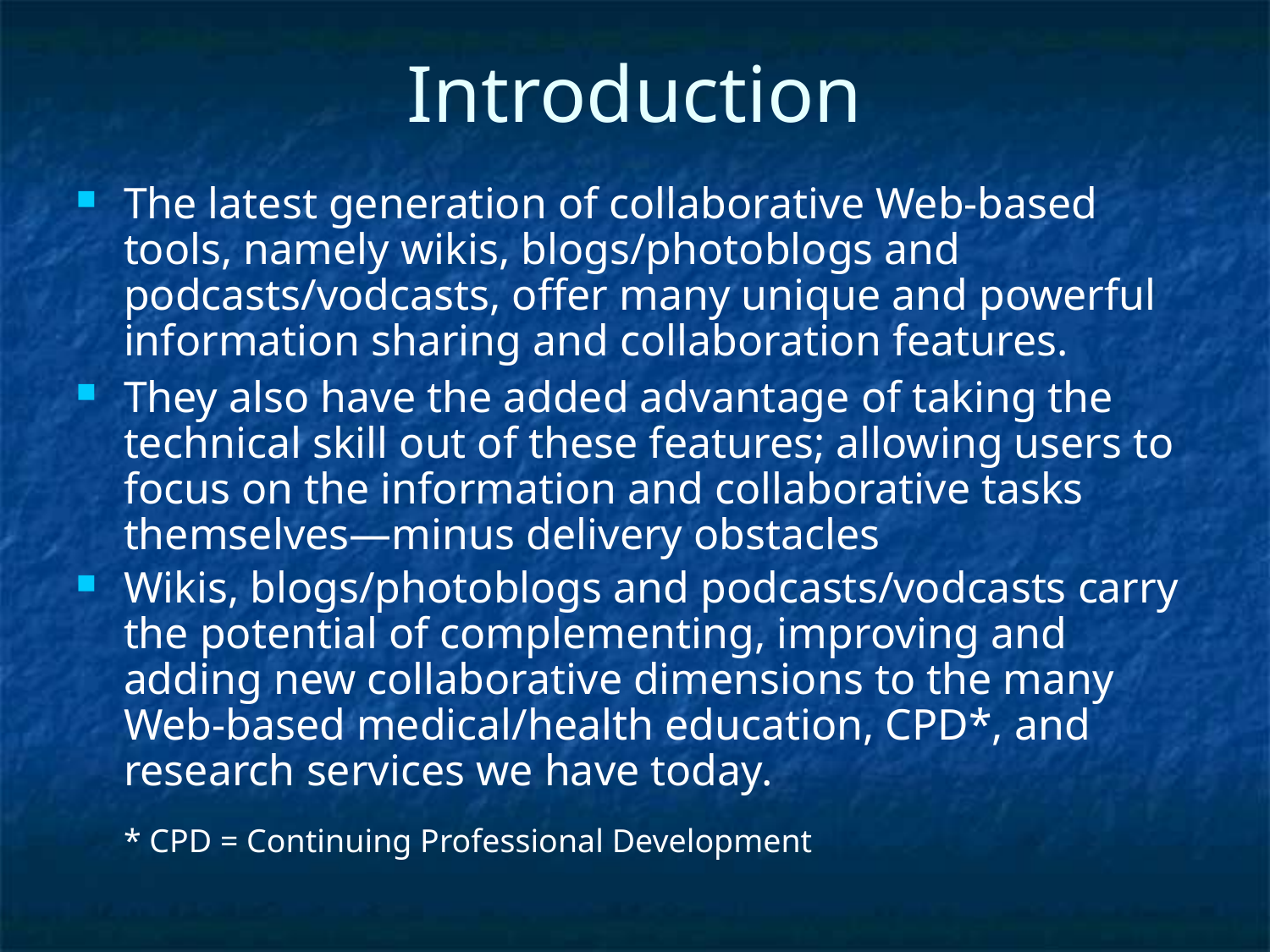

# Introduction
The latest generation of collaborative Web-based tools, namely wikis, blogs/photoblogs and podcasts/vodcasts, offer many unique and powerful information sharing and collaboration features.
They also have the added advantage of taking the technical skill out of these features; allowing users to focus on the information and collaborative tasks themselves—minus delivery obstacles
Wikis, blogs/photoblogs and podcasts/vodcasts carry the potential of complementing, improving and adding new collaborative dimensions to the many Web-based medical/health education, CPD*, and research services we have today.
* CPD = Continuing Professional Development

## Slide 4
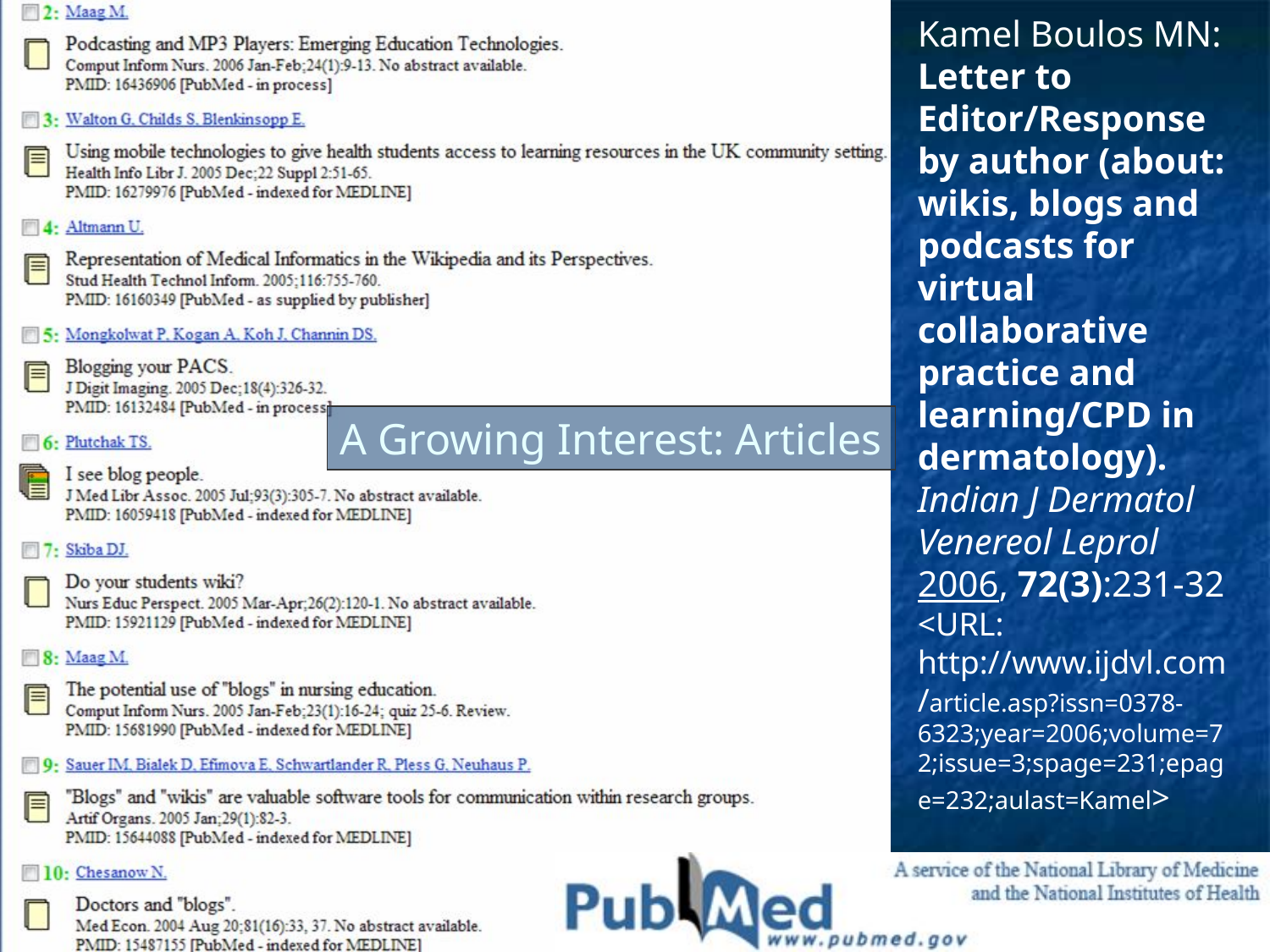

Kamel Boulos MN: Letter to Editor/Response by author (about: wikis, blogs and podcasts for virtual collaborative practice and learning/CPD in dermatology). Indian J Dermatol Venereol Leprol 2006, 72(3):231-32 <URL: http://www.ijdvl.com/article.asp?issn=0378-6323;year=2006;volume=72;issue=3;spage=231;epage=232;aulast=Kamel>
A Growing Interest: Articles

## Slide 5
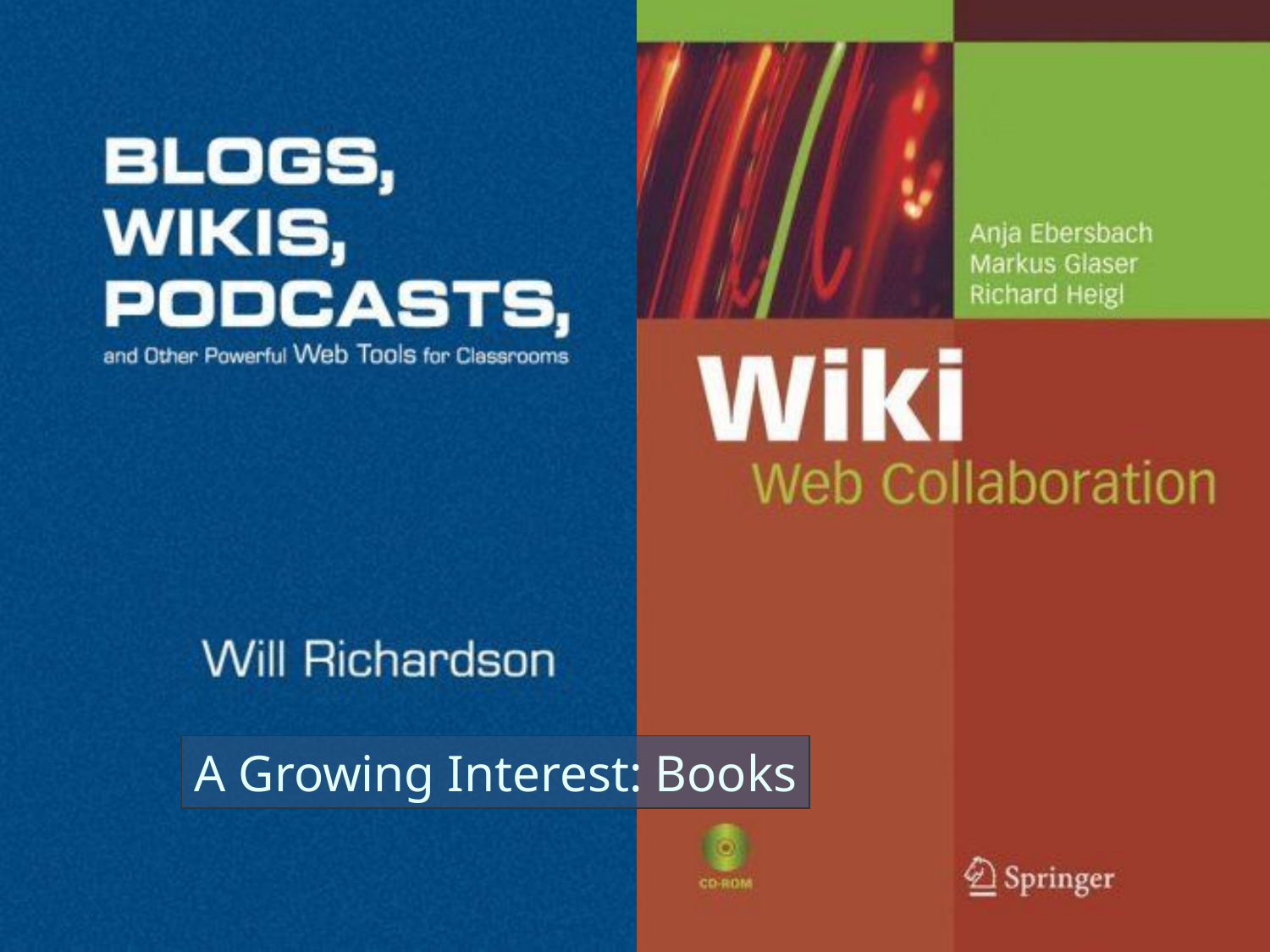

A Growing Interest: Books

## Slide 6
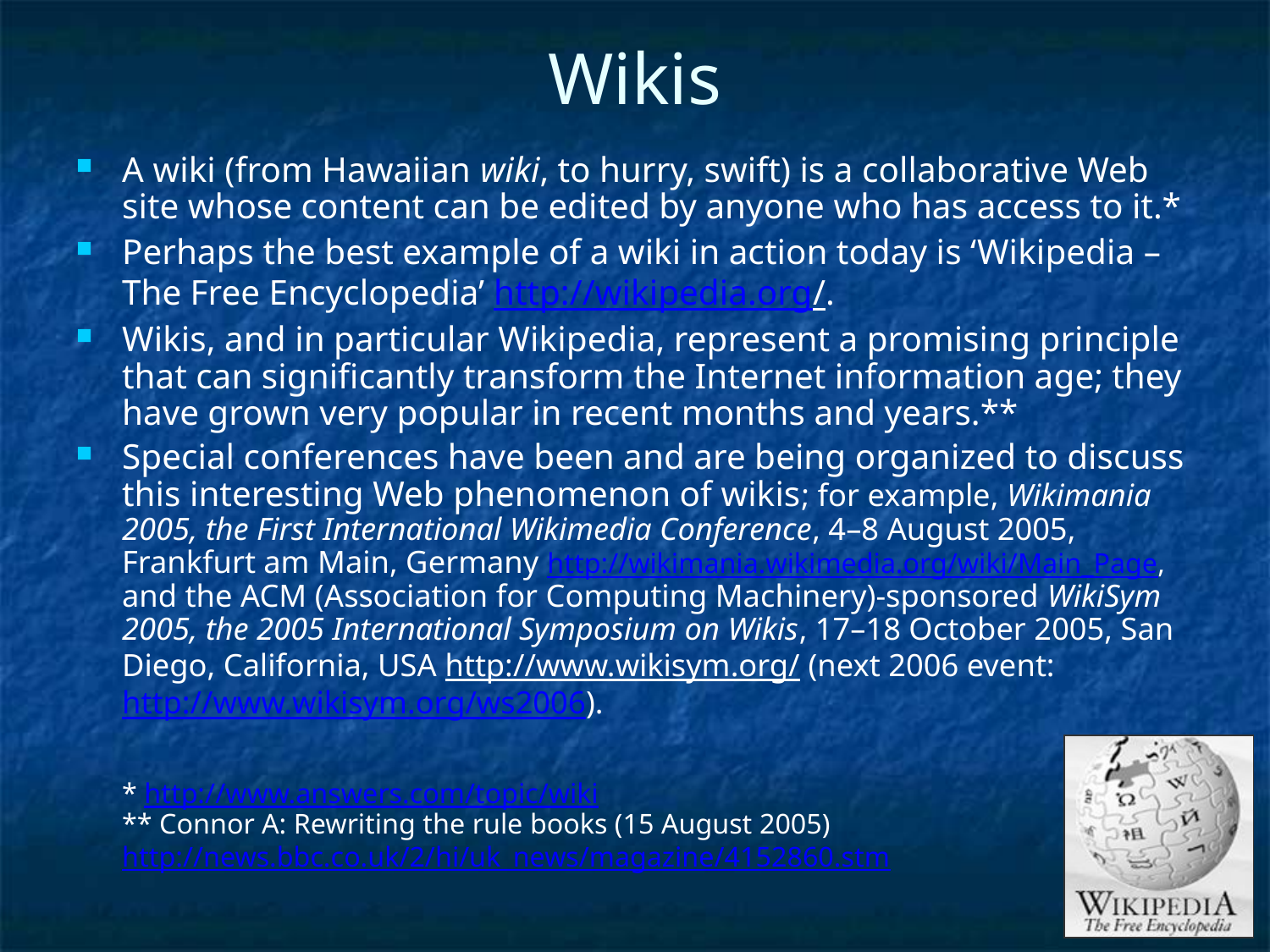

# Wikis
A wiki (from Hawaiian wiki, to hurry, swift) is a collaborative Web site whose content can be edited by anyone who has access to it.*
Perhaps the best example of a wiki in action today is ‘Wikipedia – The Free Encyclopedia’ http://wikipedia.org/.
Wikis, and in particular Wikipedia, represent a promising principle that can significantly transform the Internet information age; they have grown very popular in recent months and years.**
Special conferences have been and are being organized to discuss this interesting Web phenomenon of wikis; for example, Wikimania 2005, the First International Wikimedia Conference, 4–8 August 2005, Frankfurt am Main, Germany http://wikimania.wikimedia.org/wiki/Main_Page, and the ACM (Association for Computing Machinery)-sponsored WikiSym 2005, the 2005 International Symposium on Wikis, 17–18 October 2005, San Diego, California, USA http://www.wikisym.org/ (next 2006 event: http://www.wikisym.org/ws2006).
* http://www.answers.com/topic/wiki
** Connor A: Rewriting the rule books (15 August 2005) http://news.bbc.co.uk/2/hi/uk_news/magazine/4152860.stm

## Slide 7
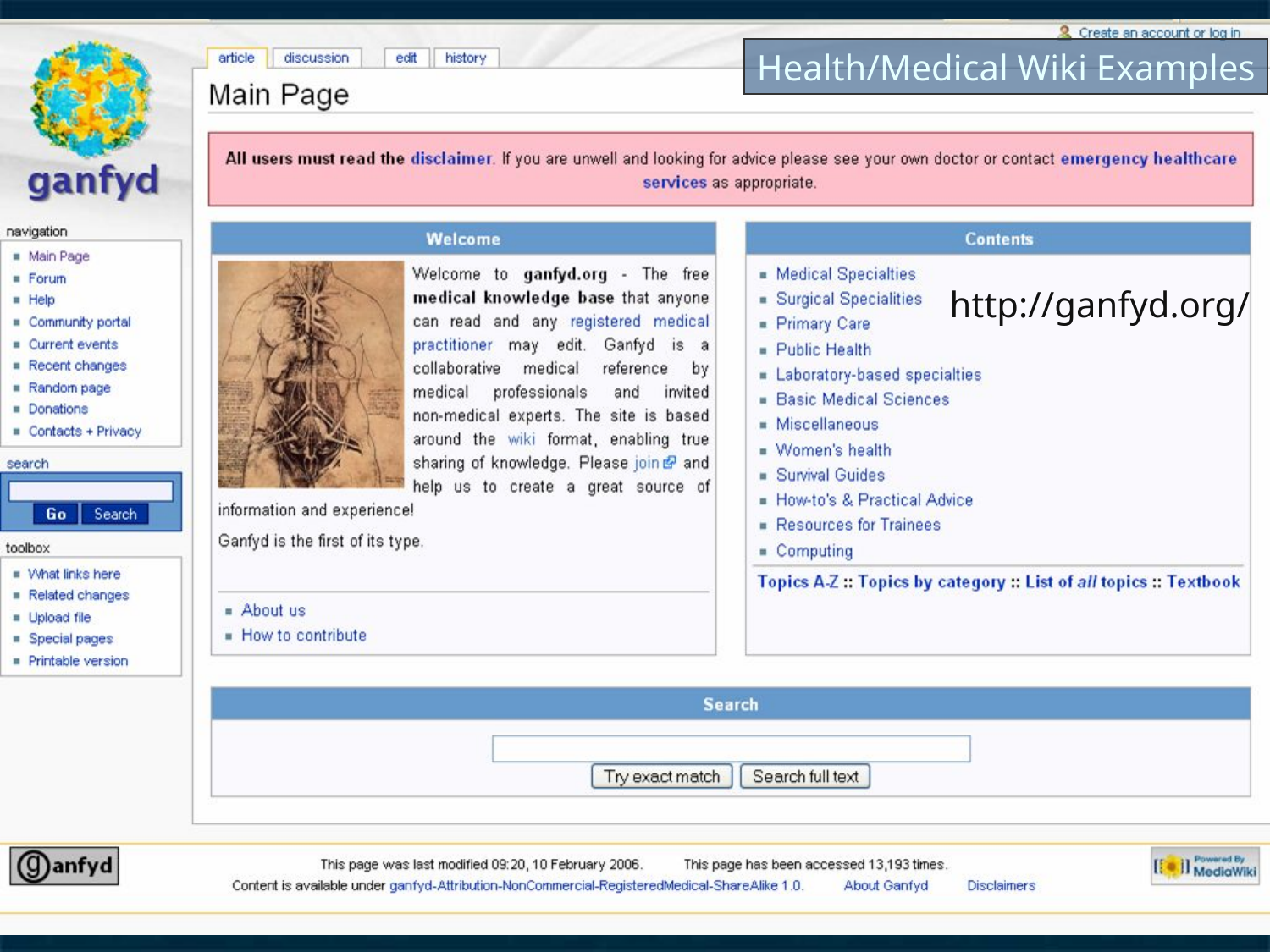

Health/Medical Wiki Examples
http://ganfyd.org/

## Slide 8
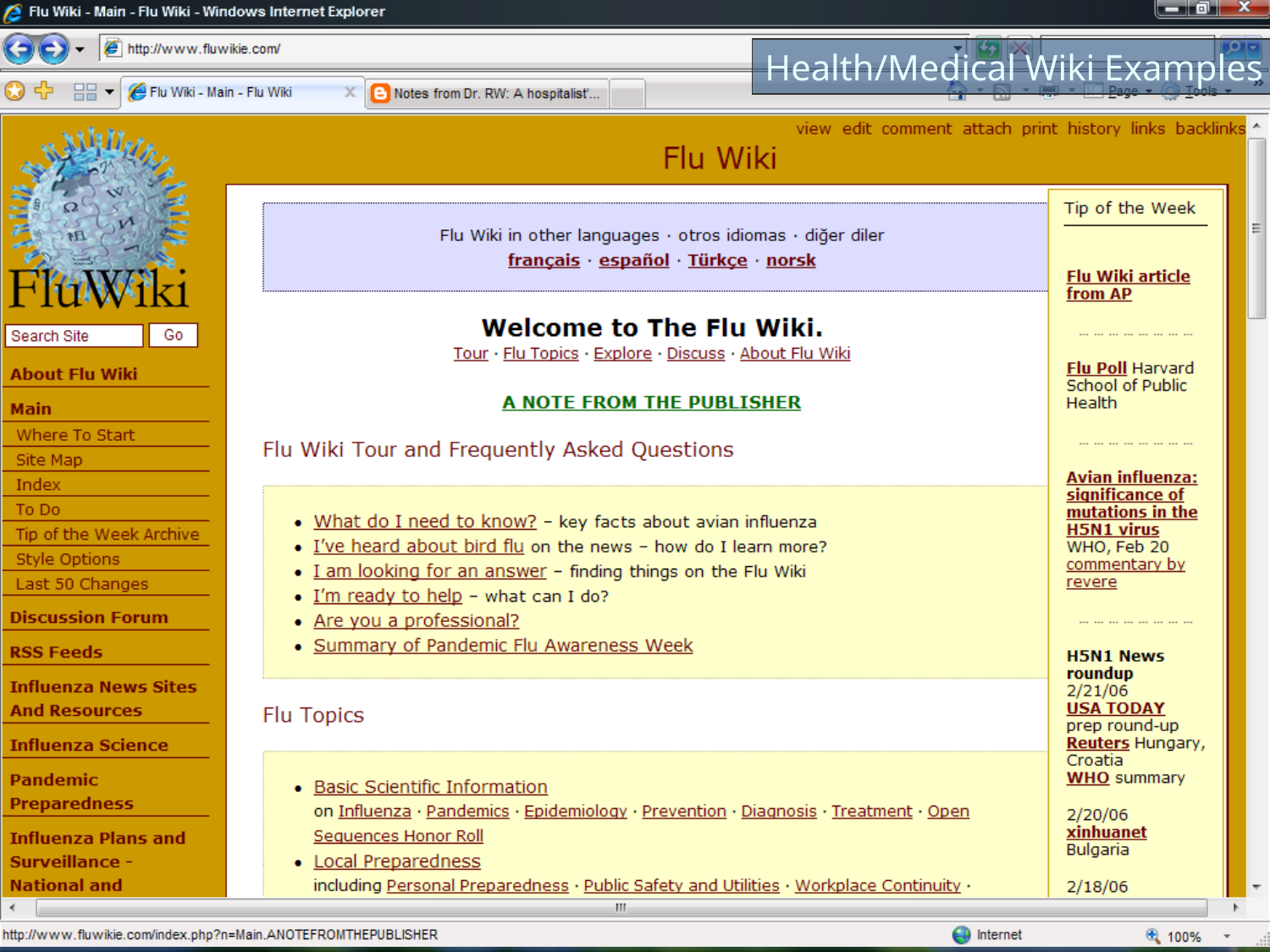

Health/Medical Wiki Examples

## Slide 9
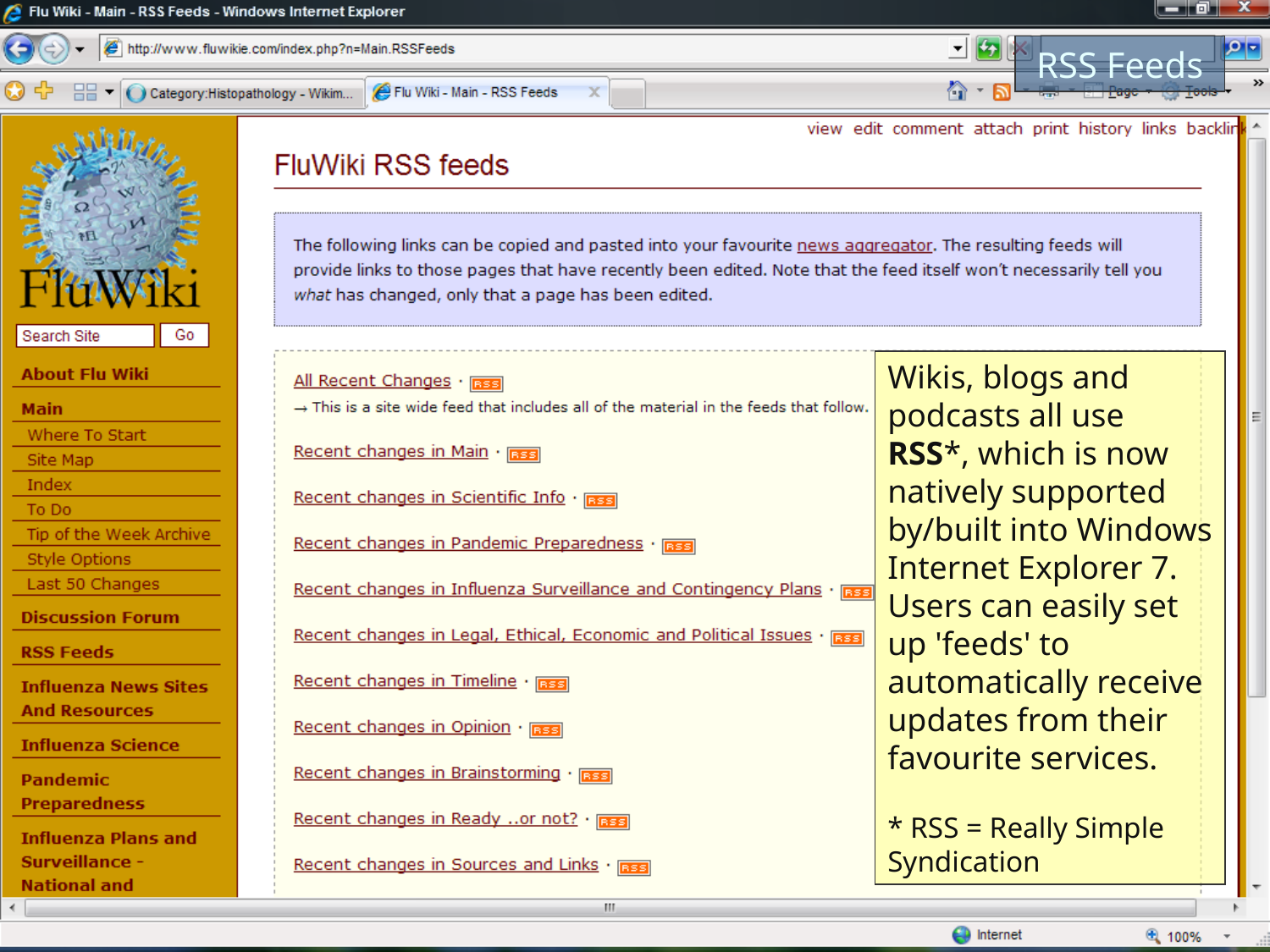

RSS Feeds
Wikis, blogs and podcasts all use RSS*, which is now natively supported by/built into Windows Internet Explorer 7. Users can easily set up 'feeds' to automatically receive updates from their favourite services.
* RSS = Really Simple Syndication

## Slide 10
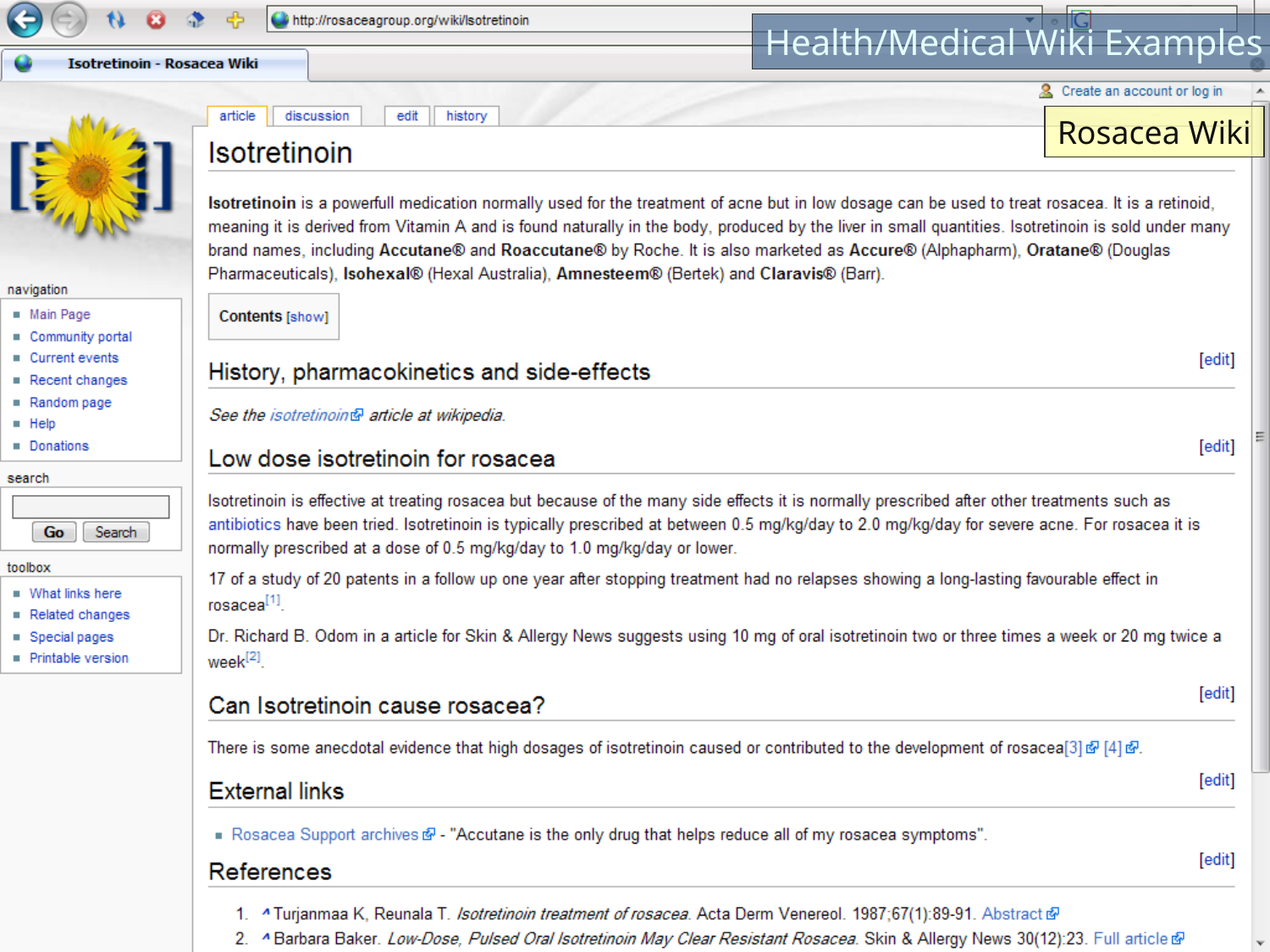

Health/Medical Wiki Examples
Rosacea Wiki

## Slide 11
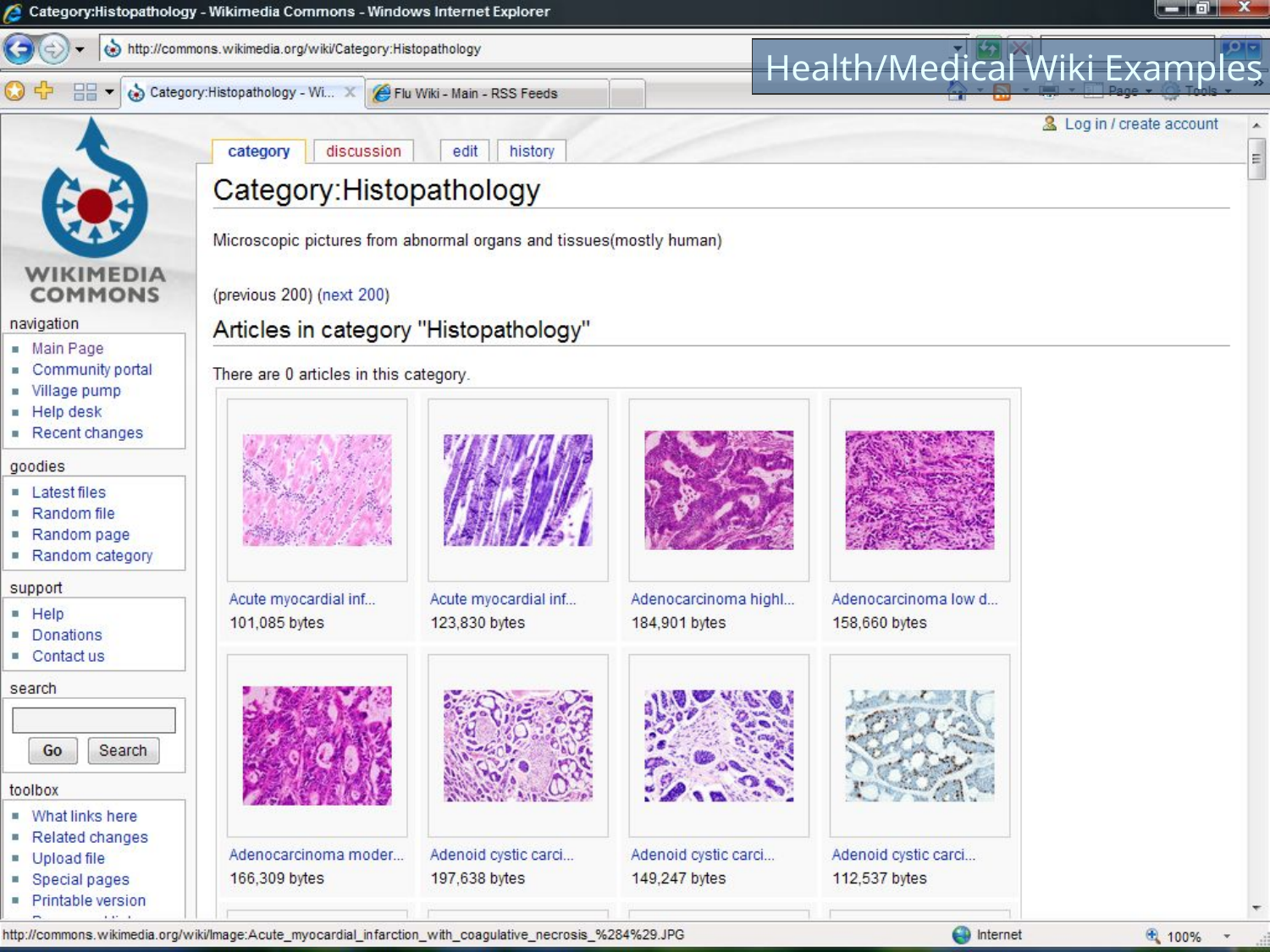

Health/Medical Wiki Examples

## Slide 12
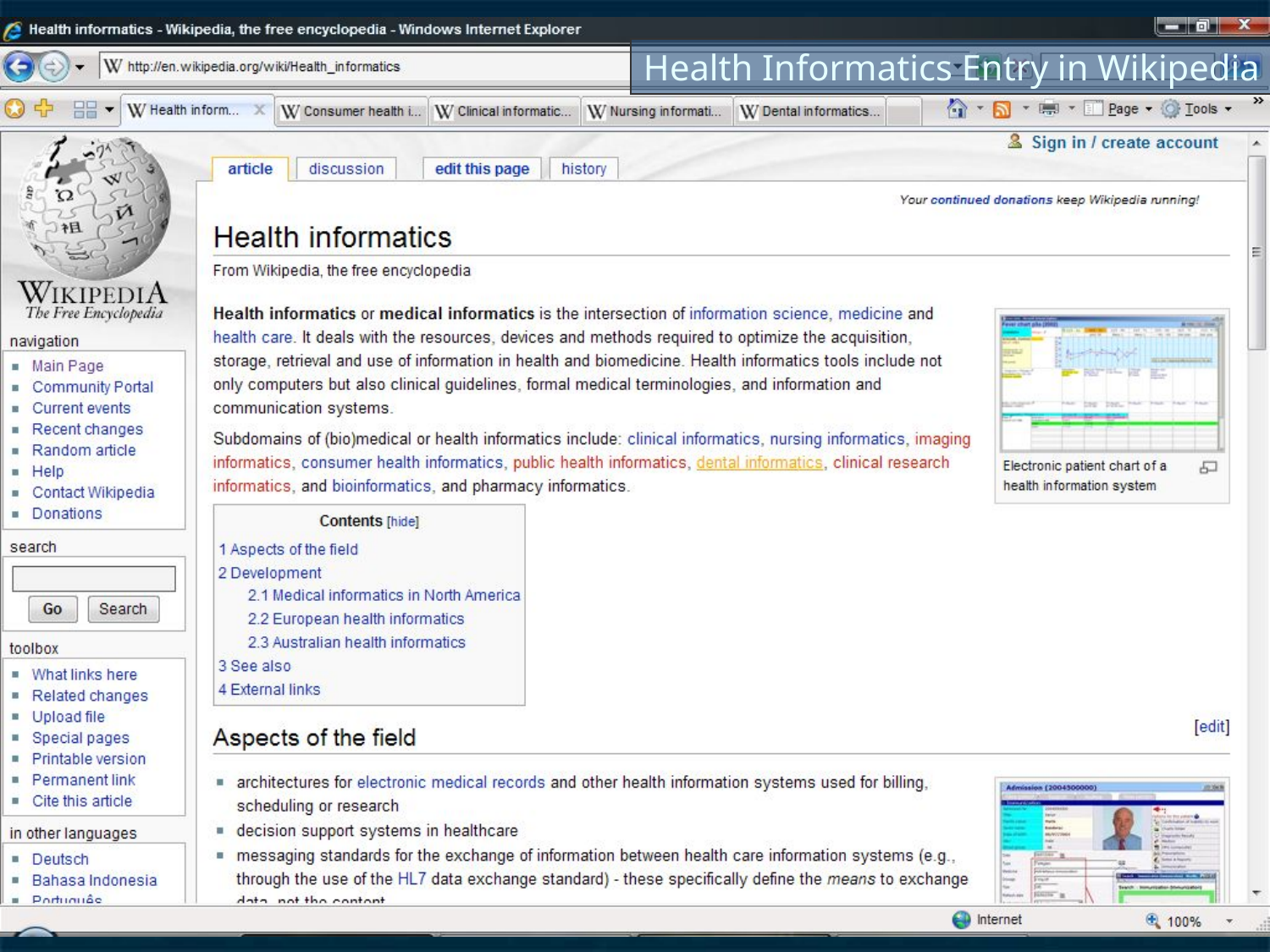

Health Informatics Entry in Wikipedia

## Slide 13
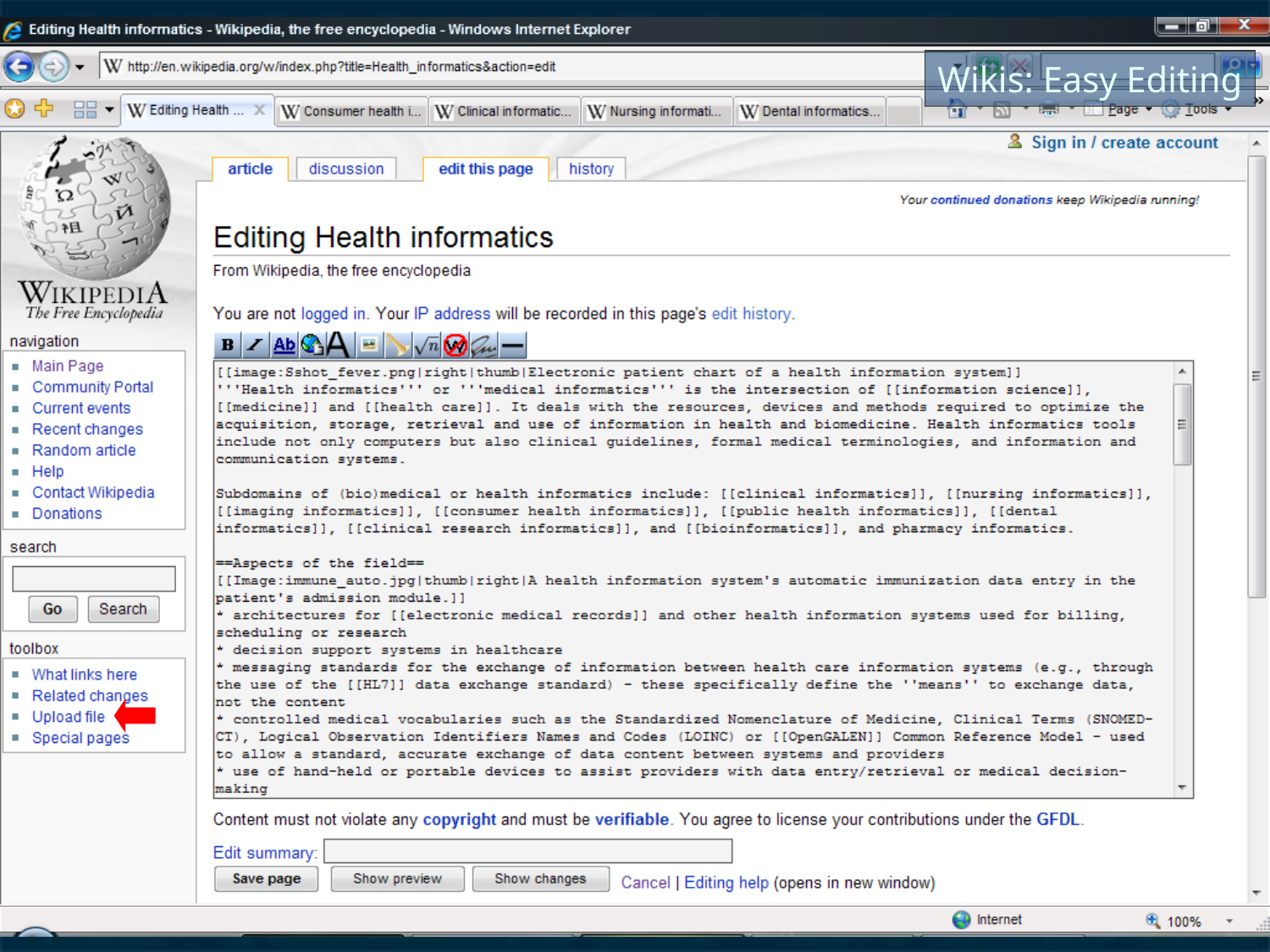

Wikis: Easy Editing

## Slide 14
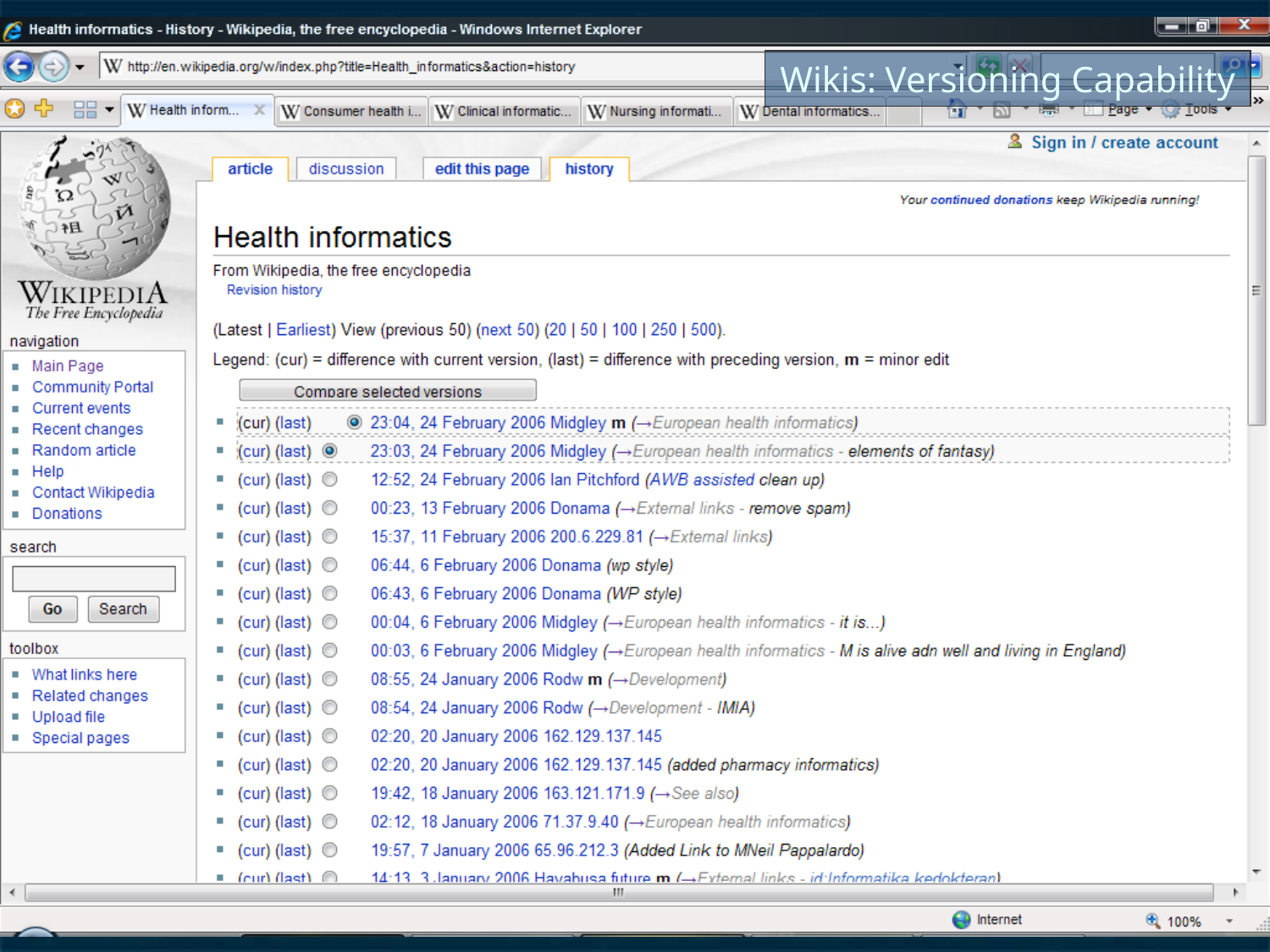

Wikis: Versioning Capability

## Slide 15
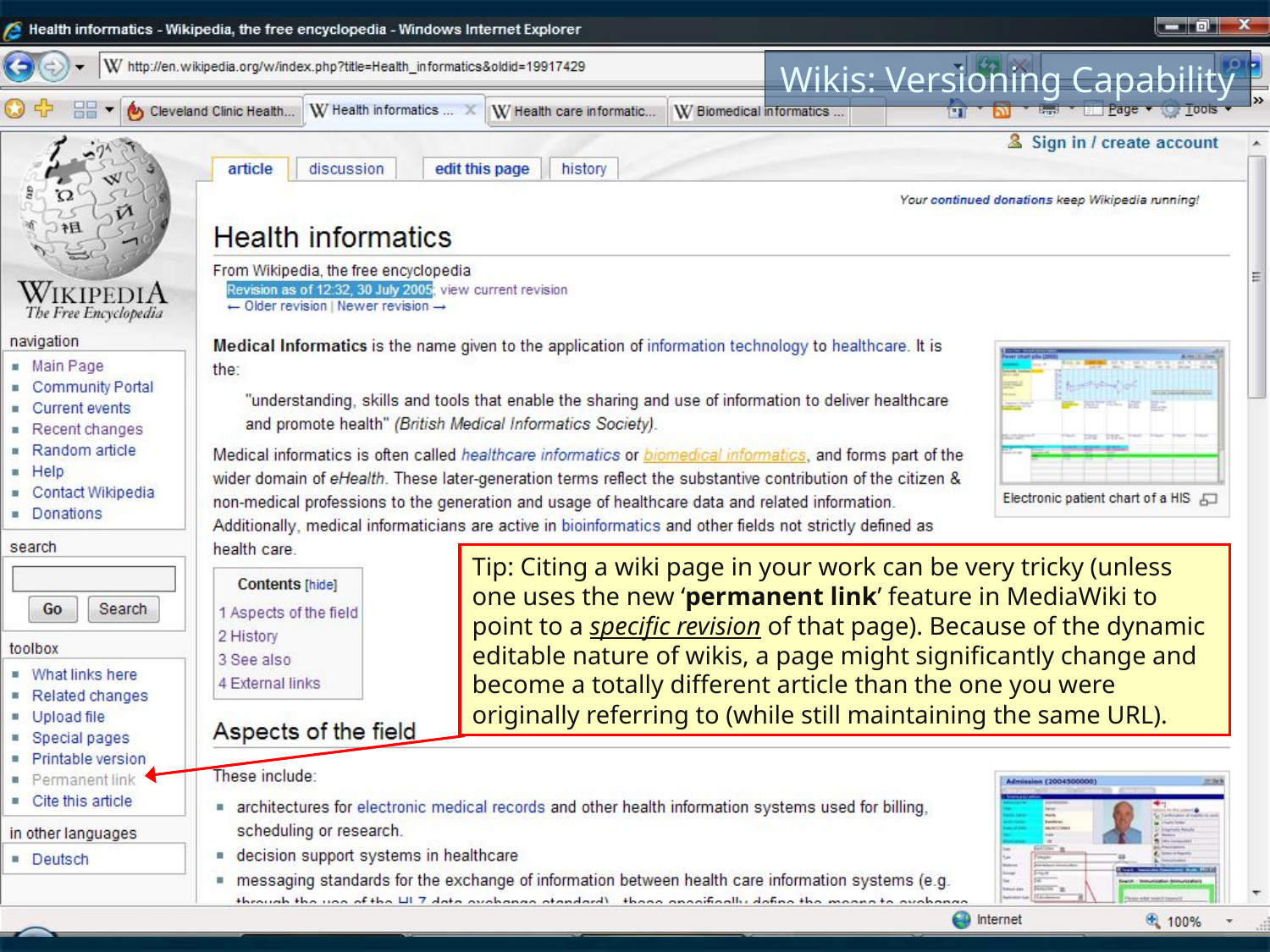

Wikis: Versioning Capability
Tip: Citing a wiki page in your work can be very tricky (unless one uses the new ‘permanent link’ feature in MediaWiki to point to a specific revision of that page). Because of the dynamic editable nature of wikis, a page might significantly change and become a totally different article than the one you were originally referring to (while still maintaining the same URL).

## Slide 16
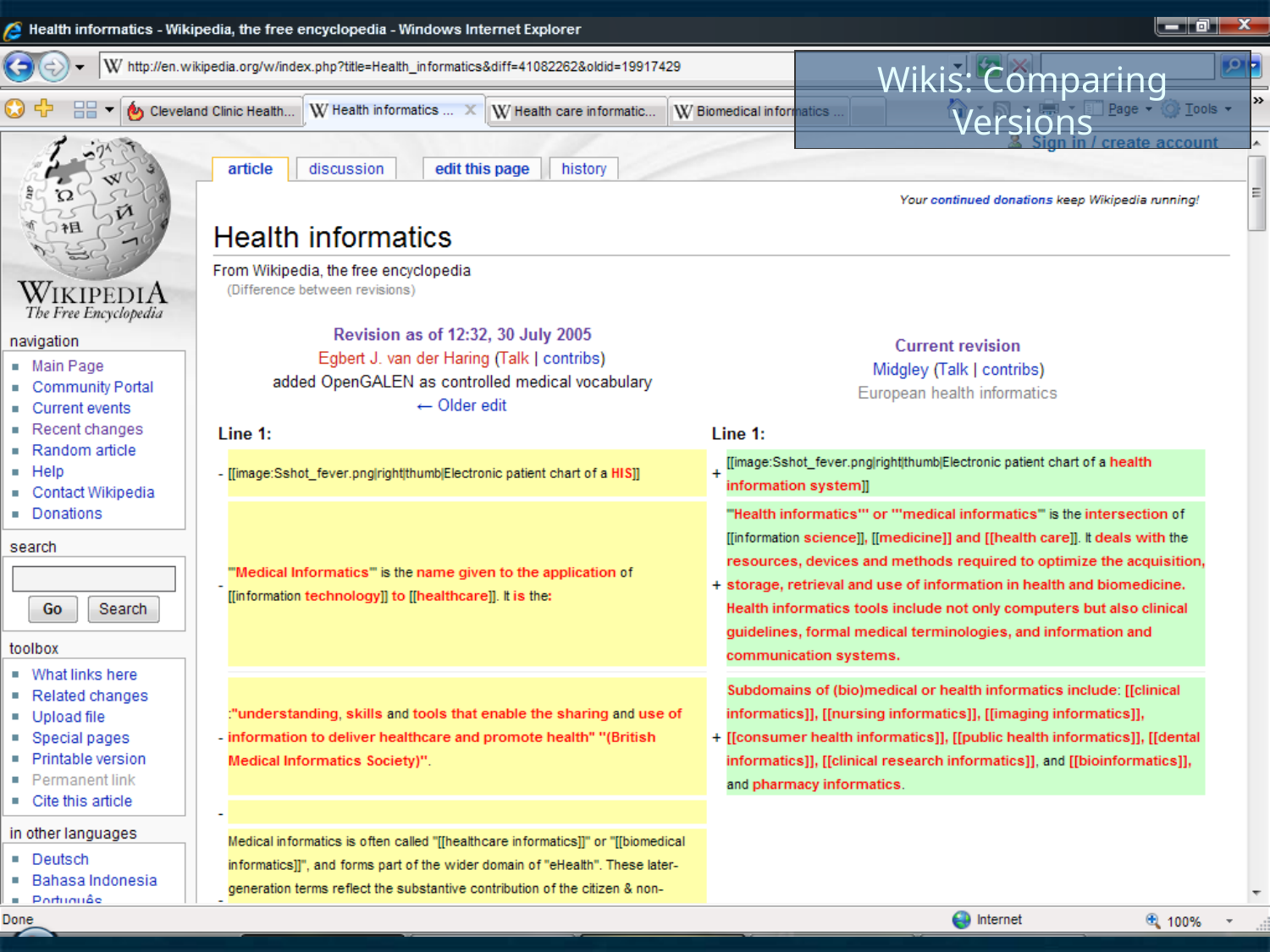

Wikis: Comparing Versions

## Slide 17
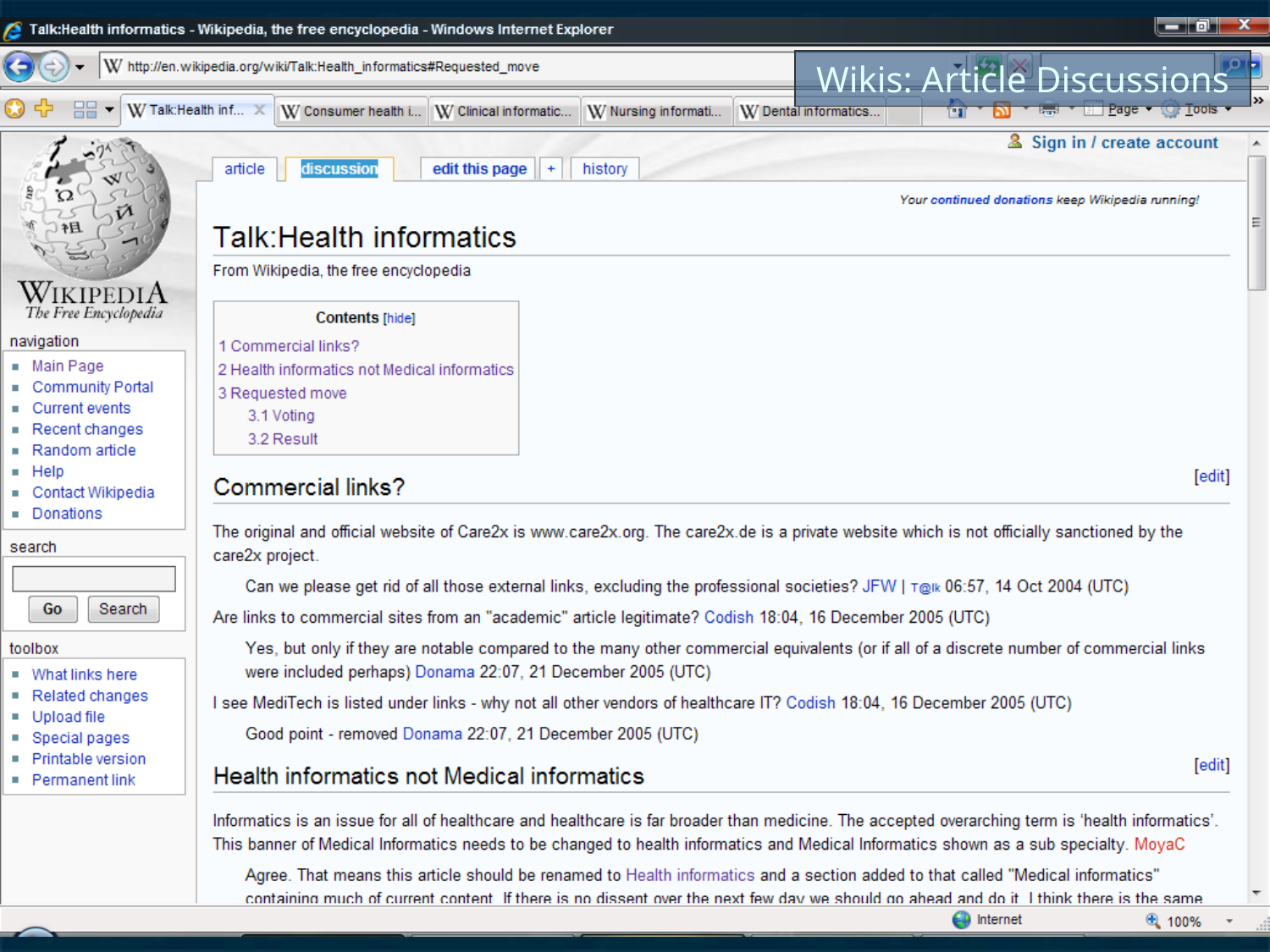

Wikis: Article Discussions

## Slide 18
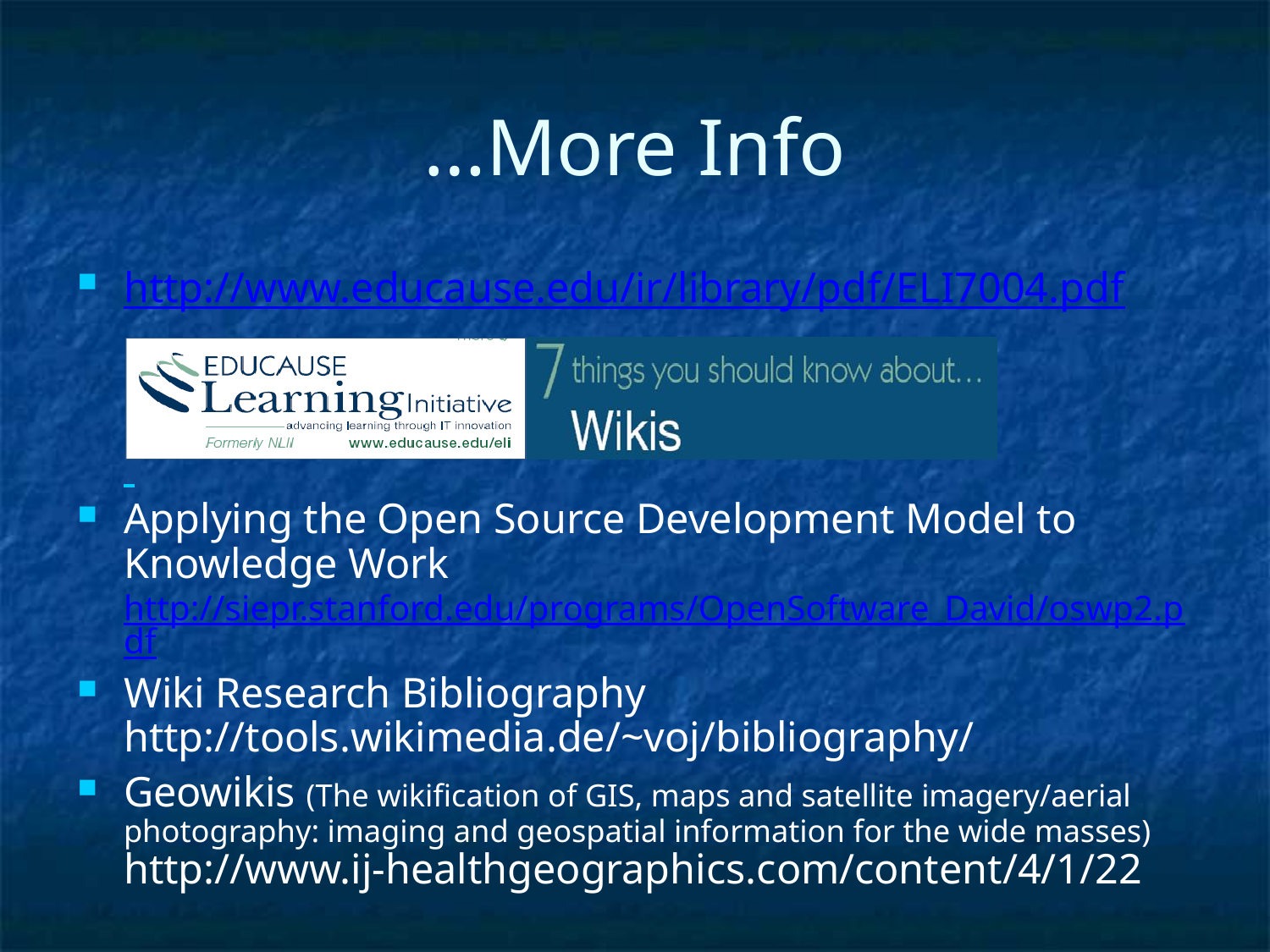

# More Info…
http://www.educause.edu/ir/library/pdf/ELI7004.pdf
Applying the Open Source Development Model to Knowledge Work http://siepr.stanford.edu/programs/OpenSoftware_David/oswp2.pdf
Wiki Research Bibliography http://tools.wikimedia.de/~voj/bibliography/
Geowikis (The wikification of GIS, maps and satellite imagery/aerial photography: imaging and geospatial information for the wide masses)
http://www.ij-healthgeographics.com/content/4/1/22

## Slide 19
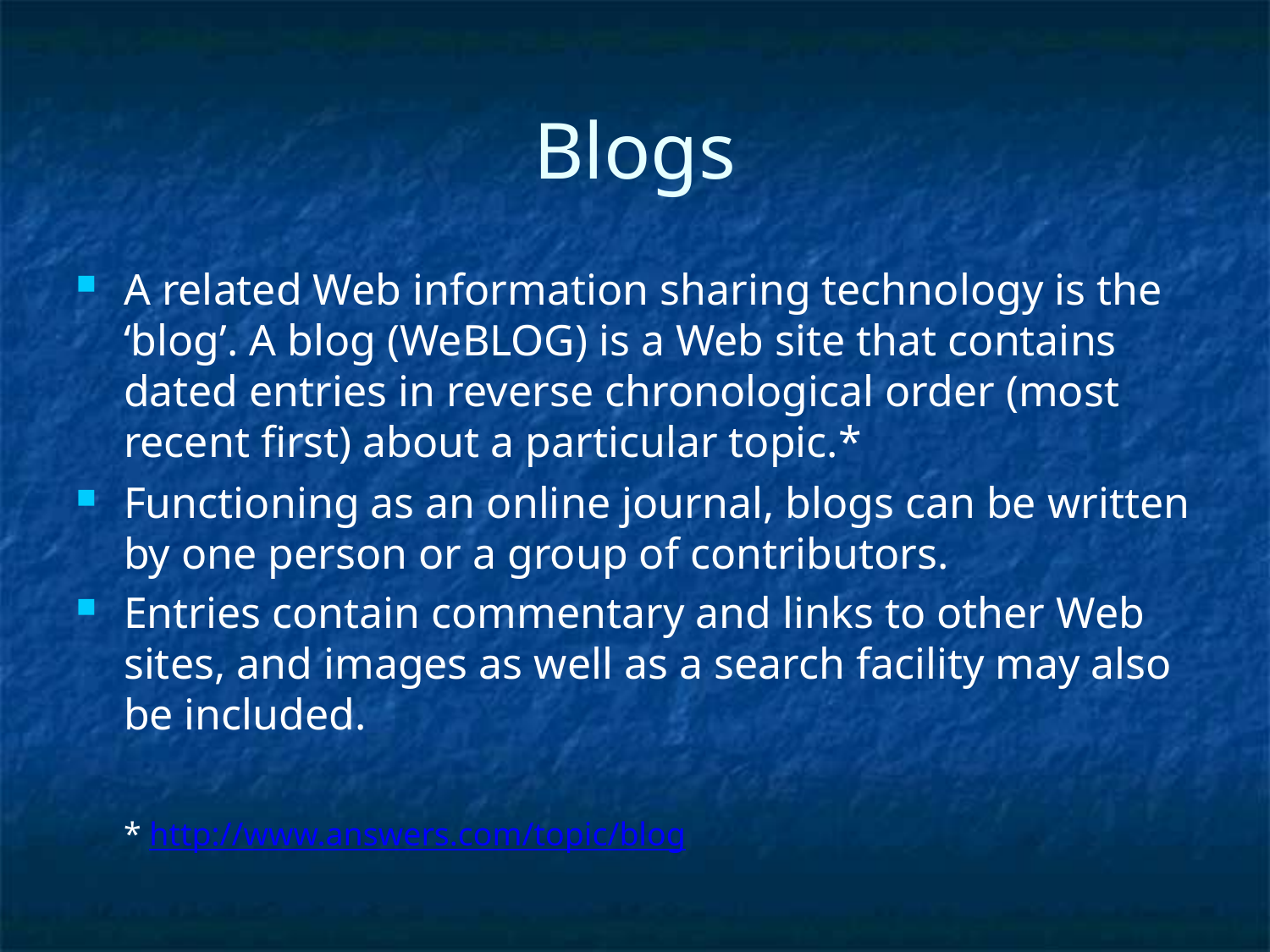

# Blogs
A related Web information sharing technology is the ‘blog’. A blog (WeBLOG) is a Web site that contains dated entries in reverse chronological order (most recent first) about a particular topic.*
Functioning as an online journal, blogs can be written by one person or a group of contributors.
Entries contain commentary and links to other Web sites, and images as well as a search facility may also be included.
* http://www.answers.com/topic/blog

## Slide 20
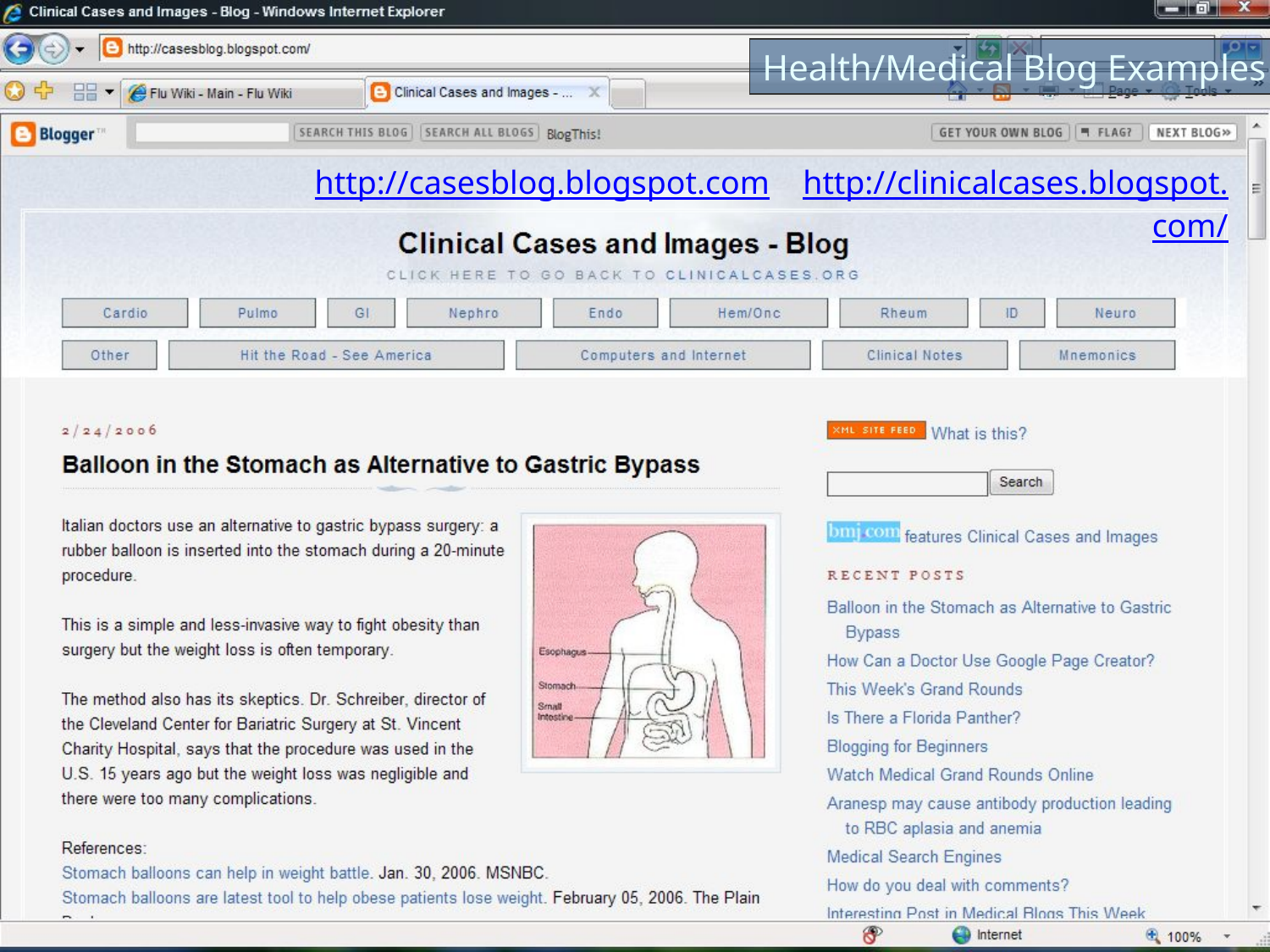

Health/Medical Blog Examples
http://casesblog.blogspot.com http://clinicalcases.blogspot.com/

## Slide 21
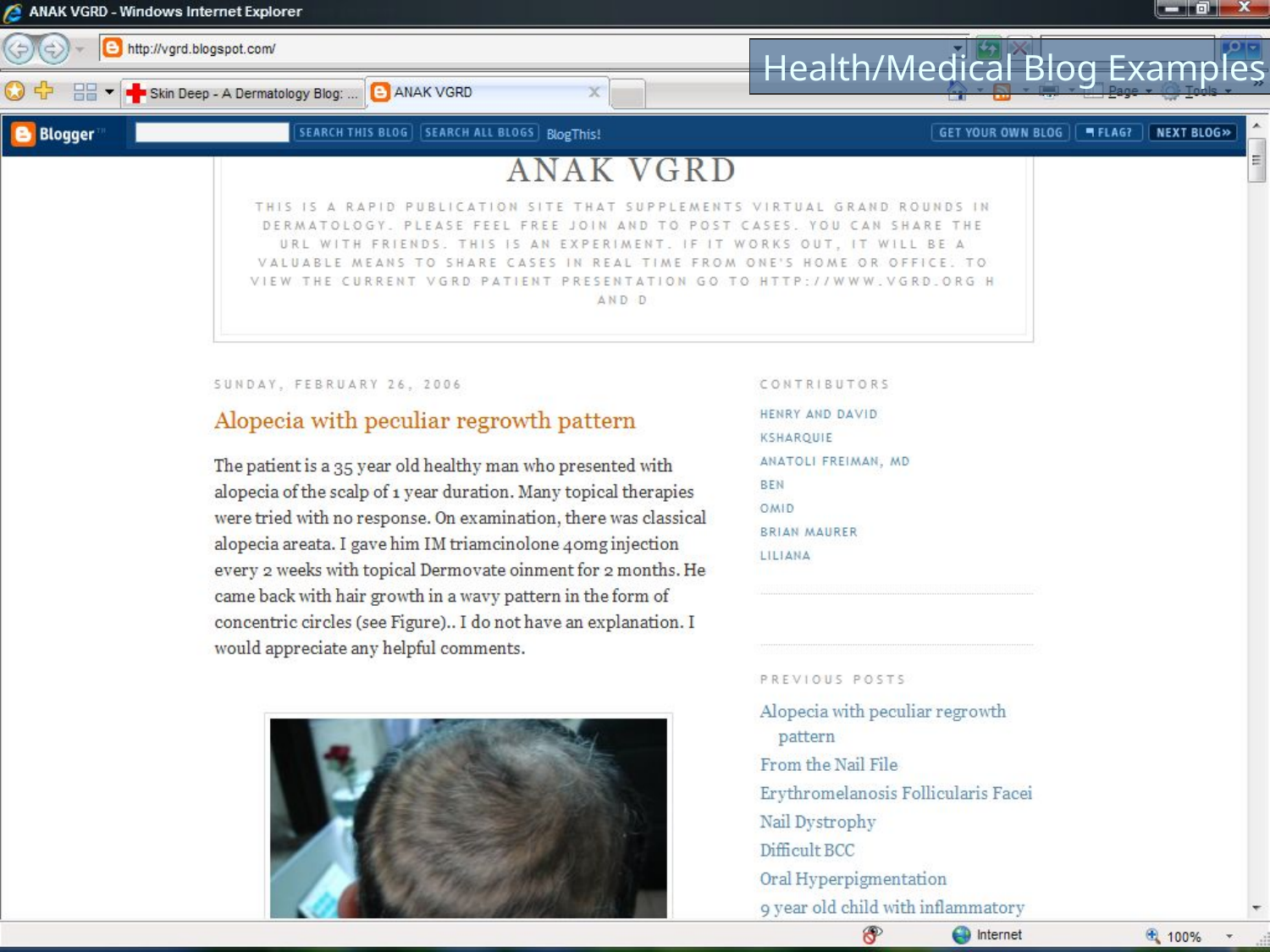

Health/Medical Blog Examples

## Slide 22
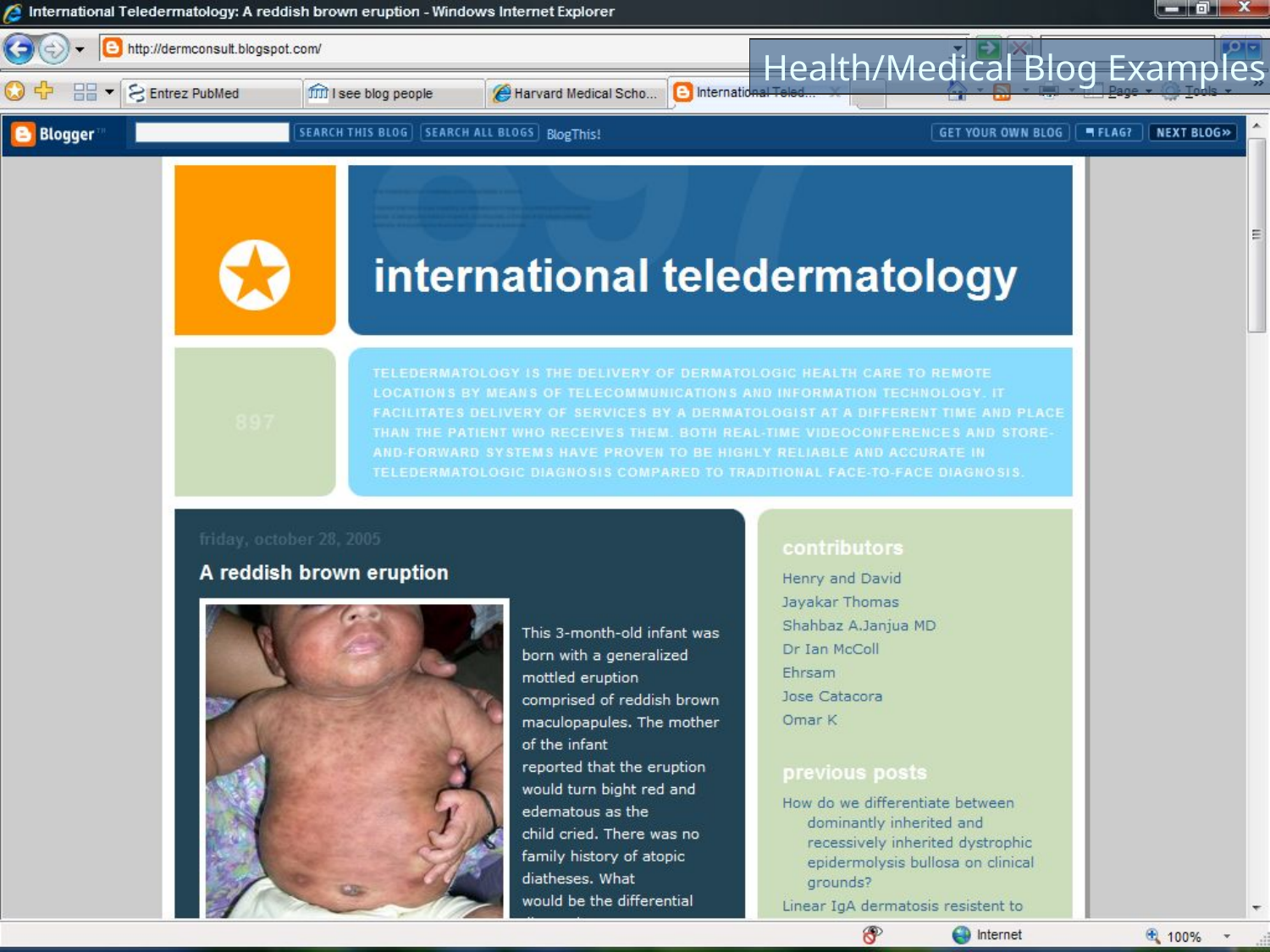

Health/Medical Blog Examples

## Slide 23
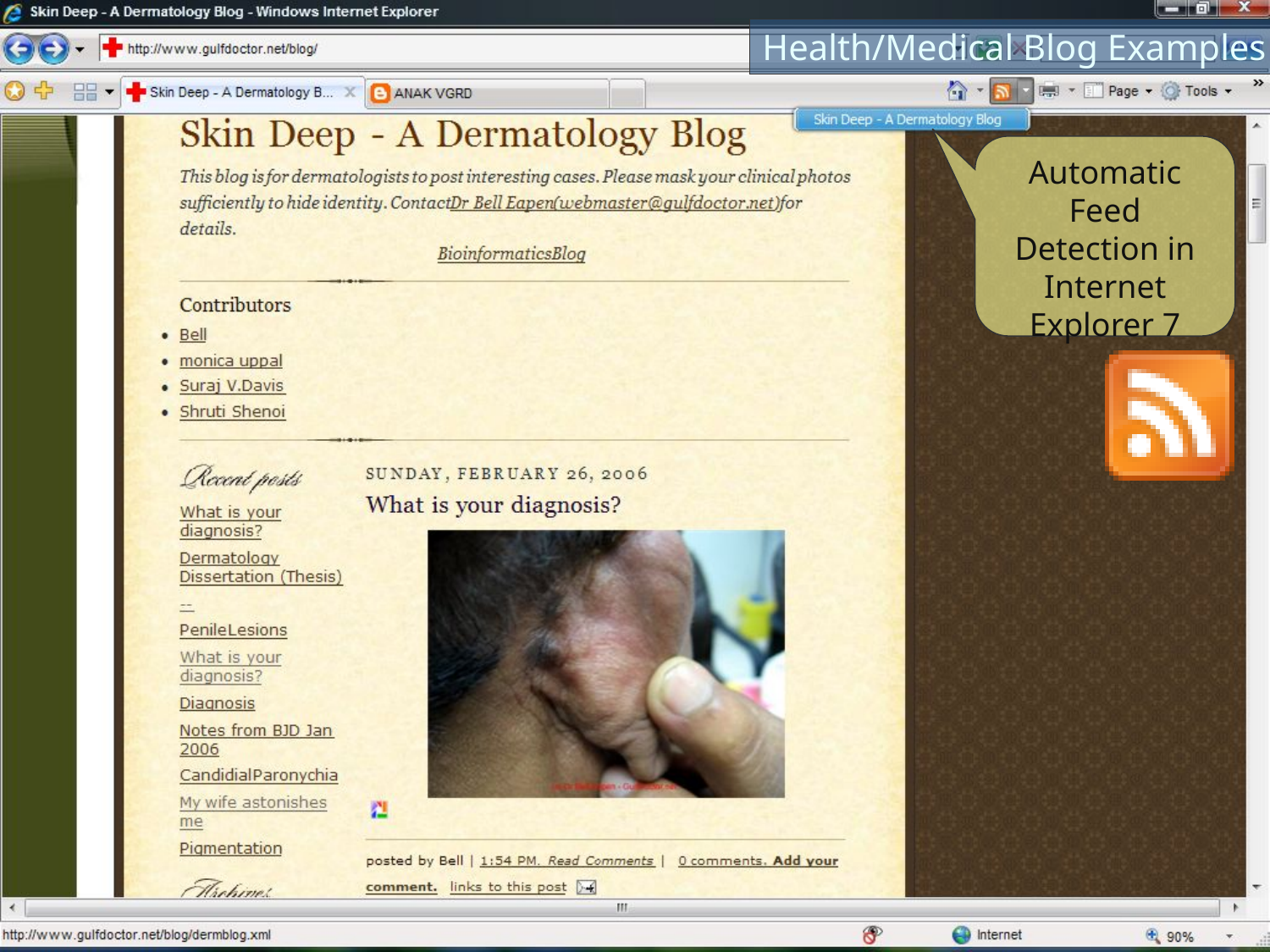

Health/Medical Blog Examples
Automatic Feed Detection in Internet Explorer 7

## Slide 24
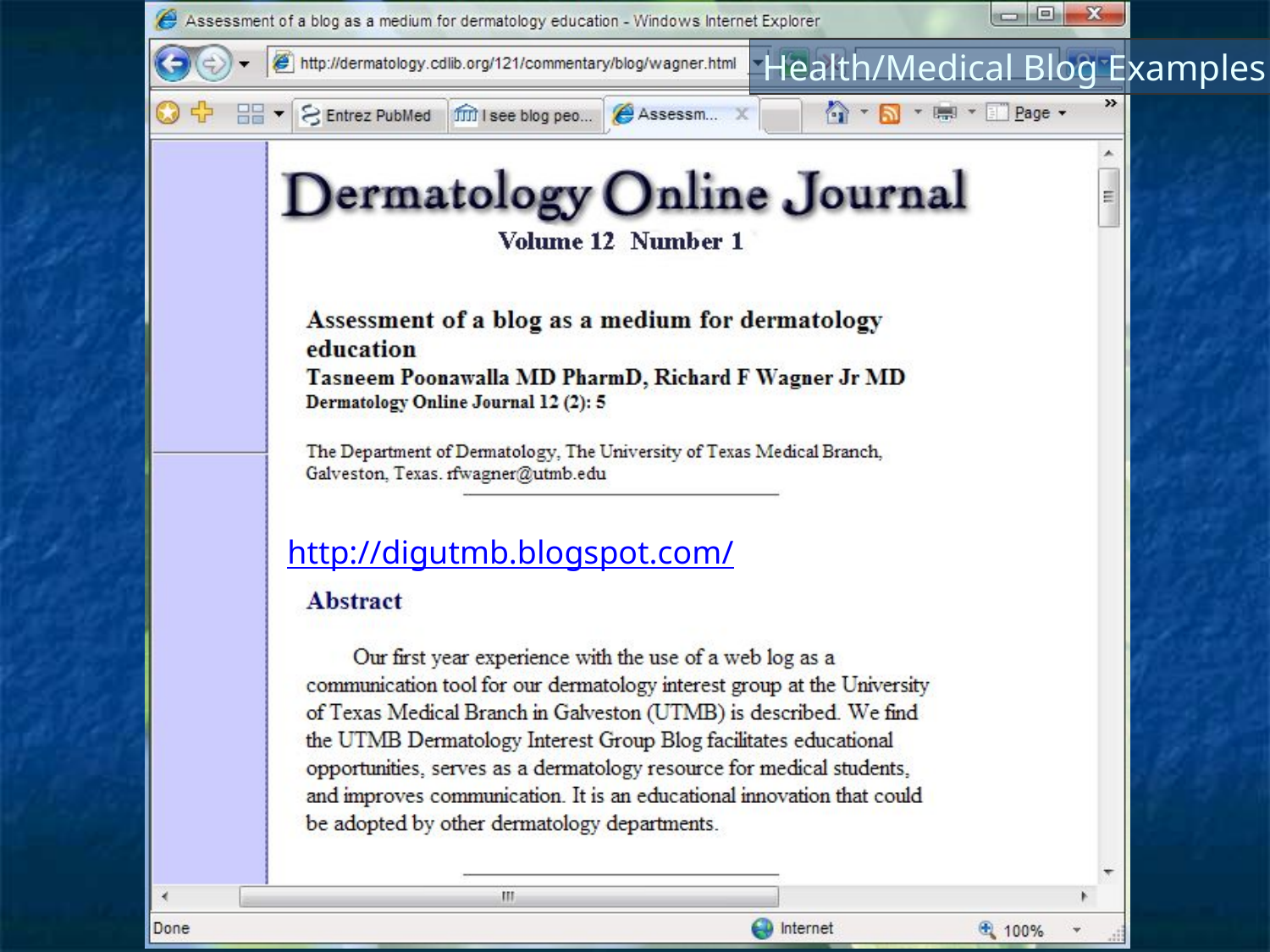

Health/Medical Blog Examples
http://digutmb.blogspot.com/

## Slide 25
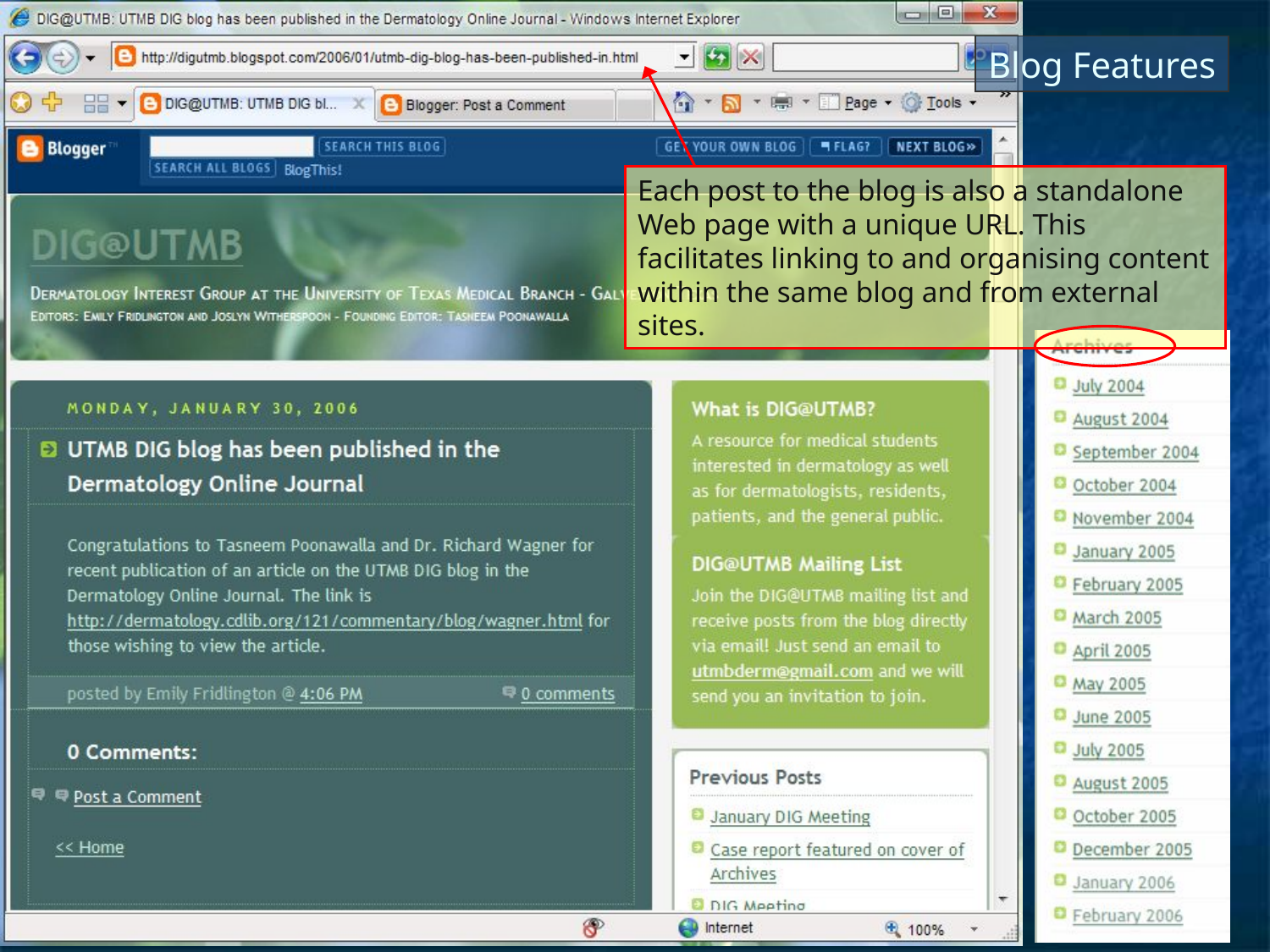

Blog Features
Each post to the blog is also a standalone Web page with a unique URL. This facilitates linking to and organising content within the same blog and from external sites.

## Slide 26
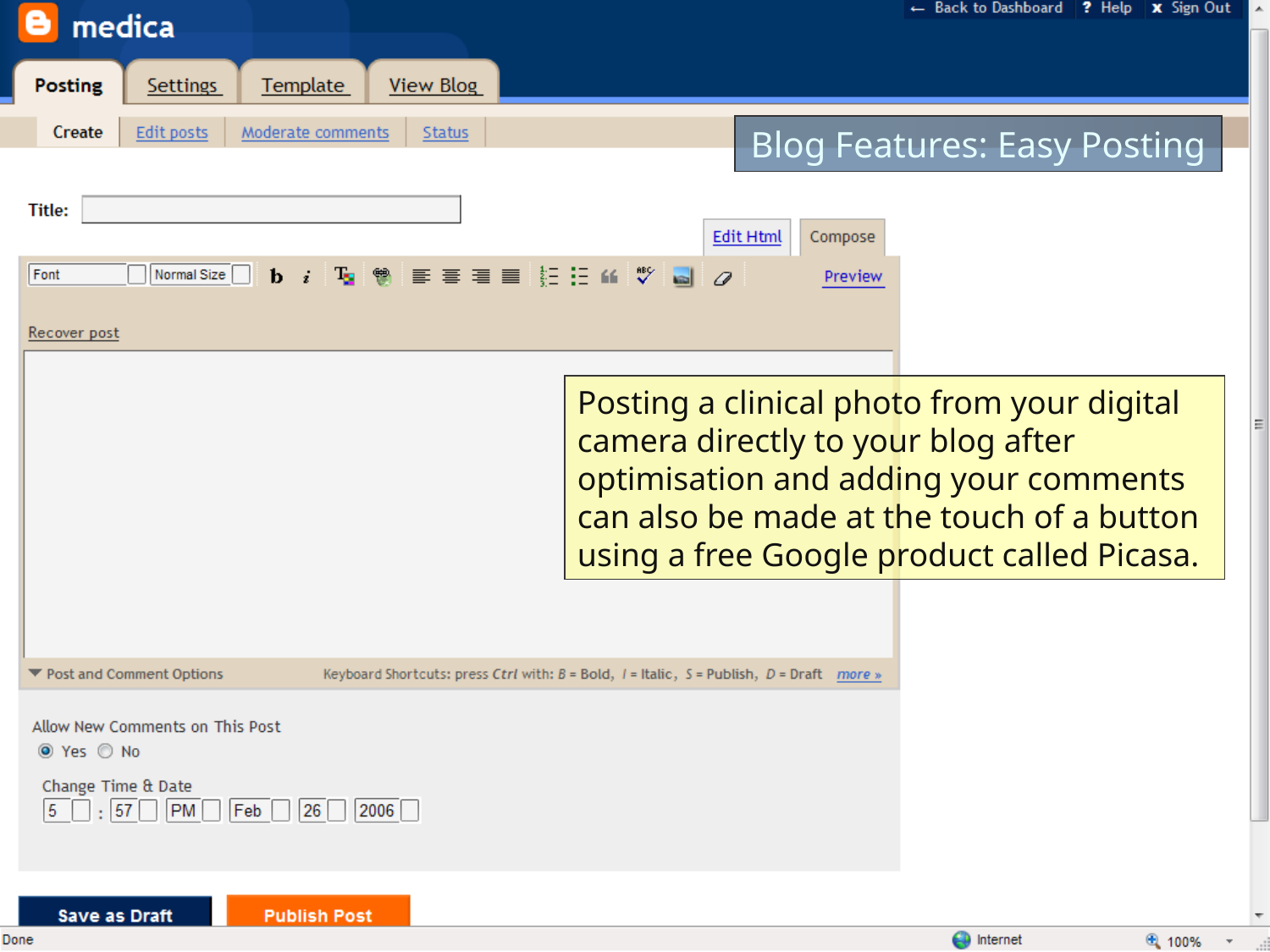

Blog Features: Easy Posting
Posting a clinical photo from your digital camera directly to your blog after optimisation and adding your comments can also be made at the touch of a button using a free Google product called Picasa.

## Slide 27
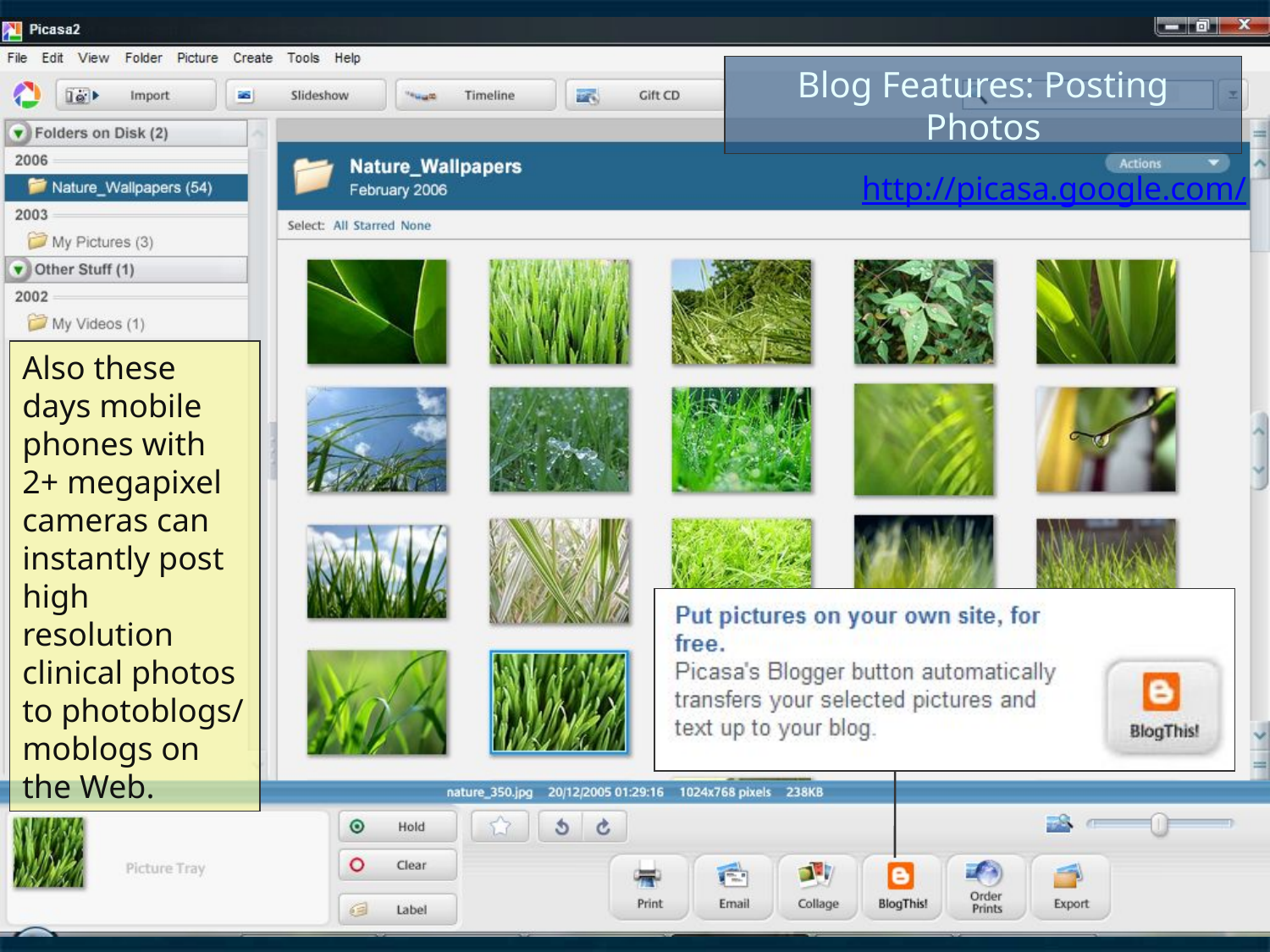

Blog Features: Posting Photos
http://picasa.google.com/
Also these days mobile phones with 2+ megapixel cameras can instantly post high resolution clinical photos to photoblogs/ moblogs on the Web.

## Slide 28
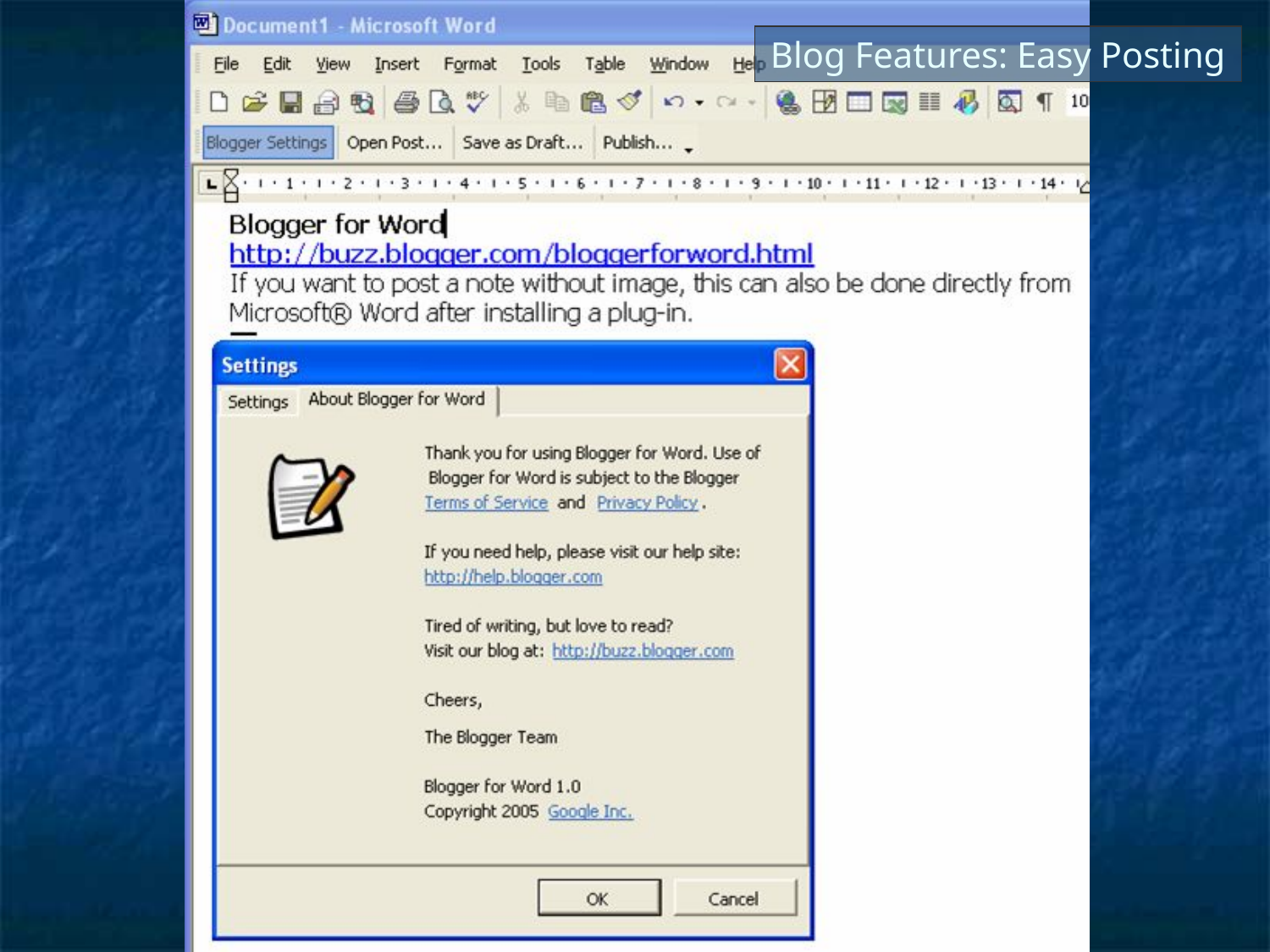

Blog Features: Easy Posting

## Slide 29
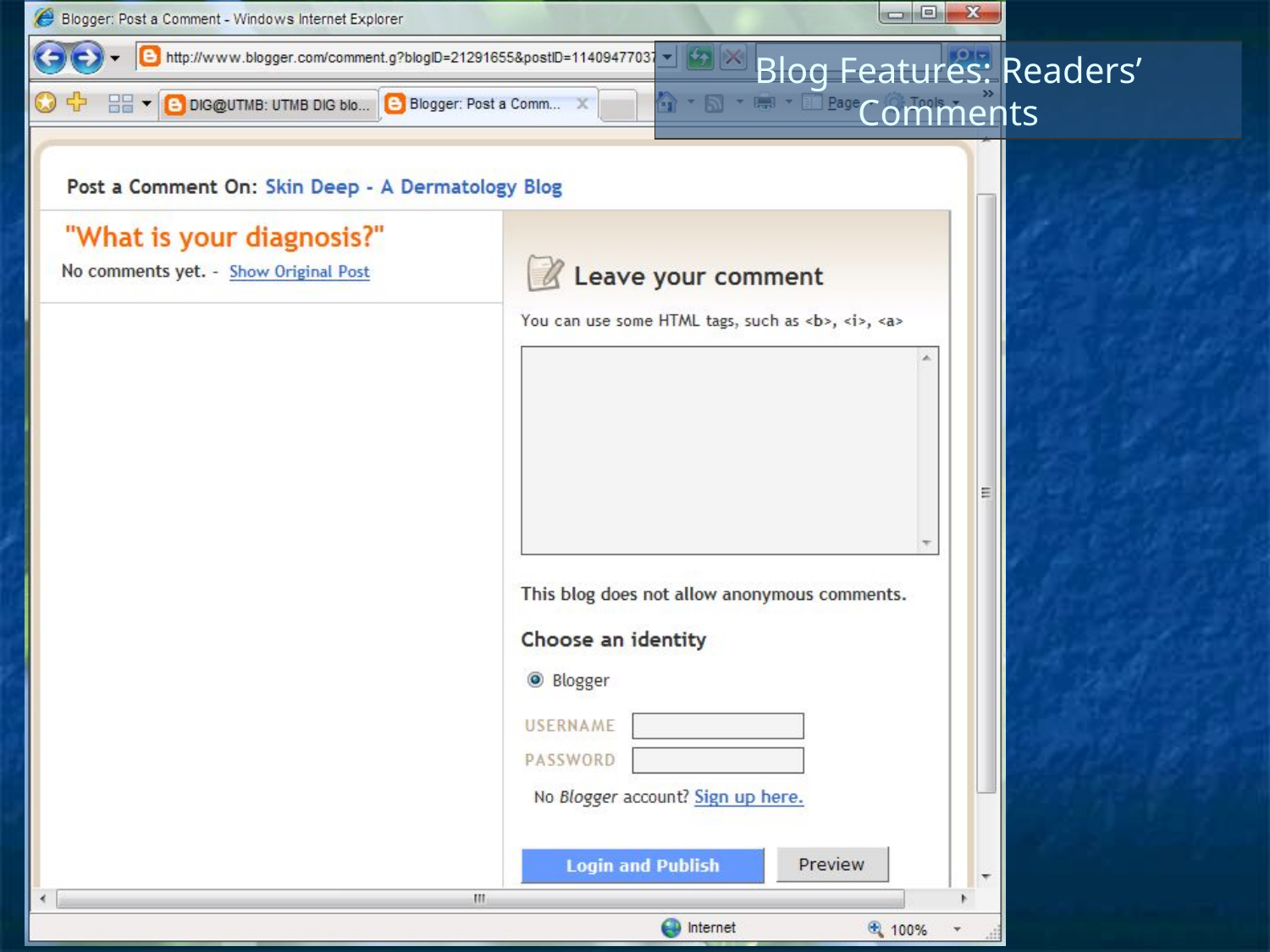

Blog Features: Readers’ Comments

## Slide 30
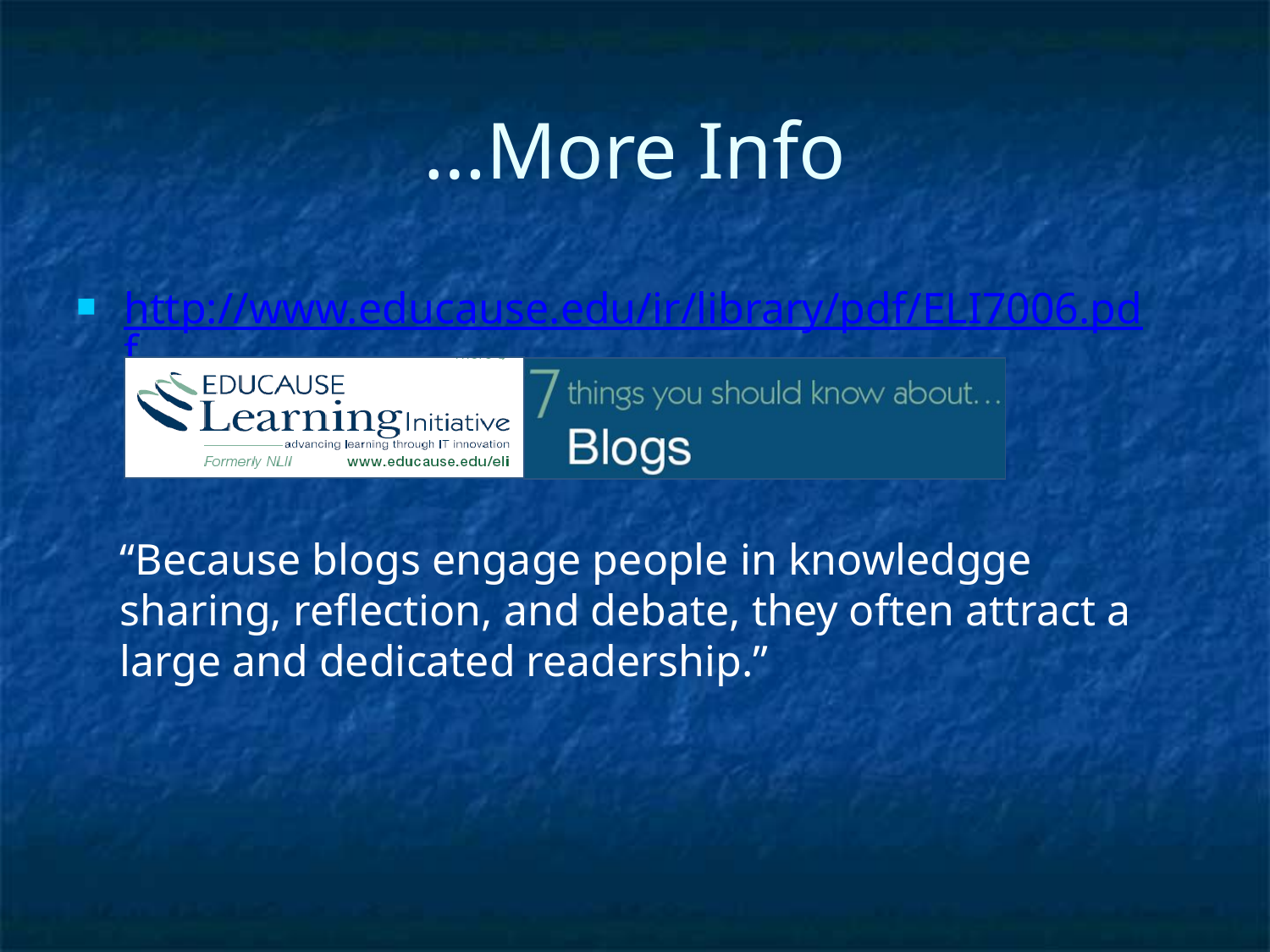

# More Info…
http://www.educause.edu/ir/library/pdf/ELI7006.pdf
“Because blogs engage people in knowledgge sharing, reflection, and debate, they often attract a large and dedicated readership.”

## Slide 31
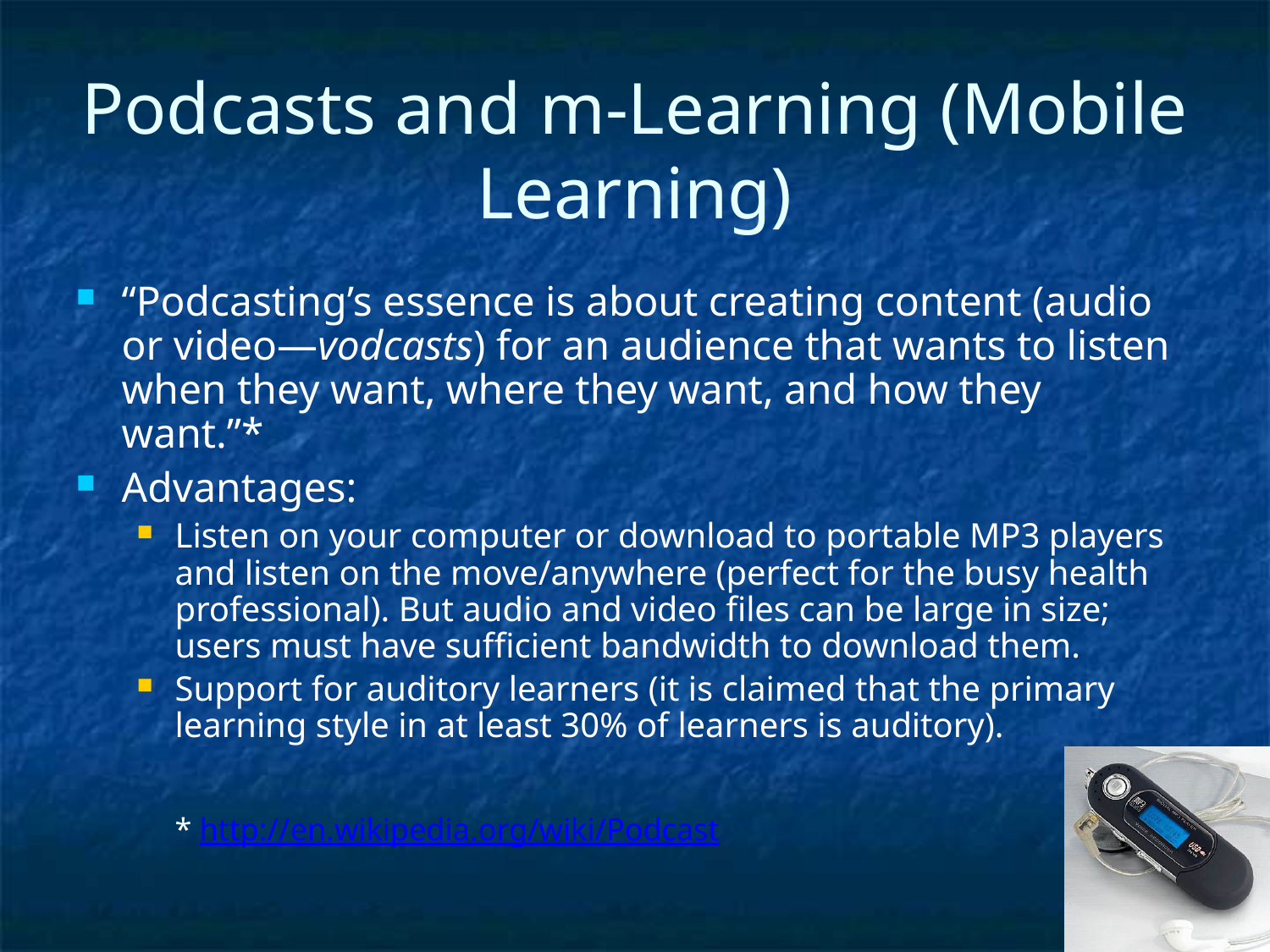

# Podcasts and m-Learning (Mobile Learning)
“Podcasting’s essence is about creating content (audio or video—vodcasts) for an audience that wants to listen when they want, where they want, and how they want.”*
Advantages:
Listen on your computer or download to portable MP3 players and listen on the move/anywhere (perfect for the busy health professional). But audio and video files can be large in size; users must have sufficient bandwidth to download them.
Support for auditory learners (it is claimed that the primary learning style in at least 30% of learners is auditory).
* http://en.wikipedia.org/wiki/Podcast

## Slide 32
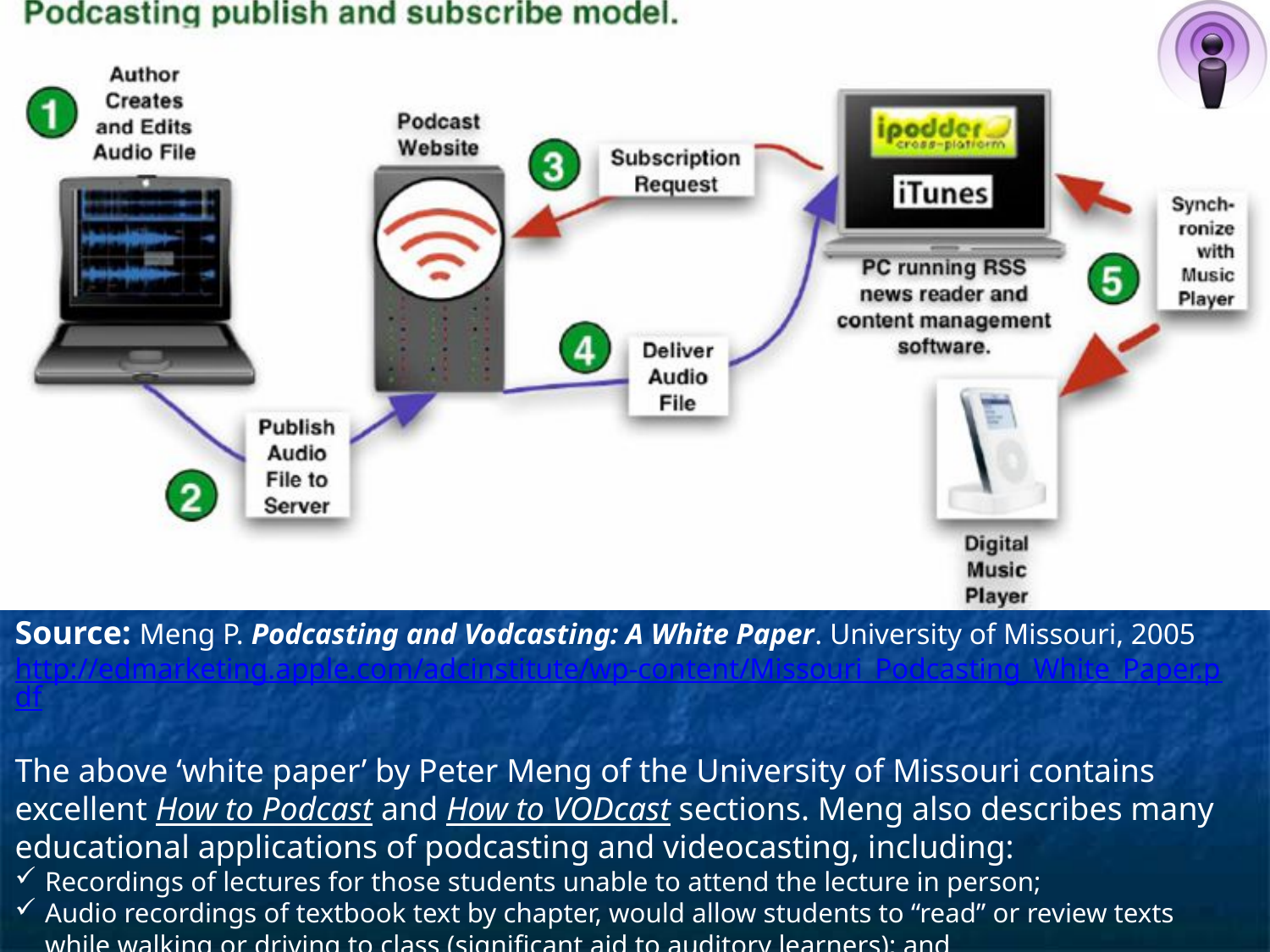

Source: Meng P. Podcasting and Vodcasting: A White Paper. University of Missouri, 2005
http://edmarketing.apple.com/adcinstitute/wp-content/Missouri_Podcasting_White_Paper.pdf
The above ‘white paper’ by Peter Meng of the University of Missouri contains excellent How to Podcast and How to VODcast sections. Meng also describes many educational applications of podcasting and videocasting, including:
Recordings of lectures for those students unable to attend the lecture in person;
Audio recordings of textbook text by chapter, would allow students to “read” or review texts while walking or driving to class (significant aid to auditory learners); and
Downloadable libraries of high resolution heart and respiratory sounds for medical students.

## Slide 33
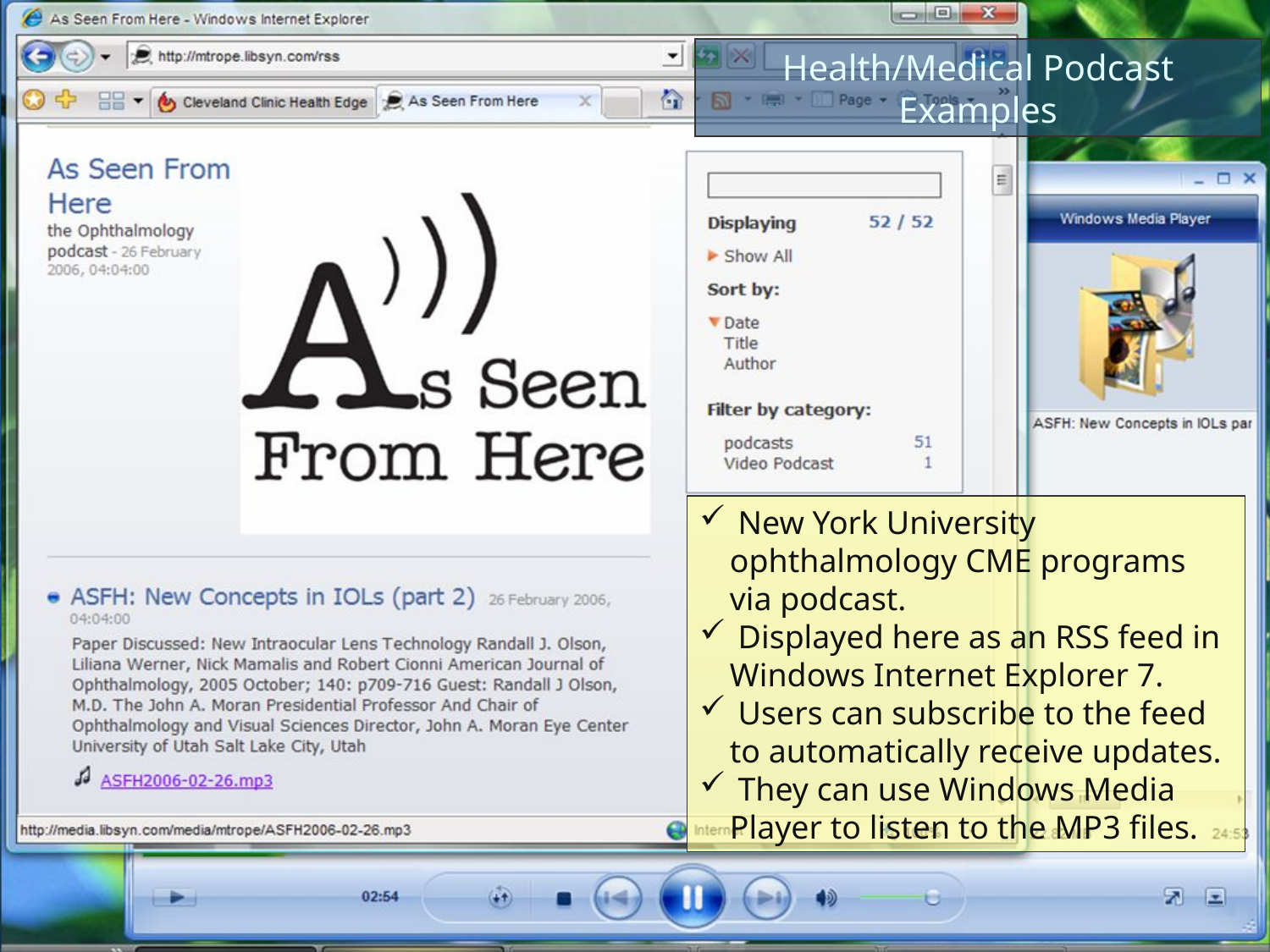

Health/Medical Podcast Examples
 New York University ophthalmology CME programs via podcast.
 Displayed here as an RSS feed in Windows Internet Explorer 7.
 Users can subscribe to the feed to automatically receive updates.
 They can use Windows Media Player to listen to the MP3 files.

## Slide 34
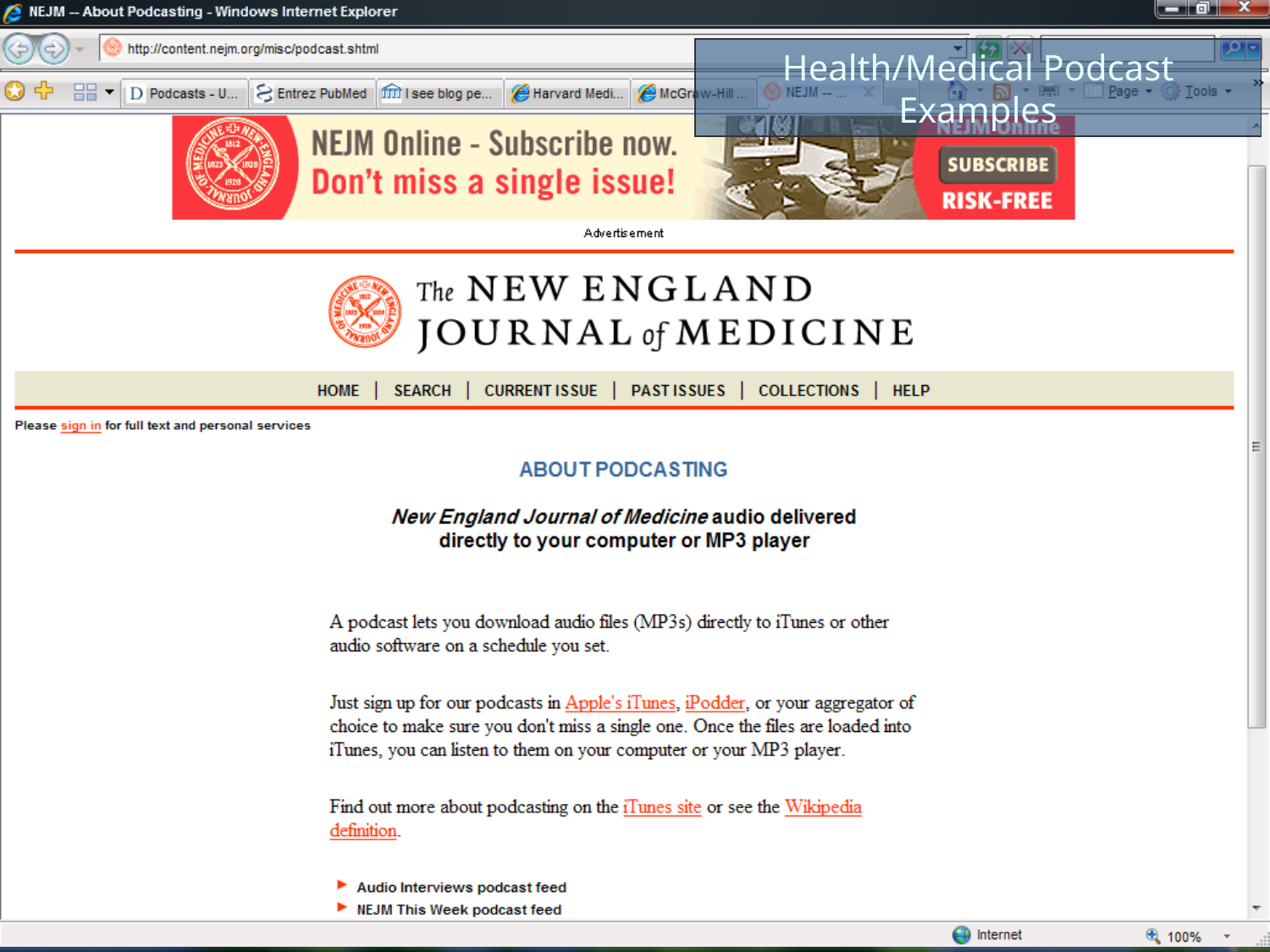

Health/Medical Podcast Examples

## Slide 35
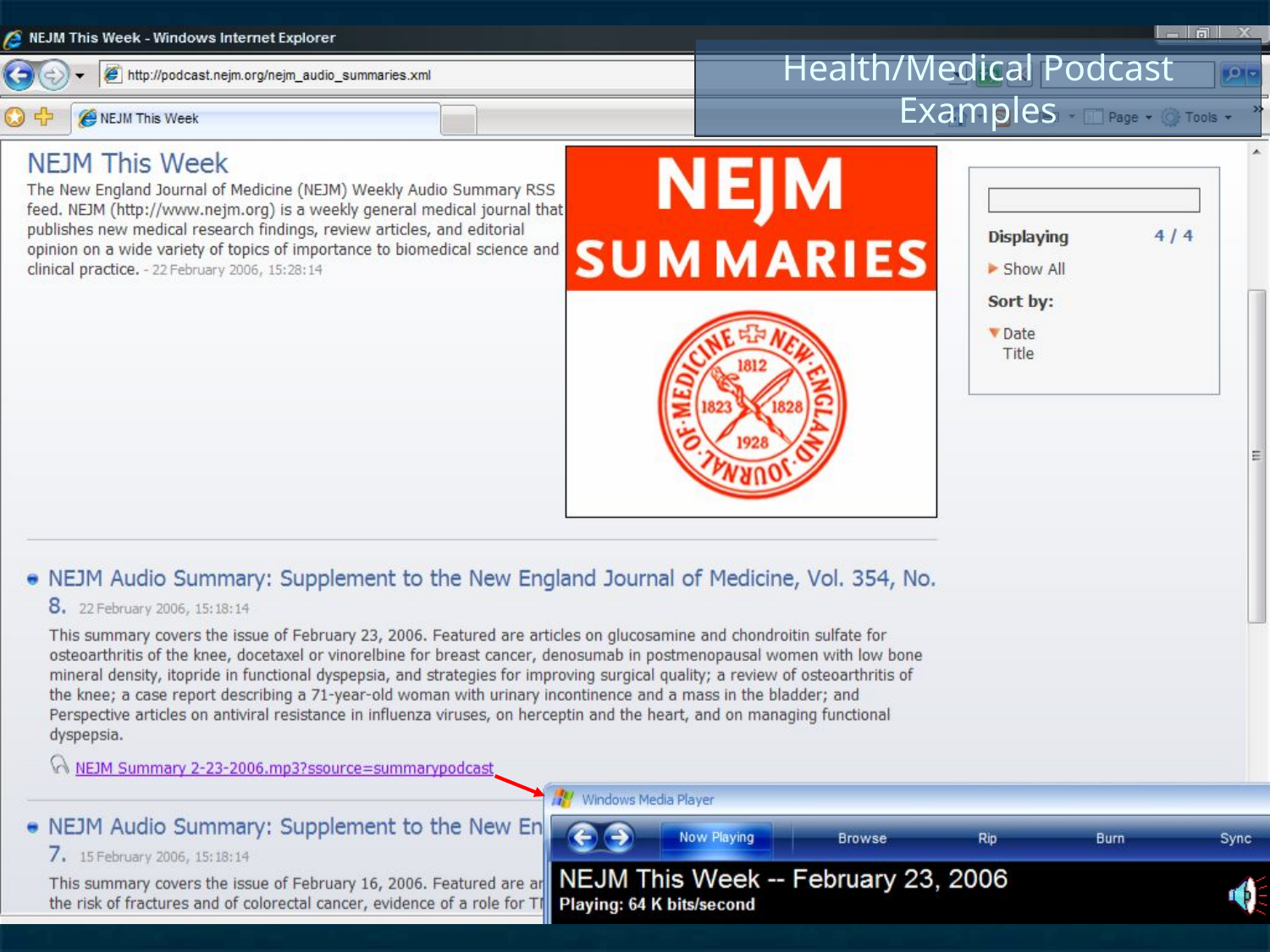

Health/Medical Podcast Examples

## Slide 36
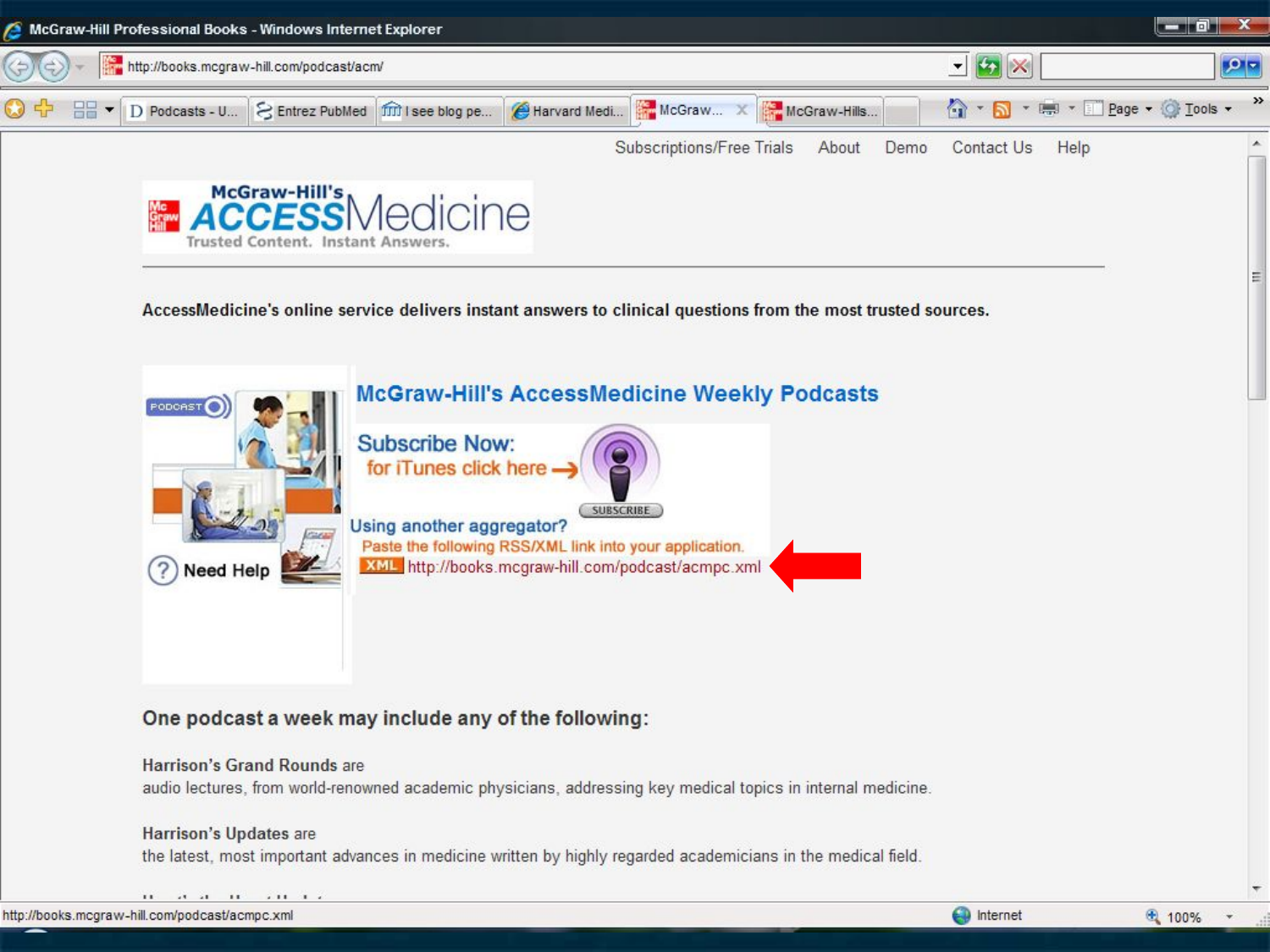

## Slide 37
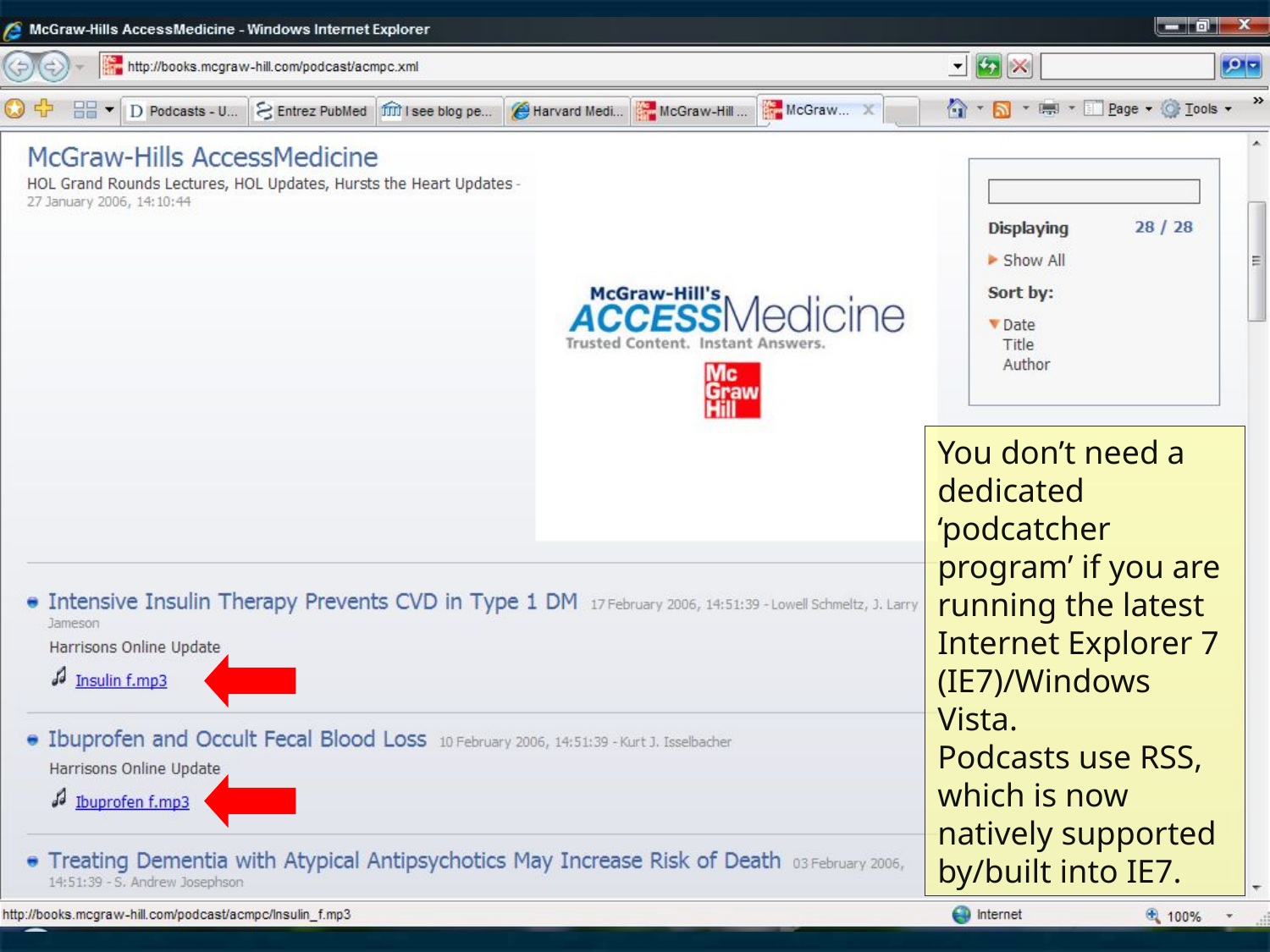

You don’t need a dedicated ‘podcatcher program’ if you are running the latest Internet Explorer 7 (IE7)/Windows Vista.
Podcasts use RSS, which is now natively supported by/built into IE7.

## Slide 38
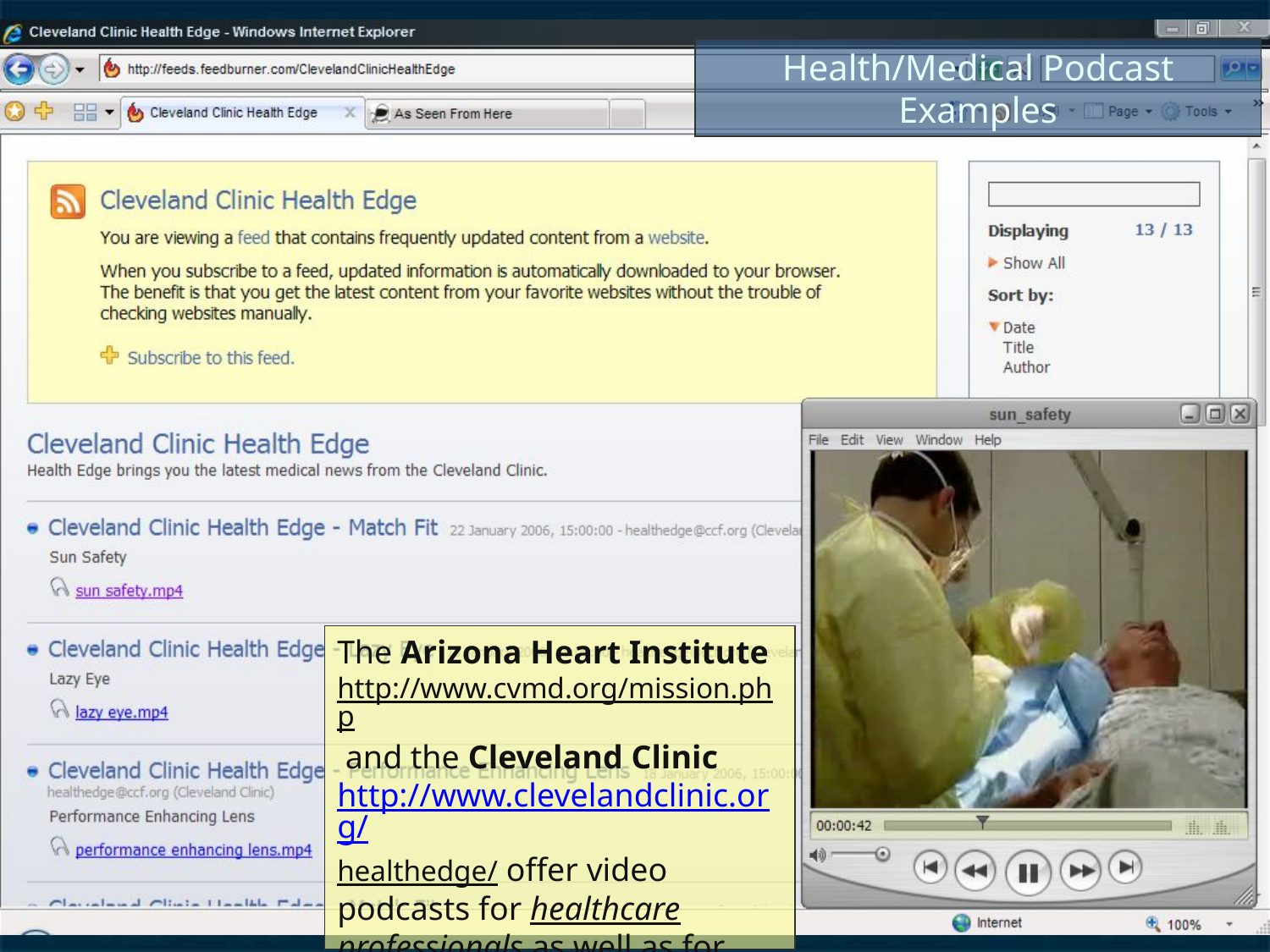

Health/Medical Podcast Examples
The Arizona Heart Institute http://www.cvmd.org/mission.php and the Cleveland Clinic http://www.clevelandclinic.org/healthedge/ offer video podcasts for healthcare professionals as well as for patients.

## Slide 39
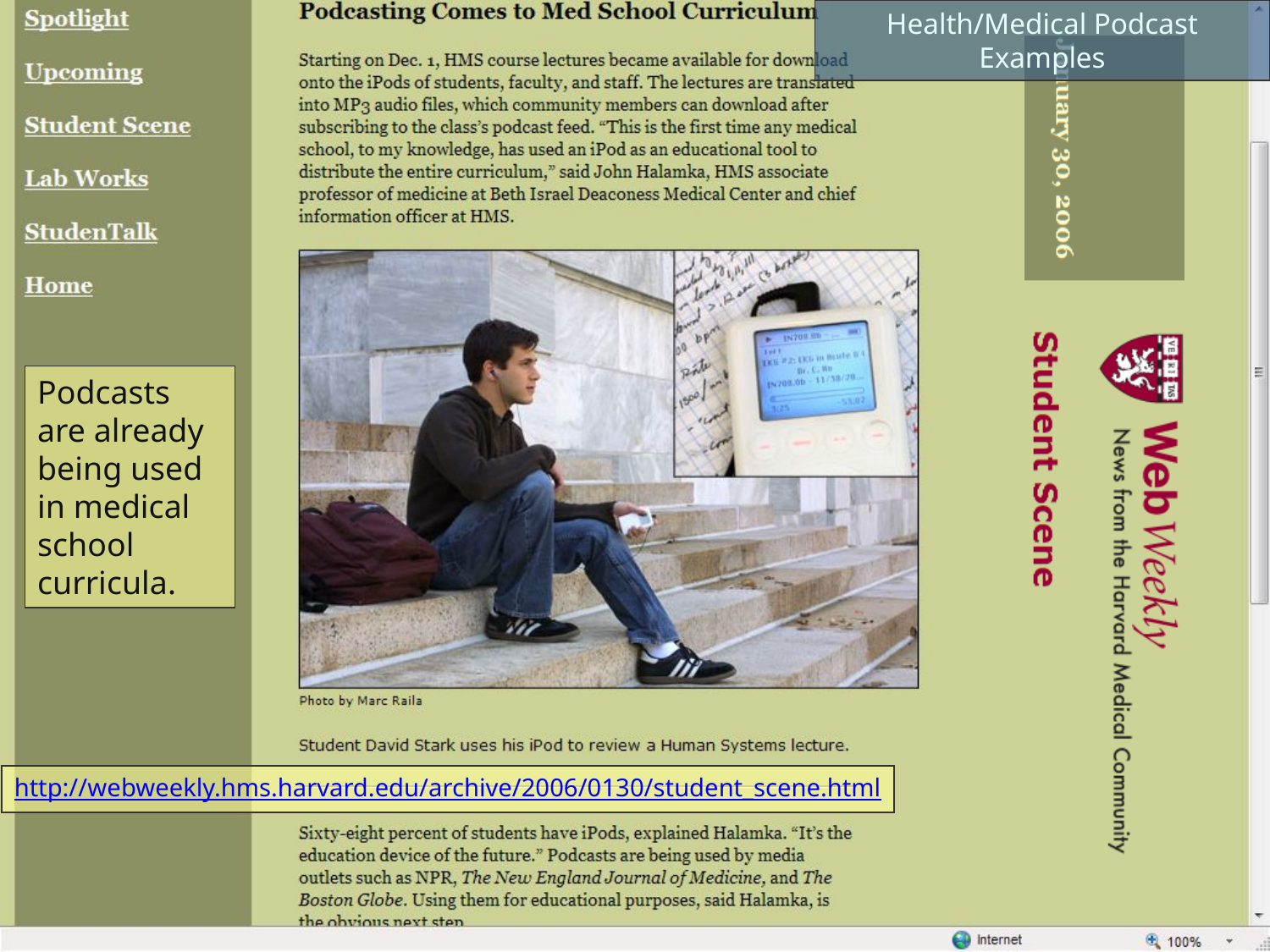

Health/Medical Podcast Examples
Podcasts are already being used in medical school curricula.
http://webweekly.hms.harvard.edu/archive/2006/0130/student_scene.html

## Slide 40
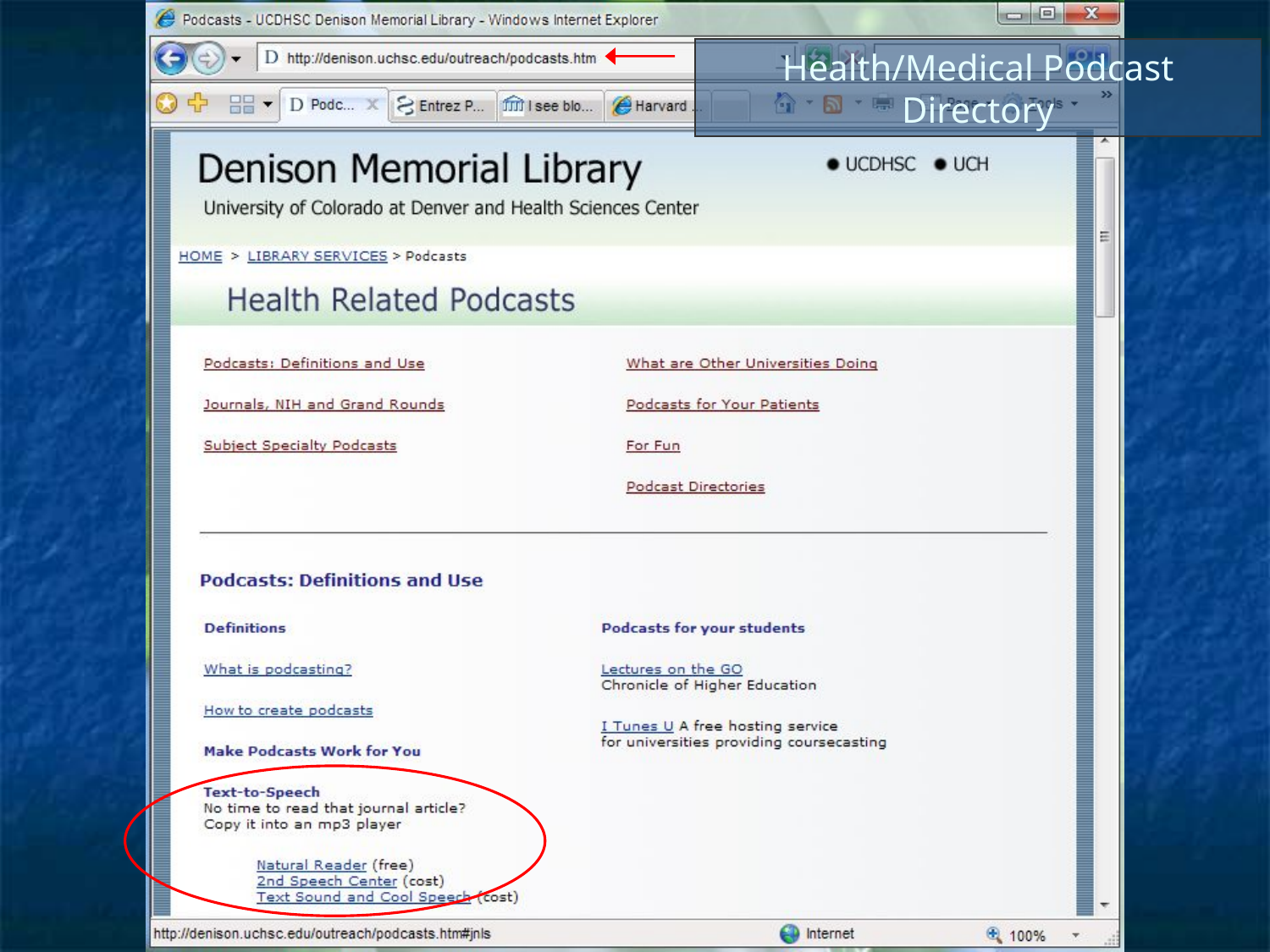

Health/Medical Podcast Directory

## Slide 41
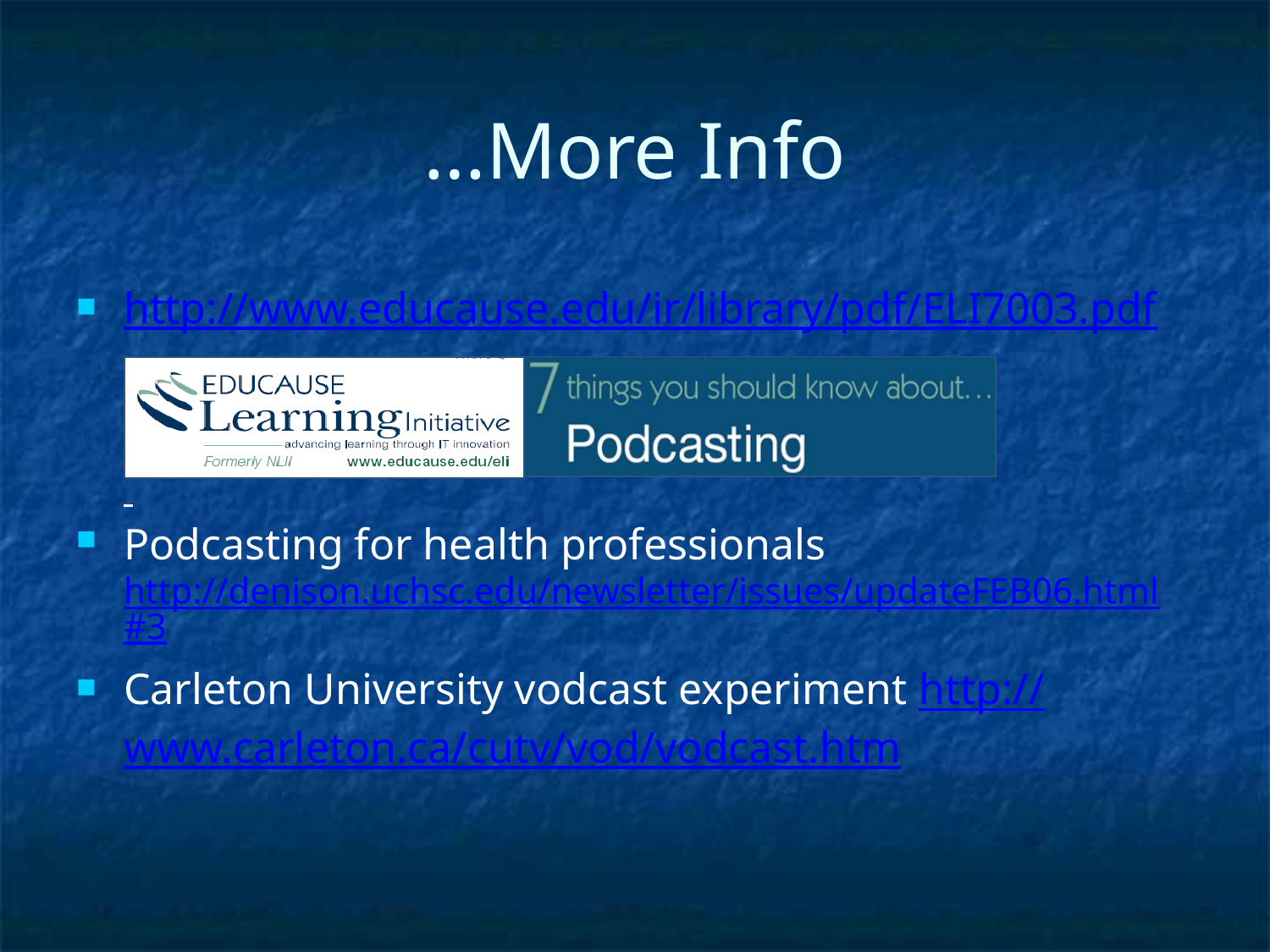

# More Info…
http://www.educause.edu/ir/library/pdf/ELI7003.pdf
Podcasting for health professionals http://denison.uchsc.edu/newsletter/issues/updateFEB06.html#3
Carleton University vodcast experiment http://www.carleton.ca/cutv/vod/vodcast.htm

## Slide 42
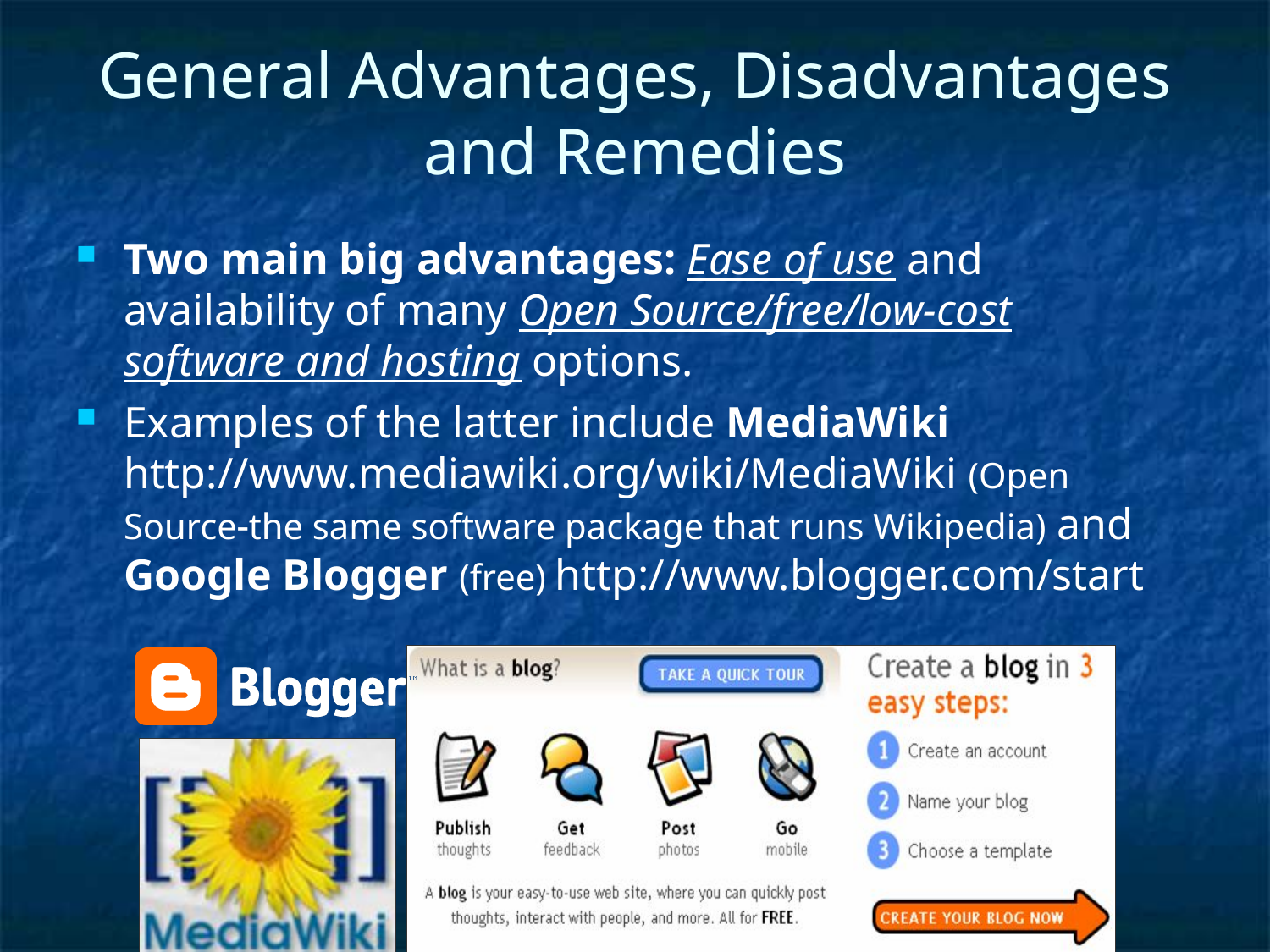

# General Advantages, Disadvantages and Remedies
Two main big advantages: Ease of use and availability of many Open Source/free/low-cost software and hosting options.
Examples of the latter include MediaWiki http://www.mediawiki.org/wiki/MediaWiki (Open Source−the same software package that runs Wikipedia) and Google Blogger (free) http://www.blogger.com/start

## Slide 43
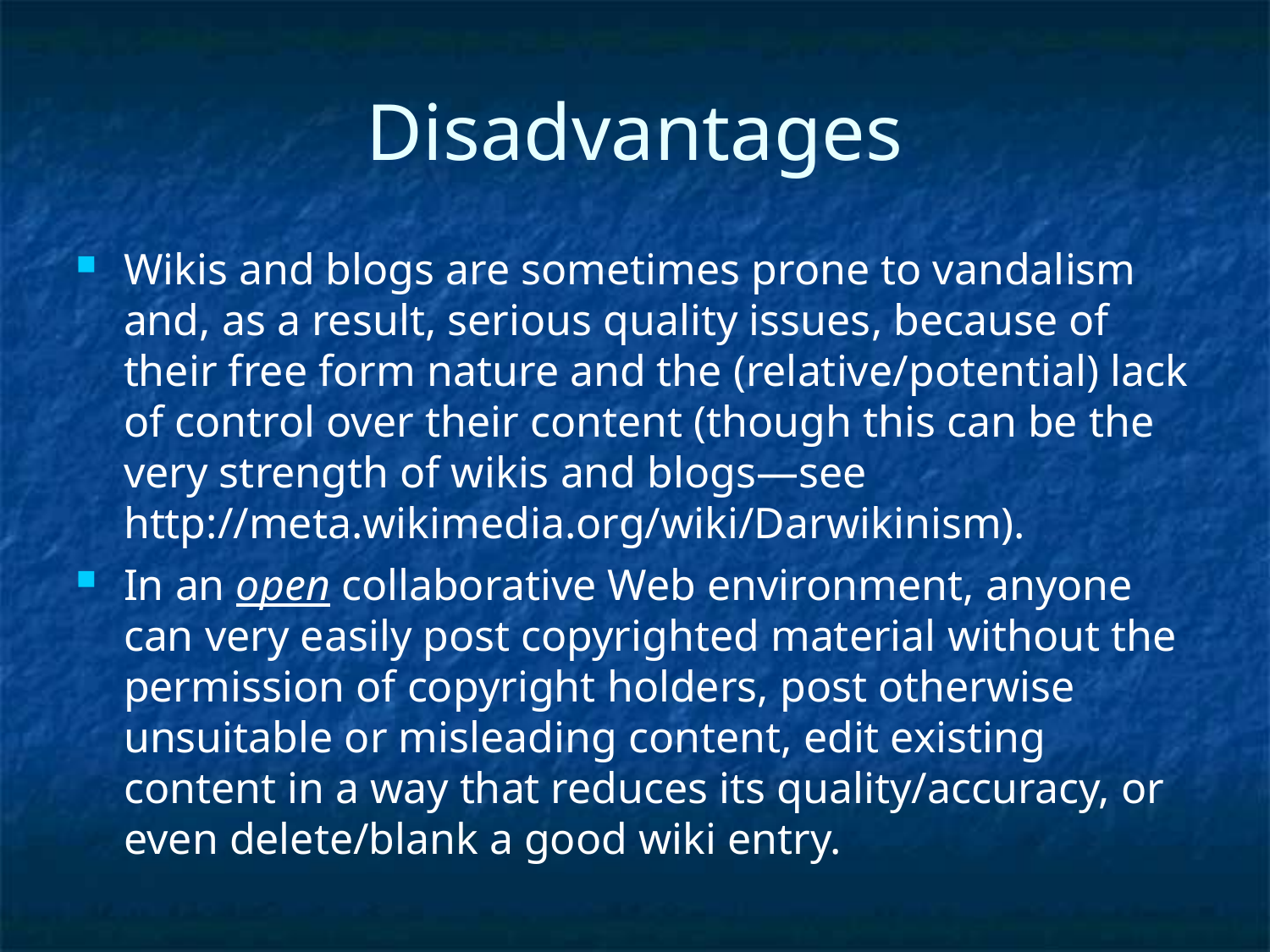

# Disadvantages
Wikis and blogs are sometimes prone to vandalism and, as a result, serious quality issues, because of their free form nature and the (relative/potential) lack of control over their content (though this can be the very strength of wikis and blogs—see http://meta.wikimedia.org/wiki/Darwikinism).
In an open collaborative Web environment, anyone can very easily post copyrighted material without the permission of copyright holders, post otherwise unsuitable or misleading content, edit existing content in a way that reduces its quality/accuracy, or even delete/blank a good wiki entry.

## Slide 44
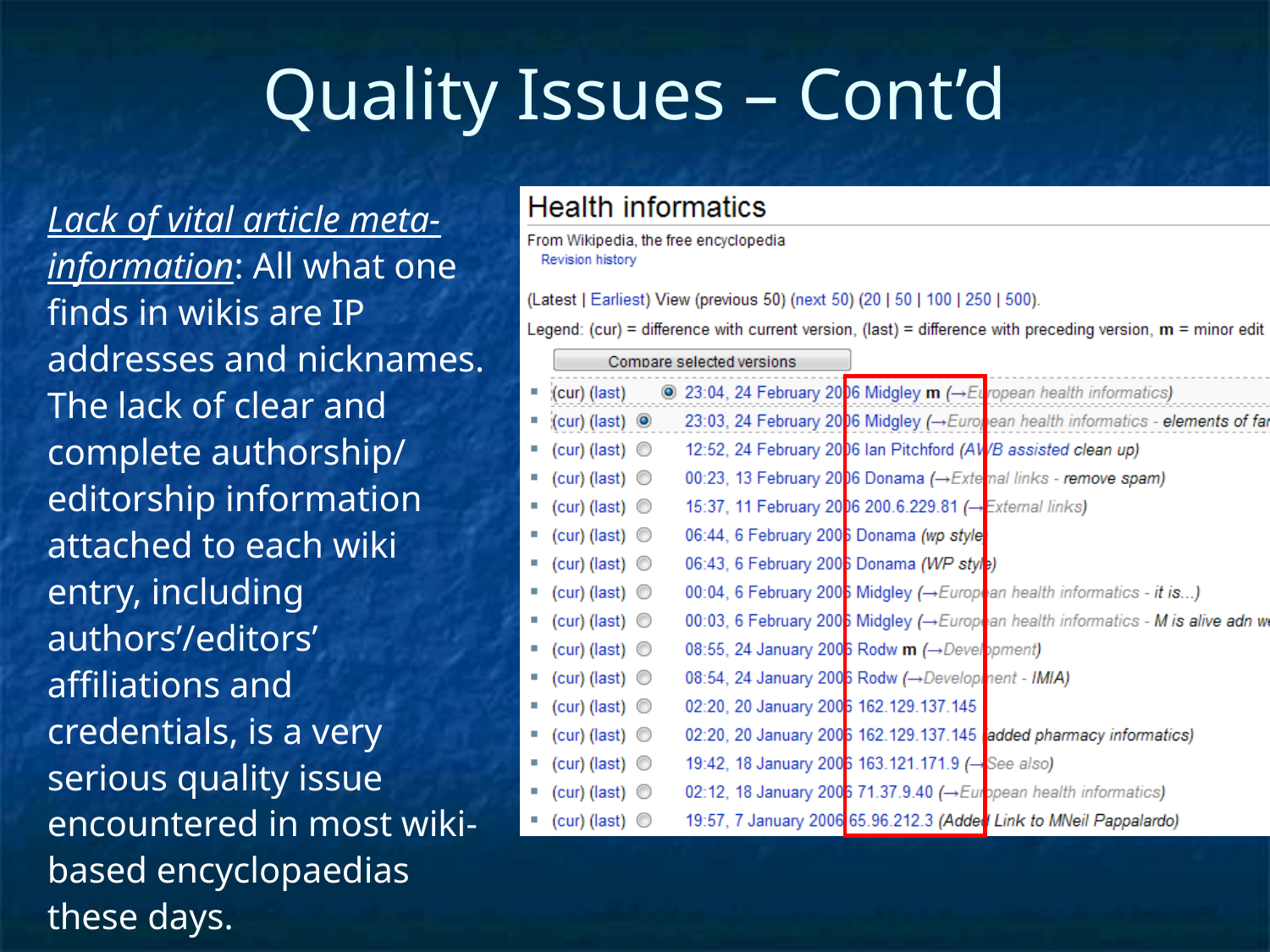

Quality Issues – Cont’d
Lack of vital article meta-information: All what one finds in wikis are IP addresses and nicknames. The lack of clear and complete authorship/ editorship information attached to each wiki entry, including authors’/editors’ affiliations and credentials, is a very serious quality issue encountered in most wiki-based encyclopaedias these days.

## Slide 45
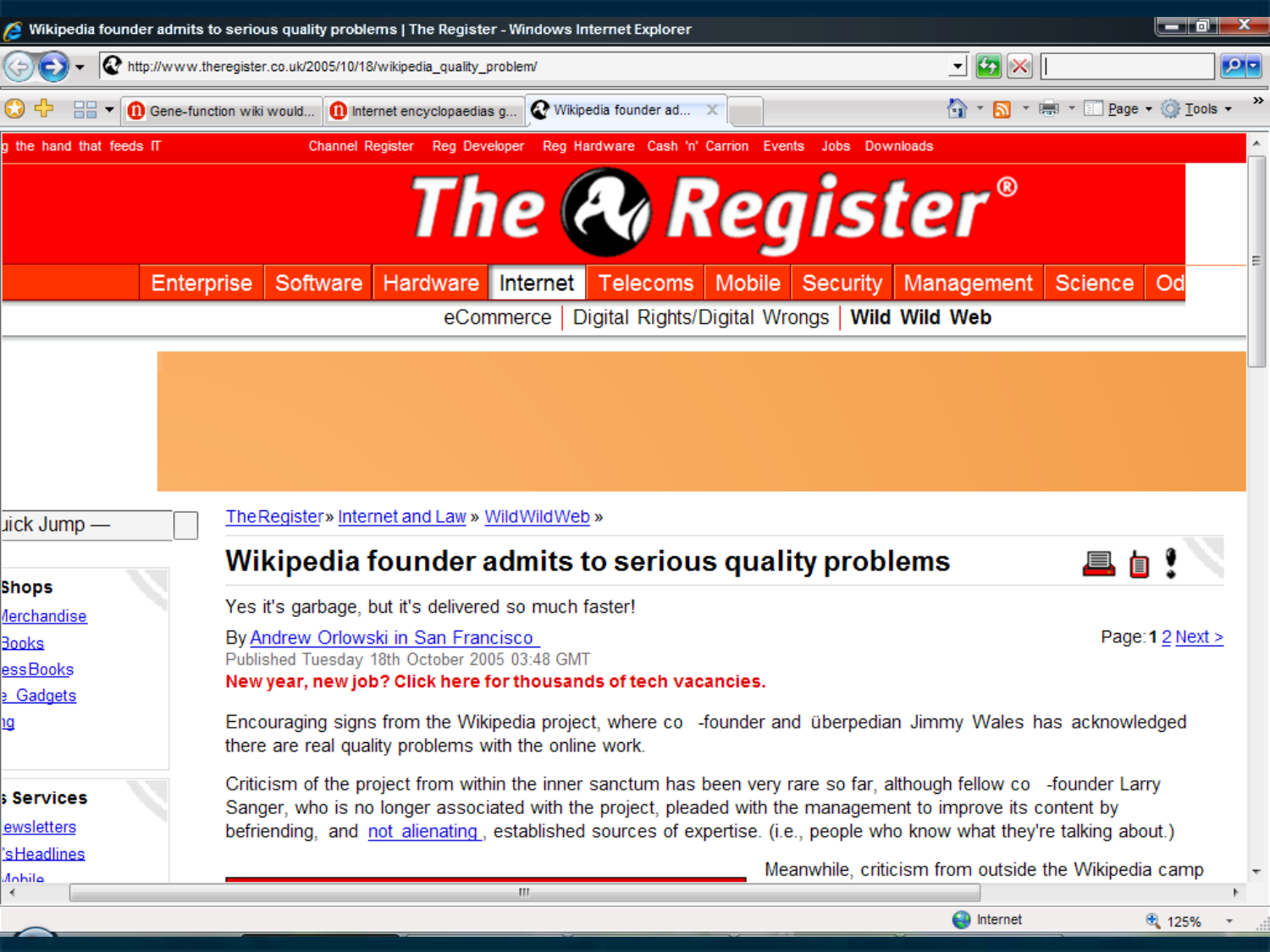

## Slide 46
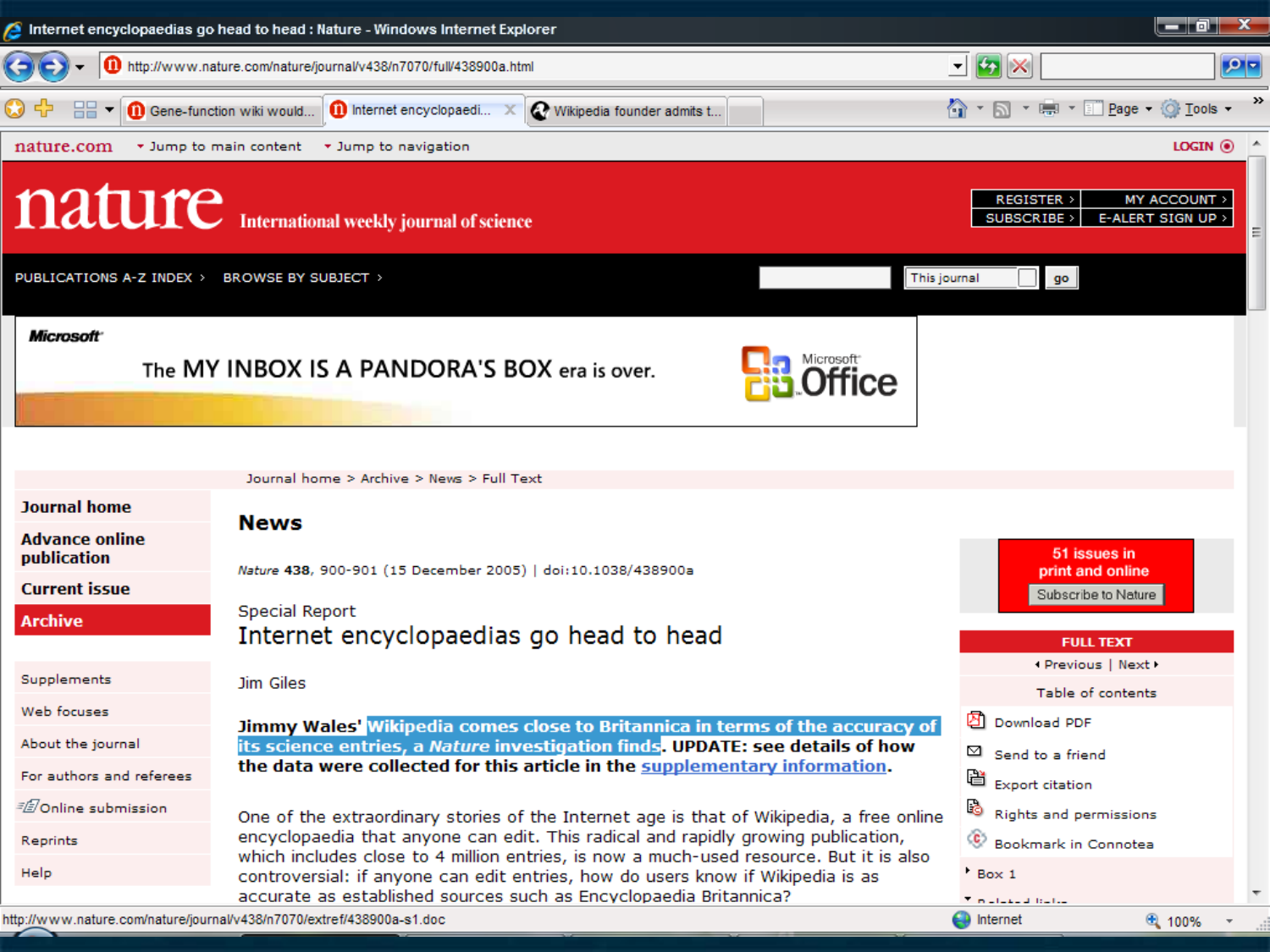

## Slide 47
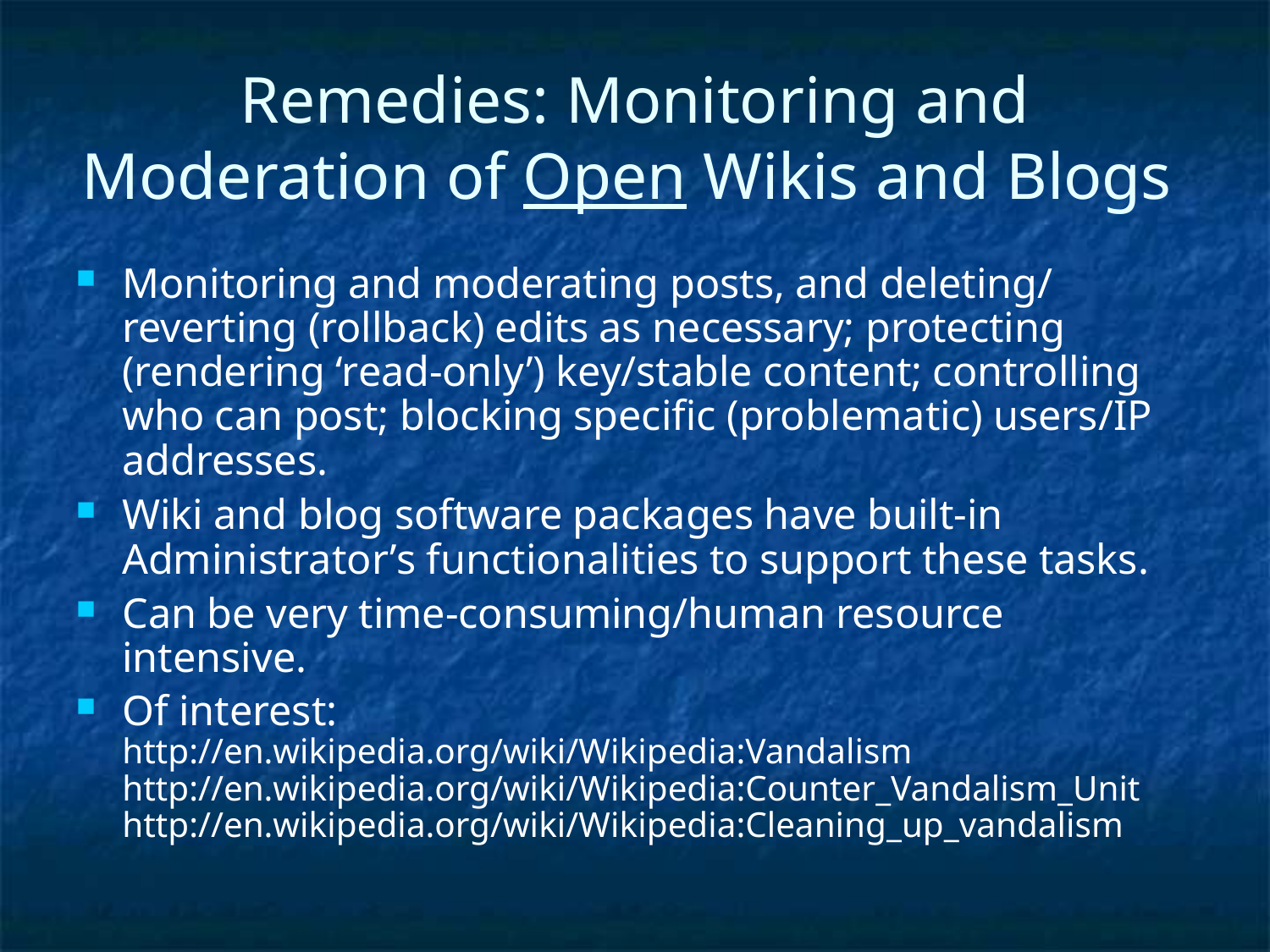

# Remedies: Monitoring and Moderation of Open Wikis and Blogs
Monitoring and moderating posts, and deleting/ reverting (rollback) edits as necessary; protecting (rendering ‘read-only’) key/stable content; controlling who can post; blocking specific (problematic) users/IP addresses.
Wiki and blog software packages have built-in Administrator’s functionalities to support these tasks.
Can be very time-consuming/human resource intensive.
Of interest:
http://en.wikipedia.org/wiki/Wikipedia:Vandalism
http://en.wikipedia.org/wiki/Wikipedia:Counter_Vandalism_Unit
http://en.wikipedia.org/wiki/Wikipedia:Cleaning_up_vandalism

## Slide 48
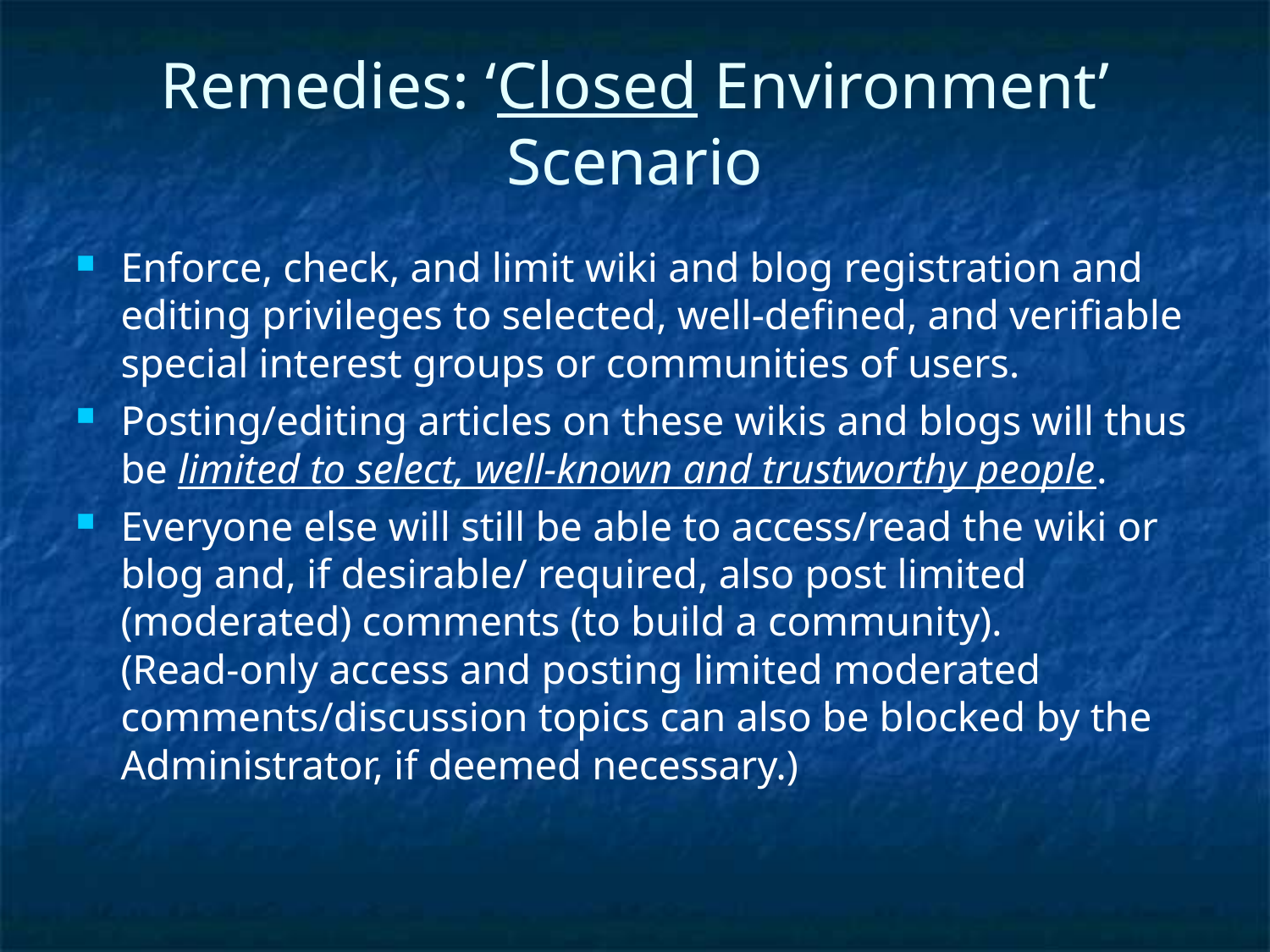

# Remedies: ‘Closed Environment’ Scenario
Enforce, check, and limit wiki and blog registration and editing privileges to selected, well-defined, and verifiable special interest groups or communities of users.
Posting/editing articles on these wikis and blogs will thus be limited to select, well-known and trustworthy people.
Everyone else will still be able to access/read the wiki or blog and, if desirable/ required, also post limited (moderated) comments (to build a community).
(Read-only access and posting limited moderated comments/discussion topics can also be blocked by the Administrator, if deemed necessary.)

## Slide 49
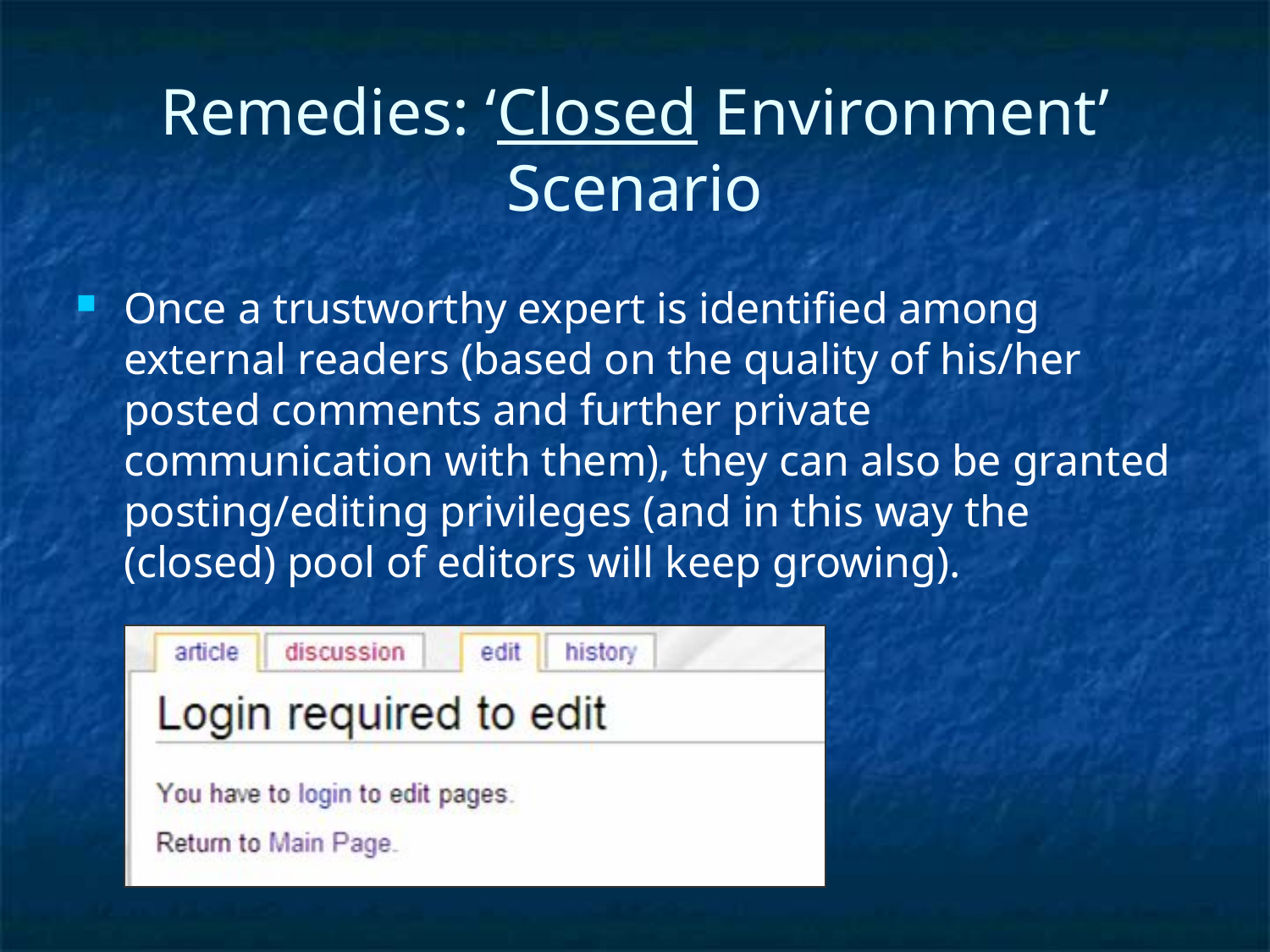

# Remedies: ‘Closed Environment’ Scenario
Once a trustworthy expert is identified among external readers (based on the quality of his/her posted comments and further private communication with them), they can also be granted posting/editing privileges (and in this way the (closed) pool of editors will keep growing).

## Slide 50
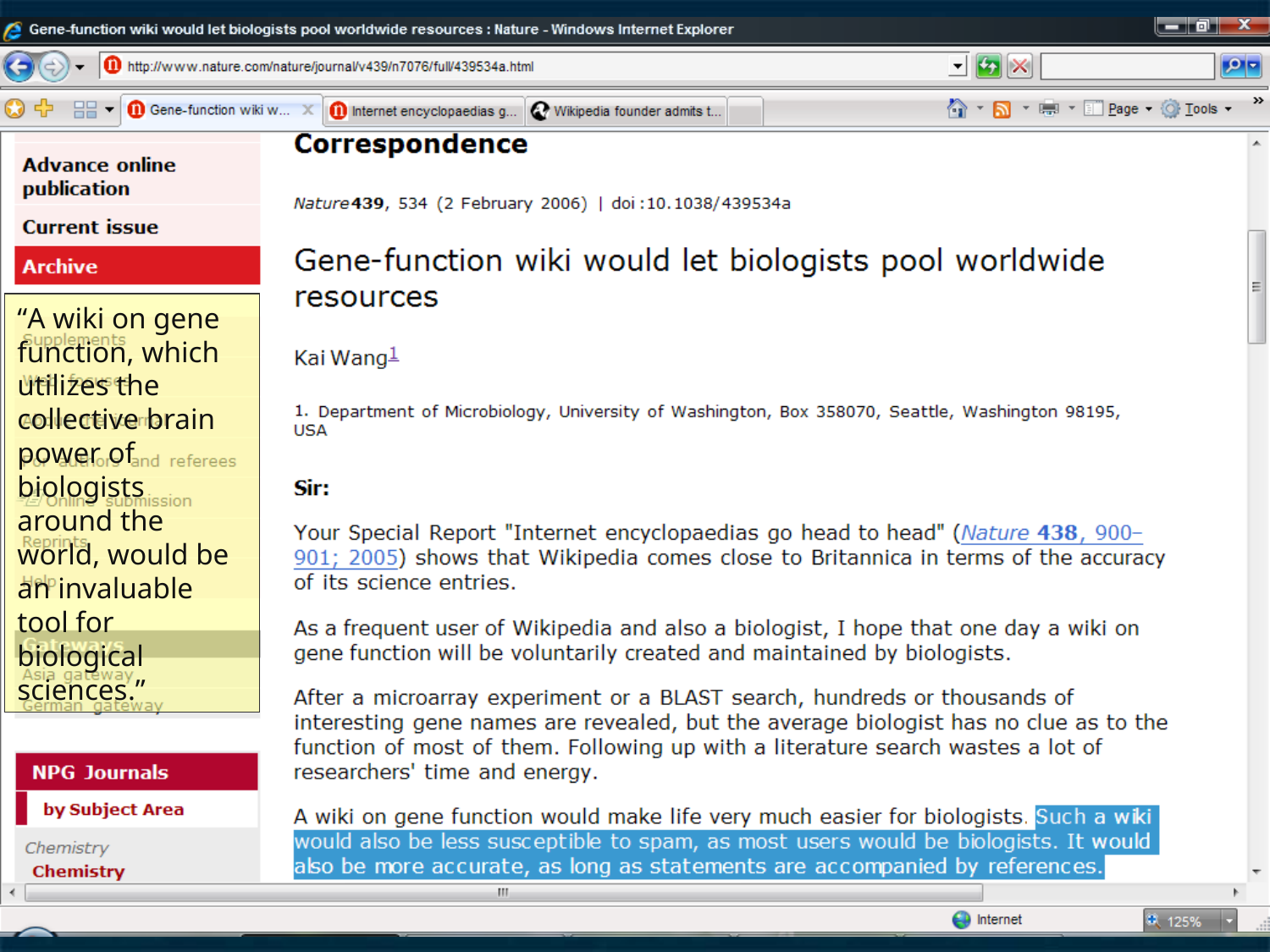

“A wiki on gene function, which utilizes the collective brain power of biologists around the world, would be an invaluable tool for biological sciences.”

## Slide 51
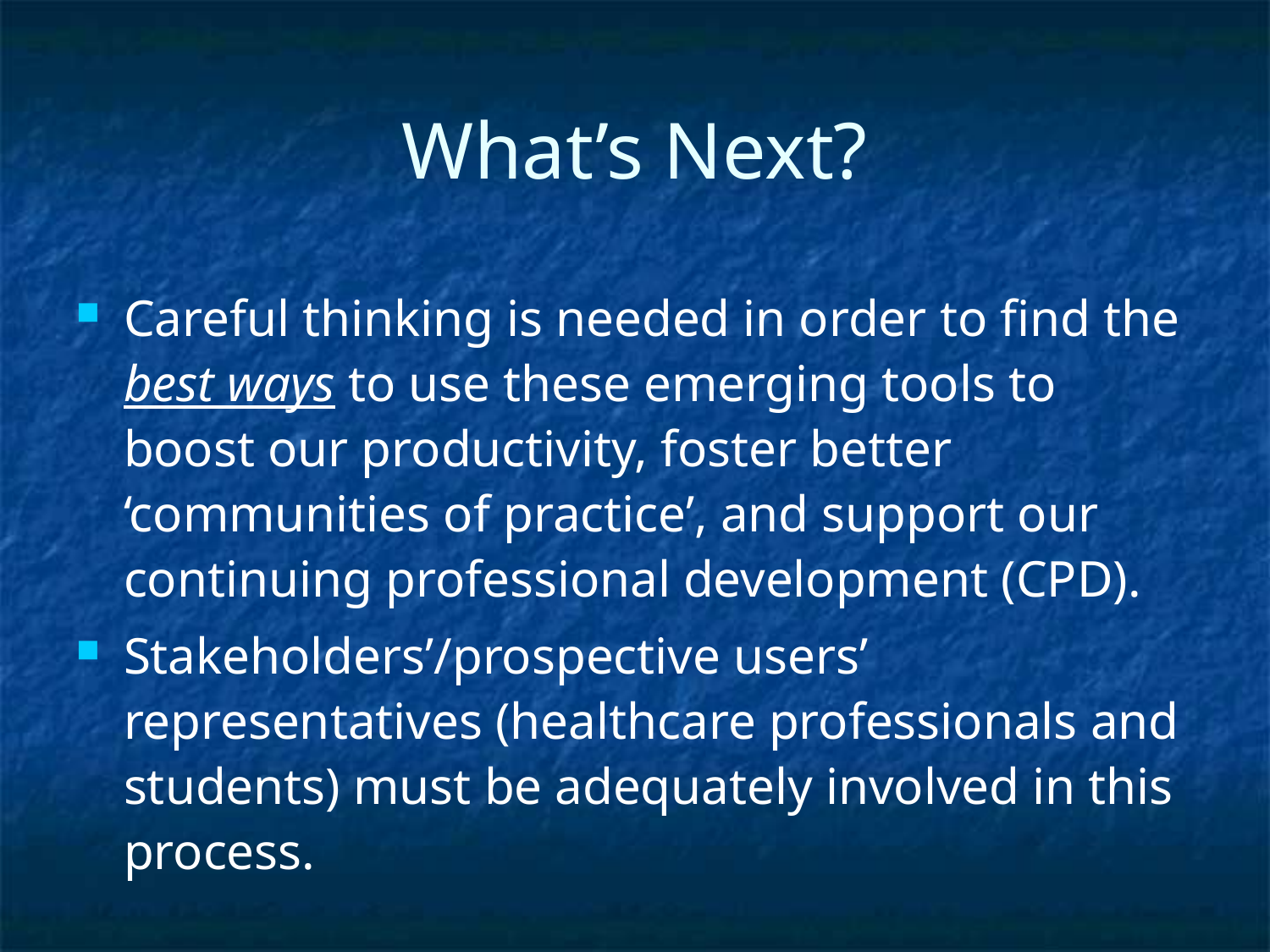

# What’s Next?
Careful thinking is needed in order to find the best ways to use these emerging tools to boost our productivity, foster better ‘communities of practice’, and support our continuing professional development (CPD).
Stakeholders’/prospective users’ representatives (healthcare professionals and students) must be adequately involved in this process.

## Slide 52
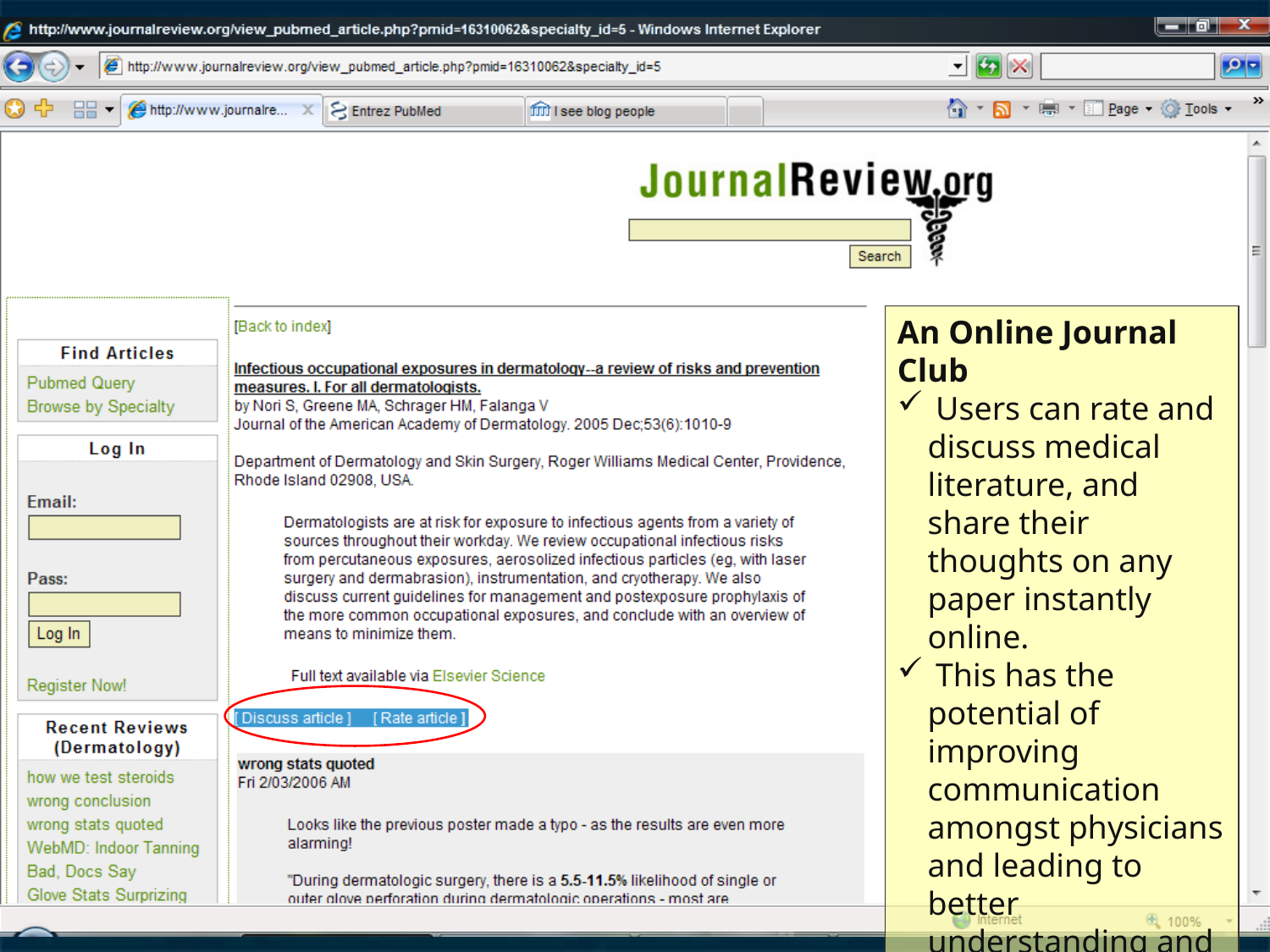

An Online Journal Club
 Users can rate and discuss medical literature, and share their thoughts on any paper instantly online.
 This has the potential of improving communication amongst physicians and leading to better understanding and interpretation of medical literature.

## Slide 53
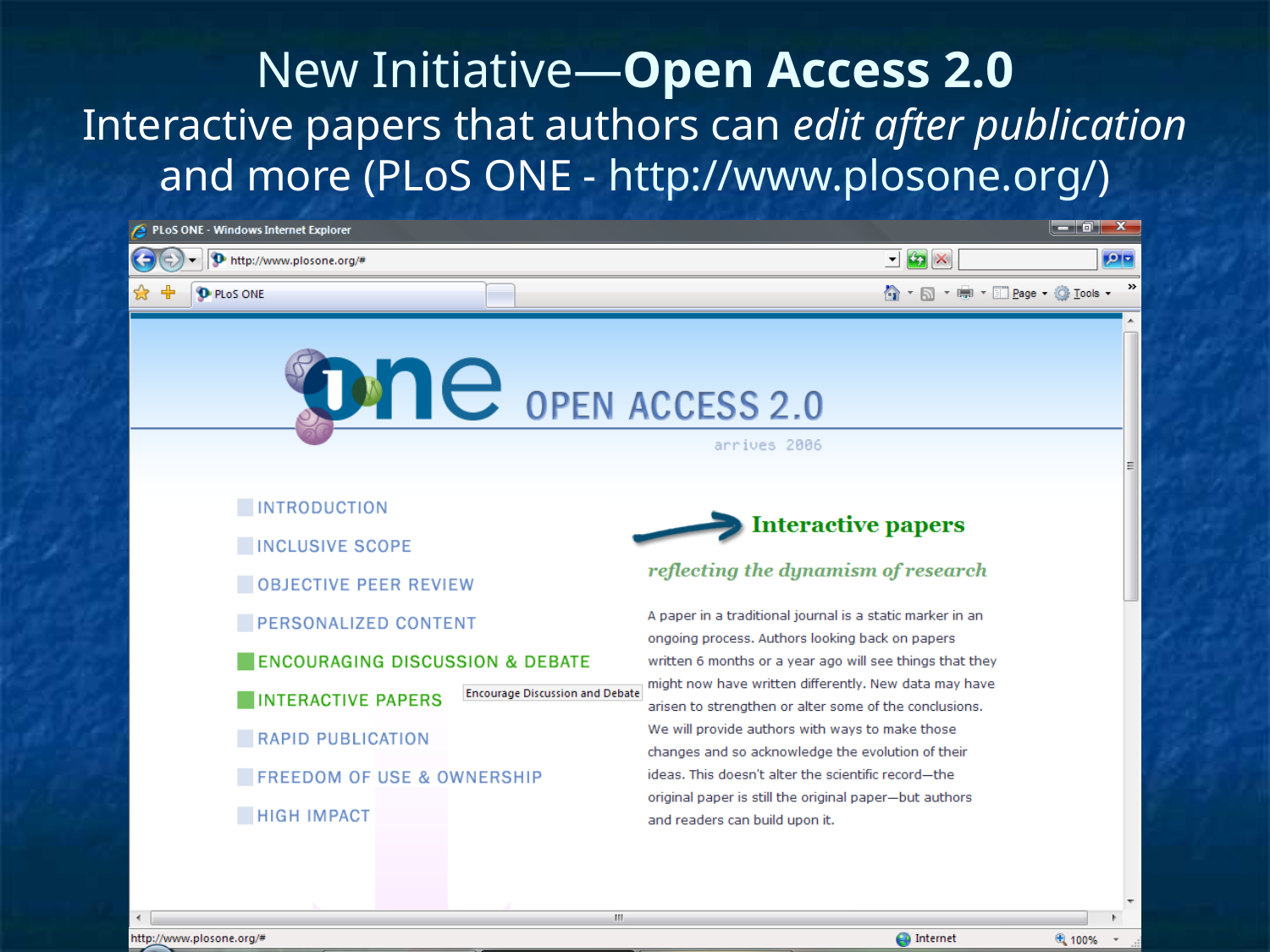

# New Initiative—Open Access 2.0 Interactive papers that authors can edit after publication and more (PLoS ONE - http://www.plosone.org/)

## Slide 54
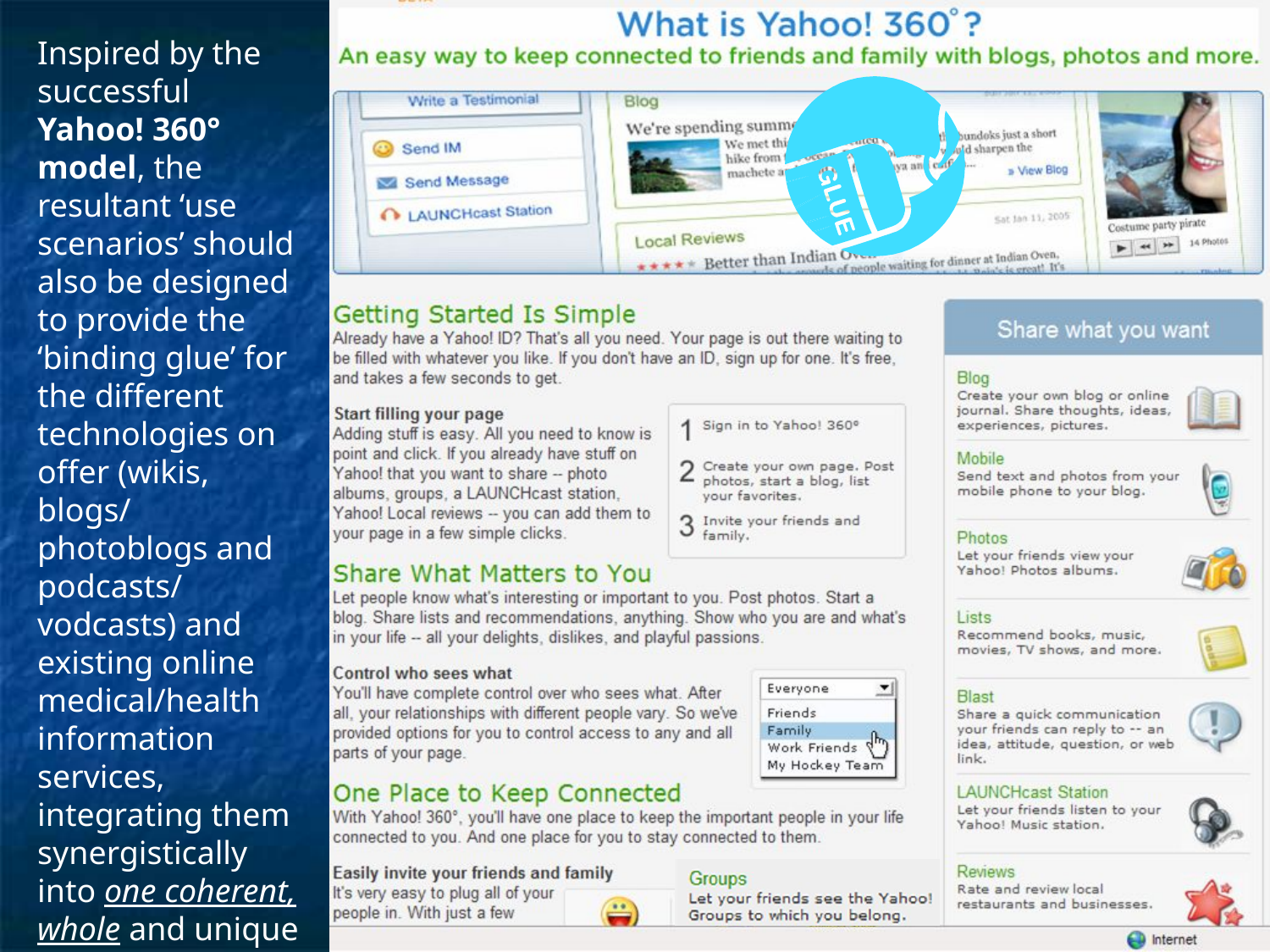

Inspired by the successful Yahoo! 360° model, the resultant ‘use scenarios’ should also be designed to provide the ‘binding glue’ for the different technologies on offer (wikis, blogs/ photoblogs and podcasts/ vodcasts) and existing online medical/health information services, integrating them synergistically into one coherent, whole and unique user experience.

## Slide 55
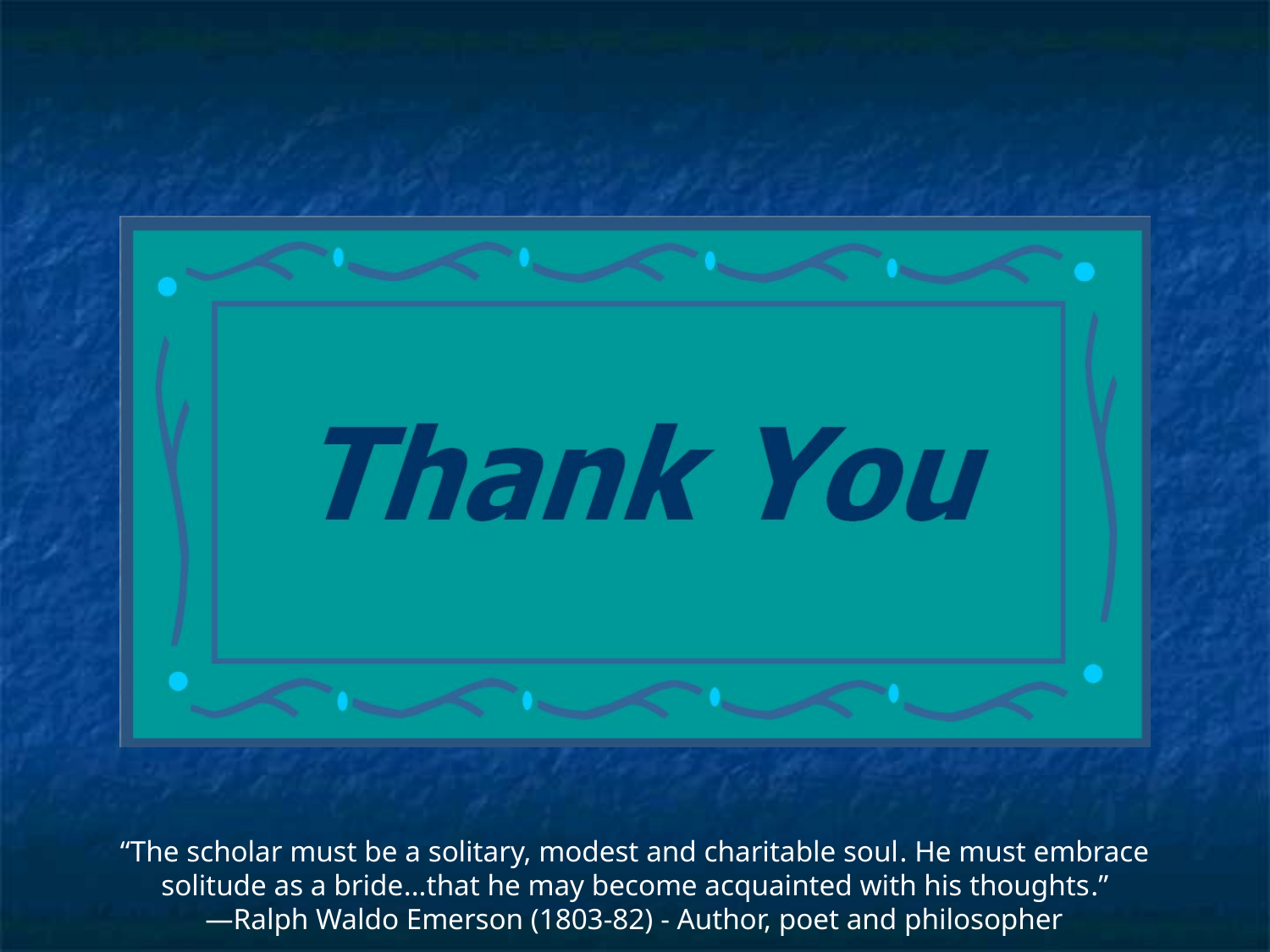

“The scholar must be a solitary, modest and charitable soul. He must embrace solitude as a bride...that he may become acquainted with his thoughts.”
—Ralph Waldo Emerson (1803-82) - Author, poet and philosopher
