# Supplementary material for: Wikis, blogs and podcasts: a new generation of Web-based tools for virtual collaborative clinical practice and education
Source: BMC Med Educ. 2006 Aug 15;6:41. doi: 10.1186/1472-6920-6-41 (PMC1564136; doi:10.1186/1472-6920-6-41)
Supplement: Additional File 2 — Web 2.0 tools: underpinning pedagogy, uses, and best practices in education. An illustrated PowerPoint PDF introducing the underpinning pedagogy of Web 2.0 tools, their uses and best practices in the context of higher education, together with a comprehensive Webliography about the subject. The presentation covers wikis, blogs, wikis vs. blogs, podcasts, and confluence of Web 2.0 tools, e.g., blikis and voice wikis (format: PDF). [file 1472-6920-6-41-S2.pdf]

Web compilation prepared by:
MN Kamel Boulos, MBBCh, MSc, PhD, ILTM, FRGS
mnkamelboulos@plymouth.ac.uk

WEB 2.0 TOOLS: UNDERPINNING PEDAGOGY, USES, AND BEST PRACTICES IN EDUCATION

June MMVI

Contents

- Wikis
- Blogs
- Wikis vs. blogs
- Podcasts
- Confluence of Web 2.0 tools (blikis and voice wikis)

Wiki uses in education

- Easily create simple Web sites
- Project development with peer review
- Group authoring
- Track a group project
- Data collection
- Review classes and teachers
- Tracking progress in your research group
- (And for instructors: collaborative curriculum design/course content authoring)
- Adapted from/read more at http://scienceofspectroscopy.info/edit/index.php?title=Using_wiki_in_education

Pedagogical potential and concerns of wikis, and guidelines for wiki use in education

- Quoted from Renée Fountain, **Wiki Pedagogy** (2005), URL: http://www.profetic.org:16080/dossiers/dossier_imprimer.php3?id_rubrique=110
  - Wikis may work best for knowledge building "over time" (through versions and groups)
  - ...progressive problem-solving (particularly open-ended problems) and even problem redefinition. For example, Wikis could work well for COP (communities of practice) whose goal is to develop solutions to common problems over time in order to improve practice
  - ...explaining increasingly diverse and contrary ideas, as well as examining the relatedness of ideas from diverse contexts
  - ...combining, synthesizing and evaluating definitions and terminology across disciplines
  - ...questioning underlying causes and principles
  - ...critically reading, and responding in a constructive and public way, to others' work
  - ...learning how to add both nuance and complexity to concepts in a given field, through systematic engagement and analysis with work produced by more advanced students, specialists and experts
  - ...learning to observe deeply, stereotype less, and avoid premature judgment

Pedagogical potential and concerns of wikis, and guidelines for wiki use in education – Cont'd

- Must read: Renée Fountain, **Wiki Pedagogy** (2005), URL: http://www.profetic.org:16080/dossiers/dossier_imprimer.php3?id_rubrique=110
  - Wikis maximize interplay
  - ...are democratic
  - ...work in real time
  - ...technology is text-based
  - ...permit public document construction, that is, distributed authorship
  - ...complicate the evaluation (grading) of writing
  - ...promote negotiation
  - ...permit the public to publish - Public as publisher
  - ...make feedback intensely public and potentially durable

Pedagogical potential and concerns of wikis, and guidelines for wiki use in education – Cont'd

- Must read: Renée Fountain, **Wiki Pedagogy** (2005), URL: http://www.profetic.org:16080/dossiers/dossier_imprimer.php3?id_rubrique=110
  - Wikis work on volunteer collaboration: "Wikis created in pedagogical contexts wherein collaboration is forced and enforced (since participation is often related to the student's grade) may prove to be a factor that determines why wikis may not work for some educators. That is, the voluntary aspect of wiki work, which involves an "opting in" to knowledge construction, may prove to be an essential and non-negotiable component of creative and sustainable participation"
  - ...enable complete anonymity
  - ...endorse particular ways of writing (e.g., NPOV - see http://en.wikipedia.org/wiki/Wikipedia:Neutral_point_of_view)
  - ...have no rules; non-interference with respect to creativity is high
  - ...require trust: "Building trust and enabling it in the digital domain, across differing and often divergent times and spaces, may prove to be too great a challenge to ask of educators who already lack time and resources" (see next slide on wiki vandalism)

Wiki vandalism

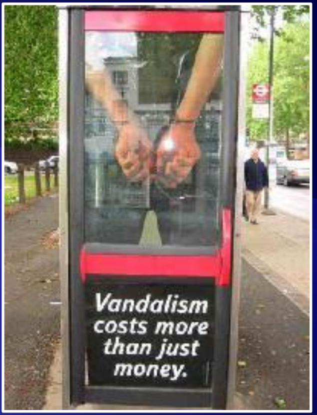

- There are two main types of vandalism—manual and automated (see http://www.orthodoxwiki.org/OrthodoxWiki:Vandalism). The latter is carried out using vandalbots (see http://meta.wikimedia.org/wiki/Vandalbot).
- A famous example of manual vandalism is the case of Wikipedia's false 'biography'of John Seigenthaler Sr.:
  http://en.wikipedia.org/wiki/John_Seigenthaler_Sr._Wikipedia_biography_controversy
  http://www.usatoday.com/news/opinion/editorials/2005-11-29-wikipedia-edit_x.htm
  http://news.bbc.co.uk/2/hi/technology/4502846.stm
- See also the discussion page of the 'Barney and Friends' Wikipedia article:
  http://en.wikipedia.org/wiki/Talk:Barney_%26_Friends#Semi-protect.3F
  and **Wikipedia:Counter-Vandalism Unit**
  http://en.wikipedia.org/wiki/Wikipedia:Counter-Vandalism_Unit

Wikis and plagiarism/copyright violation

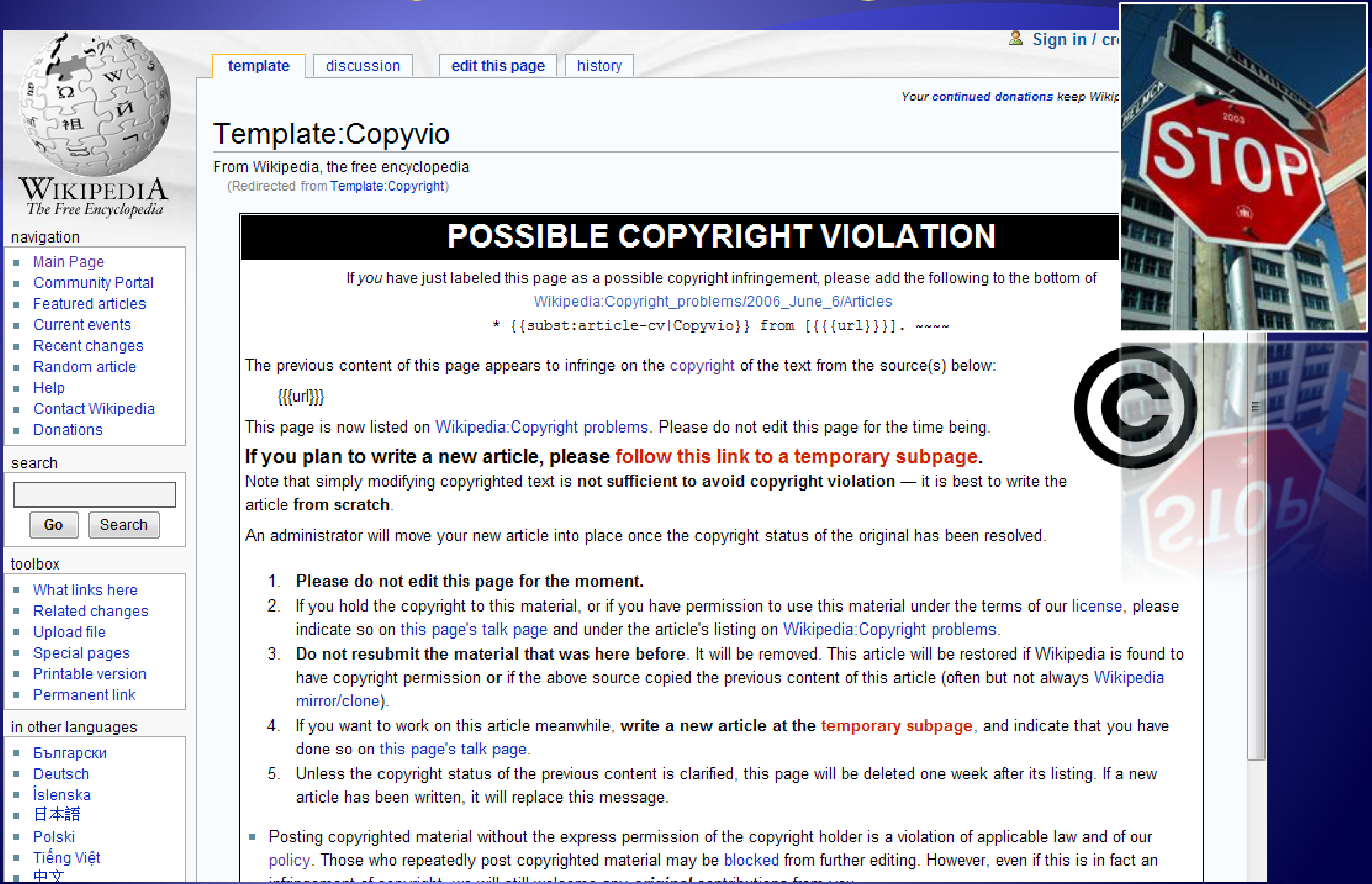

See **Wikipedia:Spotting possible copyright violations**
http://en.wikipedia.org/wiki/Wikipedia:Spotting_possible_copyright_violations

Wikis in education: Webliography

- Brian Lamb, **Wide Open Spaces: Wikis, Ready or Not**, Educause Review, 2004 - Excerpt: "In many respects, the wide-open ethic of wikis contrasts vividly with the traditional approaches of standard groupware and collaborative systems. Access restrictions, rigidly defined workflows, and structures are anathema to most wiki developers. What's unique about wikis is that users define for themselves how their processes and groups will develop, usually by making things up as they go along" - URL: http://www.educause.edu/pub/er/erm04/erm0452.asp
- Naomi Augar, Ruth Raitman and Wanlei Zhou, **Teaching and learning online with wikis**, Proceedings of the 21st ASCILITE Conference, 2004 - URL: http://www.ascilite.org.au/conferences/perth04/procs/augar.html
- Linda Schwartz, Sharon Clark, Mary Cossarin, Jim Rudolph, **Educational Wikis: features and selection criteria**, International Review of Research in Open and Distance Learning, 2004 - URL: http://www.irrodl.org/index.php/irrodl/article/view/163/244

Wikis in education: Webliography – Cont'd

- **My Brilliant Failure: Wikis In Classrooms** - URL: http://kairosnews.org/my-brilliant-failure-wikis-in-classrooms
- **TikiWiki for Education** (success stories > university level) – URL: http://edu.tikiwiki.org/tiki-index.php#_University_level_
- **WikEd** - URL: http://wik.ed.uiuc.edu/index.php/Main_Page

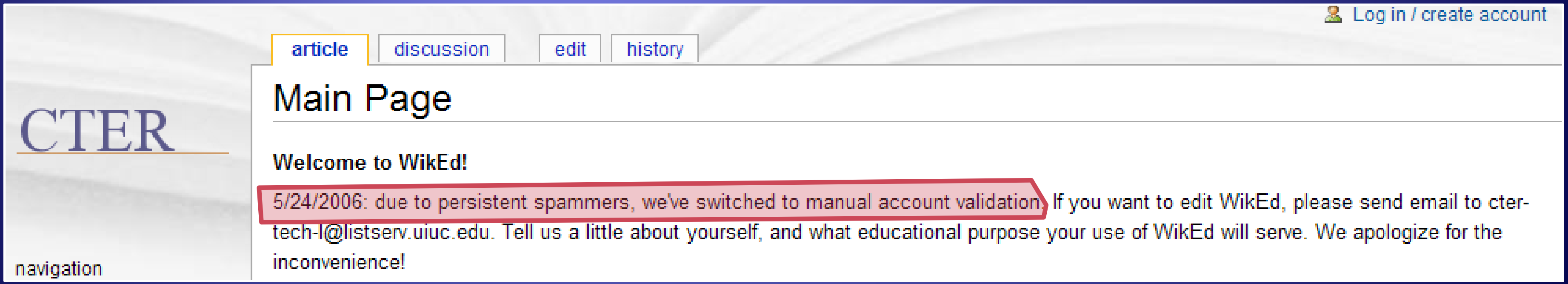

Blogs in education

Quoted from http://awd.cl.uh.edu/blog/

- **Options for <u>instructors</u> using blogs:**
  - Content-related blog as professional practice
  - Networking and personal knowledge sharing
  - Instructional tips for students
  - Course announcements and readings
  - Annotated links
  - Knowledge management
- **Options for <u>students</u> using blogs in your courses include:**
  - Reflective or writing journals
  - Knowledge management
  - Assignment submission and review
  - Dialogue for groupwork
  - E-portfolios
  - Share course-related resources

See also: **Why blogs?**
(*Ideas from various sources*)
http://www.weblogg-ed.com/why-weblogs

Blogs in education – Cont'd

Source: http://www.edtechpost.ca/gems/matrix2.gif

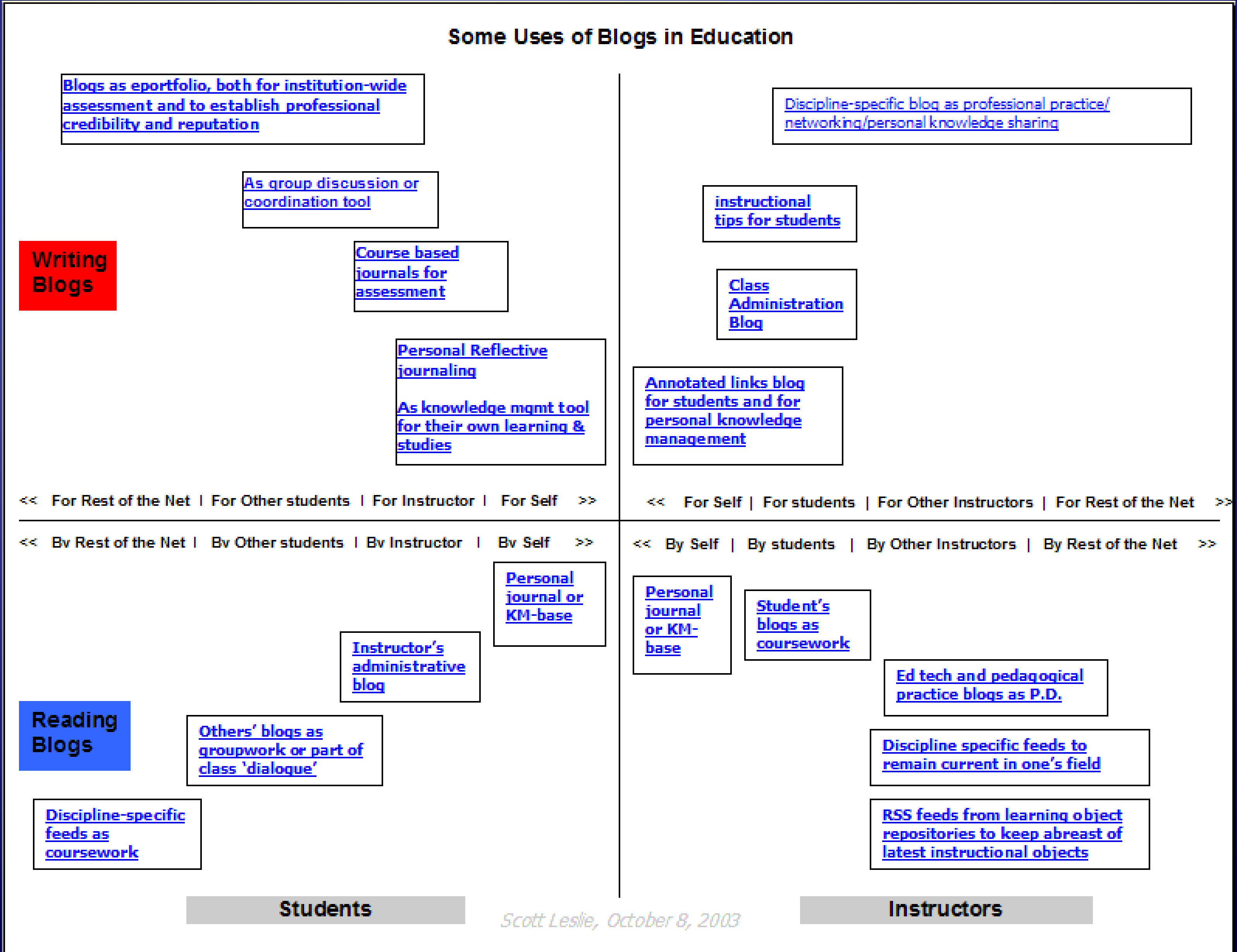

Blogs in education: Webliography

- Stephen Downes, **Educational Blogging**, Educause Review, 2004: Discusses the 'trend that is sweeping the world of online learning: the use of weblogs to support learning.' Asks interesting questions like 'what happens when a free-flowing medium such as blogging interacts with the more restrictive domains of the educational system?' - URL: http://www.educause.edu/pub/er/erm04/erm0450.asp?bhcp=1
- Richard E Ferdig and Kaye D Trammell, **Content Delivery in the 'Blogosphere'**, T.H.E Journal Online, 2004: Looks at the pedagogy behind blogs, gives practical suggestions for using blogs and discusses benefits of student blogging - URL: http://www.thejournal.com/the/printarticle/?id=16626
- **Blogs DOs and DONTs** - Excerpt: 'group blogs are a bad idea and don't work: sure there's a place for collaborative/group blogs but that place is not in education. Blogs work well for individuals' - URLs: http://blogsavvy.net/how-you-should-use-blogs-in-education and http://blogsavvy.net/how-not-to-use-blogs-in-education (based on **Blogs @ Anywhere: High fidelity online communication** - URL: http://incsub.org/blog/2005/blogs-anywhere-high-fidelity-online-communication)

Blogs in education: Webliography – Cont'd

- Janice Wendi Fernheimer and Thomas J Nelson, **Bridging the Composition Divide: Blog Pedagogy and the Potential for Agonistic Classrooms**, Currents in Electronic Literacy 2005 Fall; (9). Discusses the use of a single, multiply-authored class blog as the central interface for the writing classroom - URL: http://www.cwrl.utexas.edu/currents/fall05/fernheimernelson.html
- **Weblogs in the Classroom** - URL: http://www.eddept.wa.edu.au/cmis/eval/curriculum/ict/weblogs/
- **Blogs can help 'improve written communication skills'** - URL: http://bathsciencenews.blogspot.com/2006/02/mathematics-today-exploring.html
- **Blogging from Word 2007** - URL: http://blogs.msdn.com/joe_friend/archive/2006/05/12/595963.aspx

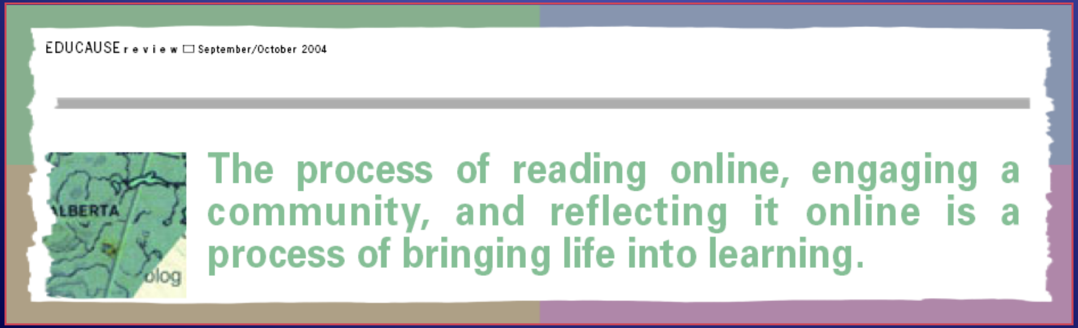

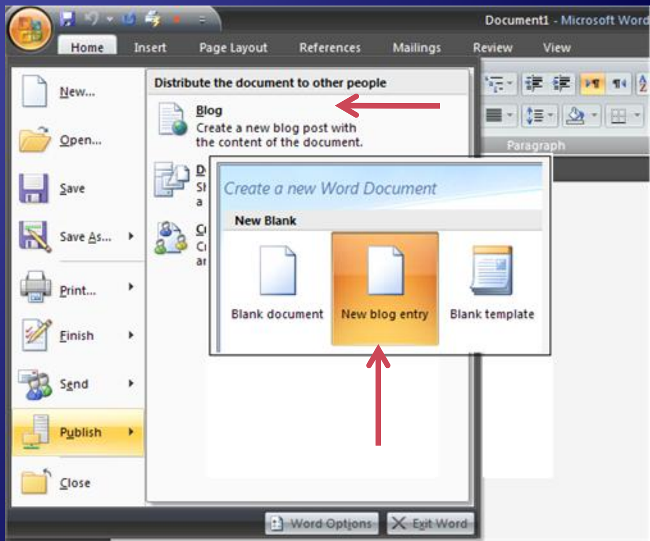

Wikis vs. blogs

- There are similarities and areas of overlap between wikis and blogs, but, by design, wikis are more suited than blogs for collaborative/group writing and editing, and also as content management tools, while blogs could be thought of as two-way conversations between an individual author and his/her audience.
- Quoting **Blogs and Wikis in Teaching at QUT - Update**, 5/5/2006, URL: http://snurb.info/index.php?q=node/472, "Both technologies can be useful tools in different teaching contexts, and it is simply important to make an informed choice as to which may be more appropriate for any one case. The key difference between them is usually the underlying organisation of information (temporal in blogs, spatial in wikis), and the answer to which one should be used can often be found right there already. So, blogs can be useful for ongoing personal/group reflection, or for the incremental development of skills/gathering of information/provision of feedback; wikis can be useful for compiling information and ideas in an *ad hoc* form, with informational structures emerging as information is being compiled."

Wikis vs. blogs – Cont'd

- However, this spatial/temporal differentiation between wikis and blogs might not be very accurate. For example, wikis (and their discussion or 'talk pages') have powerful data-and-time-stamped versioning capabilities, with many potential uses in a teaching and learning context. Wikis also feature RSS (Really Simple Syndication)/Atom-enabled special pages for 'recent changes', where changes/edits/posts to the wiki are listed chronologically. And in MediaWiki* wiki pages, editors can easily request the system to add a date-time stamp to whatever material they post (just type --~~~~).
  IBM wiki history flow is another (visual) evidence for the temporal and evolving nature/aspects of wikis: http://www.research.ibm.com/history/

  * A popular Open Source wiki engine: http://www.mediawiki.org/

Pedagogical podcasting or podagogy

Quoted from/read more at http://engage.doit.wisc.edu/podcasting/teachAndLearn/

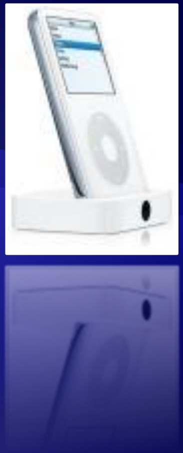

- Avoid overly complex and dense content material that includes lots of facts and figures—this is because most students listen to podcasts as they perform other tasks, e.g., riding a bus, driving, exercising, walking to class, etc. In most cases they won't be taking notes as they listen
- Recordings of classroom lectures may not be the best use of podcasting—only use lectures as podcasts when you have a strong pedagogical rationale for doing so
- Narrow the focus of a podcast

Requirements for successful podcasts in education

Quoted from/read more at http://www.xplanazine.com/xplanaradio/archives/2005/07/pedagogy_for_po.html

- Appropriate length
- Informal tone and high energy—to make students feel that they are part of something rather than merely the recipients of a boring presentation
- Context and review—educational podcasts should always begin with a clear context for the information being presented, and end with a review of the most important things to remember
- Options for different learning style preferences—effective podcasts should offer optional transcripts and/or images to address different learning styles as well as accessibility issues. Podcasts reviewed on the Web can make use of all these media types
- Flexible/multiple delivery options—to accommodate different students' preferences for subscribing and downloading

Podcasts and auditory learning style

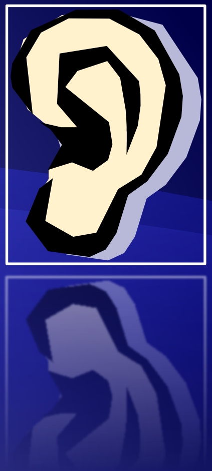

- A student with an auditory learning style component describes his experience: "Different people have different learning styles and strategies; some people like an auditory approach. (But) online classes lack the aural component; students have to read books or articles, write papers, and read and reply to topics on an online classroom discussion board, and there is no aural input. Podcasts are a big step towards bringing the aural element to distance education students who have an auditory learning style." Read more at http://kennykimchee.blogspot.com/2006/02/podcasts.html

Podcasts and auditory learning style – Cont'd

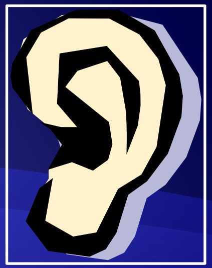

- Another blogger writes: "Podcasting gives me "sovereignty over text" (an academic term for consumer control and choice) and overcomes the linear access limitations of the broadcast media and gives me the type of random access print and the Internet provide, which is a major reason why the Internet will slowly replace the linearly accessed broadcast and cable media unless radio, television, and cable can provide random access in the form of podcasting, video on demand, and tivoing. (It's interesting that tivoing is becoming a generic phrase just as xeroxing became a generic phrase for copying paper, now it's copying programming, but it's the same concept.) **I found that listening to audio books is in many ways more satisfying for me than reading a book, but perhaps that's because I have an auditory learning style. But what this means is that I can now choose a content input system that matches my learning style**. Is technology great, or what?" Read more at http://www.mediacurmudgeon.com/archives/2005/04/000183print.html

Podagogy: Webliography

- **Podcast lectures for uni students**, BBC News, 2006: A Bradford University microbiology lecturer abolishes traditional lectures for students in favour of podcasts – URL: http://news.bbc.co.uk/go/em/fr/-/2/hi/uk_news/england/west_yorkshire/5013194.stm (Video: http://news.bbc.co.uk/media/avdb/news_web/video/9012da680043ec8/bb/09012da68004424f_16x9_bb.asx)
- Rebecca Knight, **Podcast pedagogy divides opinion at US universities**, Financial Times, 2006 - URL
- http://news.ft.com/cms/s/904272e4-9997-11da-a8c3-0000779e2340.html
- **Pod Pedagogy (blog)** – URL: http://www.bio.purdue.edu/mt/feeds/pod_pedagogy/
- **Podagogy (blog)** – URL: http://blog.podagogy.com/
- **Stanford on iTunes U** (podcasting example in higher education) – URL: http://itunes.stanford.edu/

Confluence for synergy and convenience

- Confluence: a flowing together of two or more streams; the point of juncture of such streams; the combined stream formed by this juncture
- Synergy: the interaction of two or more agents or forces so that their combined effect is greater than the sum of their individual effects
- Convenience: the quality of being suitable to one's comfort, purposes, or needs; personal comfort or advantage: services that promote the customer's convenience; something that increases comfort or saves work

Definitions from http://www.answers.com/

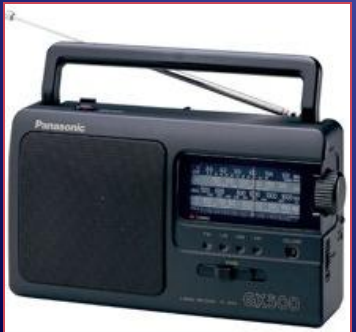

+

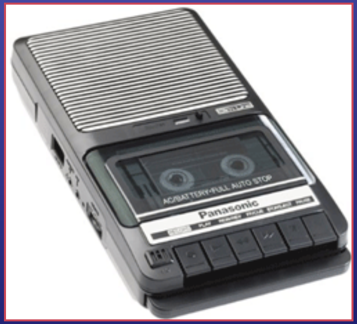

=

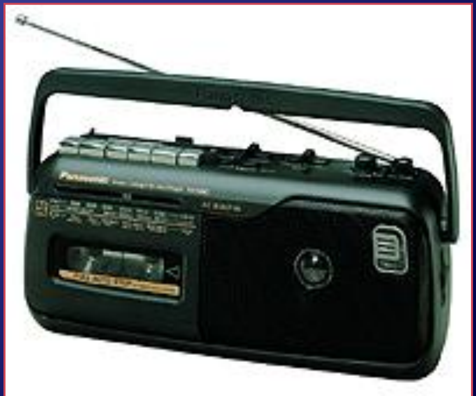

Confluence of Web 2.0 tools

- **Blikis**: "a Bliki (also known as a WikiLog, Wog, WikiWeblog, Wikiblog, or Bloki), is a blog with wiki support" (see http://en.wikipedia.org/wiki/Bliki)
- See also:
  - **Blikis - The Next Big Thing** http://www.mguerrilla.com/media_guerrilla/2004/06/blikis_the_next.html
  - **Wikis + Blogs = SocialText** http://www.intranetjournal.com/articles/200507/ij_07_29_05a.html
  - **Cardboard - a bliki (blog+wiki)** http://blog.kowalczyk.info/archives/2004/06/01/cardboard-a-bliki-blogwiki and http://sourceforge.net/projects/cardboard
  - **More on blikis** http://blog.contentious.com/archives/2004/06/23/a-new-wikibliki-movement-afoot and http://tikiwiki.org/tiki-index.php?page=Bliki

Confluence of Web 2.0 tools

- **Voice and podcasting-enabled wikis**: in 2006, Boulos *et al.* proposed developing 'a novel voice and podcasting-enabled Open Source wiki for collaborative teaching and learning' (see their ESRC grant proposal at http://healthcybermap.org/publications/MNKB_VoiceWiki_ESRC-EPSRC_TEL-Call_submission.zip)
- **The voice advantage:** voice has always been a much more natural and realistic means of human communication than writing (displayed text). Its advantages include being more easily comprehended by "poor" readers, not distracting visual attention from concurrent stimuli such as diagrams, being more lifelike and therefore more engaging, and being good for conveying temporal information. In an experimental study involving 141 college students, 82 percent of subjects preferred text and voice to text-only (or voice-only) information delivery methods (see Shih Y-F, Alessi SM: Effects of Text Versus Voice on Learning in Multimedia Courseware. Journal of Educational Multimedia and Hypermedia 1996, 5(2):203-218)
- **The collaborative advantage:** the opportunity for nurturing critical reflection and discussion skills among learners (thanks to the collaborative features of wikis and their discussion/talk pages, which would also be voice enabled in our proposed voice wiki) are irresistible and offer a significant step further than the simple podcasting of lecture material, which alone is deficient/lacks interaction with other tutees/tutors

THANK YOU
